# Mapping digital public health training: are we preparing the European workforce?

**DOI:** 10.3389/fpubh.2026.1778953

**Published:** 2026-02-11

**Authors:** Aldo Gorga, Chiara Barbati, Margarita Airapetian, Giovanni Leonardo Briganti, Erik Decio Carnevali, Francesco Cervellera, Giuseppa Granvillano, Vittorio Grieco, Francesco Leonforte, Damiano Peretti, Lorenzo Ramondetti, Lorenzo Prisciano, Eleonora Raso, Pier Paolo Russo, Giuseppe Vella, Francesco Baglivo, Caterina Rizzo, Anna Odone

**Affiliations:** 1 Department of Sciences of Public Health and Pediatrics, University of Turin, Turin, Italy; 2 Department of Public Health, Experimental and Forensic Medicine, University of Pavia, Pavia, Italy; 3 Department of Sciences of Public Health, Hygiene and Preventive Medicine, University of Genova, Genova, Italy; 4 Department of Medical and Surgical Science, Alma Mater Studiorum, University of Bologna, Bologna, Italy; 5 Department of Medicine and Surgery, University of Perugia, Perugia, Italy; 6 Public Health Physician, University Hospital Polyclinic “G.Rodolico-San Marco”, Catania, Italy; 7 Department of Medical, Surgical Sciences and Advanced Technologies, University of Catania, Catania, Italy; 8 Department of Medical Sciences and Public Health, University of Cagliari, Cagliari, Italy; 9 Department of Health Promotion, Maternal and Infant Care, Internal Medicine and Medical Specialties (PROMISE), University of Palermo, Palermo, Italy; 10 Department of Preventive Medicine, Azienda Sanitaria Provinciale di Palermo, Palermo, Italy; 11 Department of Translational Research and New Technologies in Medicine and Surgery, University of Pisa, Pisa, Italy; 12 Italian Society of Artificial Intelligence in Medicine (SIIAM, Società Italiana Intelligenza Artificiale in Medicina), Rome, Italy; 13 Medical Direction, Fondazione IRCCS Policlinico San Matteo, Pavia, Italy

**Keywords:** artificial intelligence, digital health, digital public health, education, public health, workforce

## Abstract

**Introduction:**

Digital transformation and artificial intelligence are reshaping public health practice, yet the extent to which the workforce is being prepared for these changes remains unclear. This mapping review aimed to characterize digital public health training initiatives targeting public health professionals in Europe.

**Methods:**

Training initiatives delivered between January 2020 and December 2025 were systematically identified across five European countries (Italy, Germany, France, Spain, United Kingdom) and major international organizations. Sources included websites, archives, and social media of public health associations, schools of public health, and health organizations. Systematic searching was complemented by direct institutional contact. Initiatives were classified by format, provider, and thematic content using domains from the ASPHER Core Curriculum framework.

**Results:**

A total of 367 training initiatives were identified. Activity increased sharply after 2022, with over half of all initiatives (56.7%) delivered in 2024-2025 alone. Conference sessions (54.5%) and webinars (22.3%) predominated, while structured courses (18.3%) and degree programmes (3.3%) were less common. Scientific associations delivered most initiatives (69.5%), with academic institutions accounting for less than one-third (30.5%). International organizations contributed to nearly one-third of all initiatives (32.4%). Thematic content focused primarily on digital tools (66.8%) and leadership for digital transformation (53.1%), whereas training on the infosphere and health misinformation was notably underrepresented (2.5%).

**Conclusion:**

Digital public health training for the European workforce is expanding rapidly but remains fragmented, dominated by short formats, and insufficiently integrated into academic curricula. Strengthening formal educational pathways and addressing content gaps will be essential to build sustainable digital competencies across the profession.

## Introduction

The digital transformation of health systems, accelerated by the rapid diffusion of Digital Health (DH) tools and growing interest in Artificial Intelligence (AI), is increasingly recognized as a structural and organizational opportunity to reshape how public health (PH) is governed, delivered, and evaluated (1, 2). Within this evolving landscape, the notion of digital public health (DPH) has been described in various ways, but definitions tend to converge on the general concept of integrating digital technologies into PH theory and practice to support PH goals (3–5). Rather than representing the simple adoption of new technologies, digital transformation in PH entails system-level changes affecting decision-making processes, workforce roles, and institutional capacities, with direct implications for PH performance and sustainability (6, 7). This includes established DH tools, as well as emerging technologies such as AI, with growing relevance for surveillance, risk assessment, modeling, and policy support (8, 9).

In this context, empowering the PH workforce to engage effectively with DH tools and data-driven processes has emerged as a central priority for health systems (6, 7) as the capacity to benefit from digital transformation largely depends on the skills of the workforce responsible for interpreting, governing, and applying these technologies (9). Complicating the picture, the core PH workforce as intended in common frameworks is diverse, comprising professionals whose primary functions lie in PH and spanning roles in epidemiology, surveillance, health protection, data analysis, communication, governance and policy development (10–14). A growing body of evidence indicates that the diffusion of DH technologies involves substantial changes in required competencies across health professions, underscoring the need for continuous upskilling and structured workforce development strategies (15), and extending beyond mere technical skills to include data interpretation, critical appraisal, and responsible use of digital systems (15).

PH professionals, who are closely involved in activities increasingly shaped by digital processes, are now expected to demonstrate digital literacy and eHealth literacy, particularly as limited competencies in this sense may constrain the effective use of digital tools and contribute to inequities in access, quality, and system performance (16, 17). Additionally, they need to be able to interpret complex data, understand how digital systems operate, predict ethical and regulatory issues, communicate effectively in digital information environments, lead digital change, manage health data responsibly, control and regulate digital processes and use emerging technologies safely and transparently (7, 18, 19). From a governance and leadership perspective, the digital transformation of PH raises critical challenges related to accountability, regulation, and organizational change (15). Analyses of DH governance stress the need for public institutions to develop internal competencies that support ethical oversight, coordination across sectors, and responsible adoption of digital technologies (15). At the same time, PH leaders are expected to guide and monitor AI-driven transformation processes, balancing innovation with equity, workforce sustainability, and public trust (16, 17).

In response to these challenges, several frameworks and educational initiatives have been proposed to define and strengthen DH competencies within the PH workforce. The Association of Schools of Public Health in the European Region (ASPHER) Core Curriculum identifies digital transformation as a cross-cutting domain of PH education, calling for the integration of digital competencies into formal training pathways (18). Complementary consensus-based frameworks and scoping reviews further emphasize the need for competency-based approaches to DH education, particularly for professionals engaged in population-level functions (20, 21). However, despite growing recognition of these needs, current educational responses remain fragmented (22, 23). Reviews of DPH education programmes indicate that many initiatives are short-term, heterogeneous in content, and poorly aligned with professional roles and real-world PH functions (22, 23).

Against this background, a systematic mapping of existing DPH training initiatives is needed to better understand the current educational landscape (6, 24). Such an approach can help identify gaps, overlaps, and opportunities for more coherent capacity-building strategies to integrate DH competencies into PH education, governance, and practice. Thus, this study aims to analyse DPH training initiatives delivered between 2020 and 2025 in five European countries, with a view to characterizing their availability, distribution, formats, providers and thematic content areas.

## Materials and methods

### Study design and objectives

This study adopted a mapping review approach to systematically identify and characterize DPH training initiatives delivered in five OECD European countries (Italy, Germany, France, Spain, and the United Kingdom) between 1 January 2020 and 31 December 2025. These countries were selected to ensure broad European coverage while matching the language competencies available within the research team. The objective was to provide a comprehensive description of the current DPH training landscape by assessing the availability of initiatives, their distribution across countries and types of providers, formats and addressed thematic content domains.

### Eligibility criteria

Training initiatives were included if they consisted of structured educational activity focused on DPH, understood as the use of digital technologies to support or enhance PH functions (3–5). Within this broad definition, the review considered both stand alone programmes explicitly centered on DPH topics, such as training courses, webinars, seminars, workshops, and seasonal schools, digital components embedded within wider PH curricula, including lecture series, and degree modules, and dedicated thematic sessions within scientific conferences. Eligible initiatives targeted PH professionals, according to the ASPHER tripartite classification of the PH workforce (11, 14), focusing on individuals for whom PH activities constitute a primary component of their professional role. This group includes graduates of bachelor's or master's programmes in PH, physicians and other health professionals with PH specialization, as well as professionals engaged in sustained PH practice at a relevant level of expertise (11, 14). Training initiatives were therefore eligible when they addressed this core workforce at different stages of education and professional development, encompassing formal graduate and postgraduate pathways (Master of Public Health-MPH-degrees and residency/specialization schools), as well as ongoing continuing professional development activities designed for PH practitioners and researchers.

Initiatives had to be delivered or scheduled between 1 January 2020 and 31 December 2025, and to be supported by verifiable documentation. Eligible sources of documentation included webpages, programme descriptions, flyers, conference pages, reports, and social media announcements. If such information was incomplete or not publicly available, confirmation obtained through direct contact with training providers was also accepted. Initiatives offered by providers based in the five target countries or by international organizations operating in Europe were considered, as defined below.

Initiatives were excluded if they were aimed exclusively at professional groups outside the PH practice as defined above, or if they consisted solely of informational or scientific materials without an educational component.

### Information sources and search strategy

Training providers were identified *a priori* across three macro-categories: i) National Public Health Associations, identified primarily through the European Public Health Association (EUPHA) membership list and supplemented by other relevant national scientific or professional associations; ii) Schools and Academic Institutions of PH, including universities and departments offering PH specialization programmes, residency training, or master's degrees in PH in the target countries, identified through relevant national association websites and official registries; and iii) International and National Health Organizations, including the WHO Regional Office for Europe, the European Center for Disease Prevention and Control (ECDC), the European Health Management Association (EHMA), national PH institutes, and other relevant networks. The complete list of providers searched is available in Supplementary Table S1.

Gray literature sources were systematically searched and included official institutional websites (sections on training, events, news, publications, and archives), official social media profiles (LinkedIn, Twitter/X, Facebook), newsletters, brochures, annual reports, and event archives from past conferences and workshops.

To complement the targeted institutional search, a systematic open web search was conducted using two different search engines (Google and DuckDuckGo). Predefined search strings were developed and translated into the five study languages (English, Italian, German, French, and Spanish), combining terms related to DH, AI, and PH training (Supplementary Table S2). For each search string, the first 30 pages of results were screened.

To validate gray literature findings and address information gaps, a direct contact phase was implemented. A standardized email template was sent to designated contact points for each mapped organization, requesting confirmation of identified initiatives and information on any additional activities not publicly documented.

### Data extraction and classification

For each initiative meeting the inclusion criteria, data were extracted into a structured format. Variables recorded included: initiative title, organizing body, country of provider (or “International” for supranational organizations), language of delivery, type of provider (Scientific Association/Society, School/University, International Organization, Other), type of initiative (training course, lecture series, seminar/webinar, conference session, summer/winter/spring school, degree programme, other), context (e.g., title of parent conference or master programme if applicable), delivery modality (online, in-person, hybrid), date of delivery, brief description, thematic content domains, source URL, and notes including documentation of retrieval difficulties.

Thematic content was classified according to domains derived from the ASPHER Core Curriculum for Public Health, which identifies “Digital Transformation in Public Health” as a cross-curricular competency area (18). Based on the competency descriptors outlined in the curriculum, the following eight domains were operationalised: (i) use of digital tools in public health practice; (ii) digital health literacy and digital determinants of health; (iii) leadership and management for digital transformation; (iv) health data collection and analysis; (v) health data governance; (vi) ethics and regulation of digital transformations; (vii) the infosphere and spread of information over digital networks; and (viii) the safe, ethical, and sustainable use of artificial intelligence in health. Domains were not mutually exclusive, and a single initiative could address multiple thematic areas.

Data extraction and thematic classification were carried out by the authors, with each member assigned to specific providers based on country and language competencies. Thematic classification was performed independently by two reviewers (A.G., C.B.), and any discrepancies were discussed and resolved by consensus.

### Data analysis

A descriptive data analysis was performed. Training initiatives were quantified and stratified by year, provider country, provider type, initiative type, delivery modality, language, and thematic content domain. Frequencies and percentages were calculated for categorical variables. For initiatives organized by multiple institutions, a single leading organization was identified and used for classification purposes. The leading organization was defined as the primary organizer responsible for the initiative, as reported in the original source material, and was used to assign provider type, provider country, and provider name in the analysis. Temporal trends were examined by plotting the number of initiatives per year across the study period. The distribution of thematic domains was analyzed overall and over time, as well as by training format to identify patterns in content coverage across different types of initiatives. A sensitivity analysis was conducted excluding conference sessions, to examine whether overall descriptive patterns were sensitive to the inclusion of short, event-based formats. All data analyses and visualization were performed using *R* statistical software (version 4.2.2).

## Results

### Training initiatives over time

**Figure 1:**
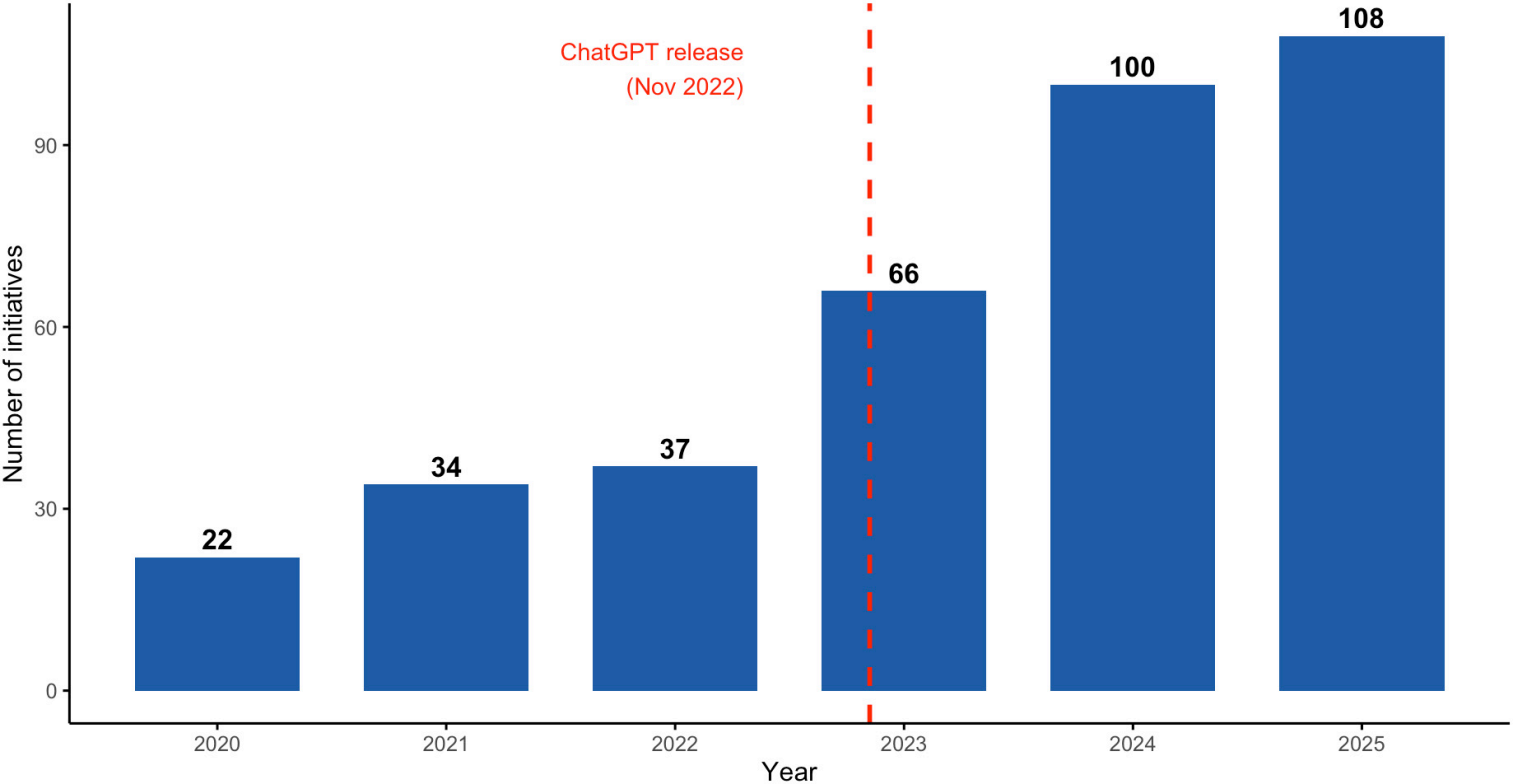
Digital Health and AI training initiatives for public health (2020–2025).

A total of 367 training initiatives focusing on DPH were identified across the study period from 2020 to 2025. As shown in Figure 1, the number of initiatives increased steadily over time. In 2020, 22 initiatives (6.0%) were identified, rising to 34 initiatives in 2021 (9.3%) and 37 initiatives in 2022 (10.1% each). A marked expansion was observed from 2023 onwards, with 66 initiatives in 2023 (18.0%), 100 in 2024 (27.2%), and a peak of 108 initiatives in 2025 (29.4%). Overall, more than half (*n* = 208, 56.7%) of all mapped initiatives were delivered in the final two years of the study period.

### Training formats and providers

The main characteristics of training initiatives are summarized in Table 1. Most initiatives were delivered through short and event-based formats. Conference-related activities, such as dedicated sessions within larger scientific meetings, represented the most common format (*n* = 200, 54.5%), followed by webinars and seminars (*n* = 82, 22.3%), and structured training courses (*n* = 67, 18.3%). Degree programmes were relatively rare (*n* = 12, 3.3%), while only a very limited number of initiatives consisted of lecture series (*n* = 3, 0.8%) or seasonal schools (*n* = 2, 0.5%).

**Table 1:** Integrated summary of digital health and artificial intelligence training initiatives for the public health workforce (Europe, 2020–2025).

**Category**	**Characteristic**	***n***	**%**
**Training format**	Conference session	200	54.5
	Seminar/Webinar	82	22.3
	Training course	67	18.3
	Degree programme	12	3.3
	Lecture series	3	0.8
	Seasonal school (summer/winter/spring)	2	0.5
	Other	1	0.3
**Delivery modality**	In-person	200	54.5
	Online	127	34.6
	Hybrid	40	10.9
**Provider type**	Scientific association/professional society	255	69.5
	Academic institution (school/university)	112	30.5
**Geographical origin of provider**	International organizations	119	32.4
	Germany	71	19.3
	Italy	58	15.8
	Spain	52	14.2
	France	34	9.3
	United Kingdom	33	9.0
**Language of delivery**	English	181	49.3
	Italian	58	15.8
	Spanish	52	14.2
	German	47	12.8
	French	23	6.3
	Bilingual (German, English)	3	0.8
	Bilingual (English, French)	3	0.8
**Thematic content domain** ^*^	Use of digital tools in public health	245	66.8
	Leadership and management for digital transformation	195	53.1
	Health data management and governance	152	41.4
	Ethics and regulation of digital transformation	152	41.4
	Safe, ethical, and sustainable use of AI in health	131	35.7
	Health data collection and analysis	129	35.1
	Digital health literacy and digital determinants of health	85	23.2
	Infosphere and spread of information over digital networks	9	2.5
**Top training providers**	European Public Health Association (EUPHA)	41	11.2
	WHO Regional Office for Europe	36	9.8
	European Health Management Association (EHMA)	24	6.5
	Italian Society of Hygiene, Preventive Medicine and Public health (SItI)	19	5.2
	Asociación Juristas de la Salud (AJS)	16	4.4
	German Society for Social Medicine and Prevention (DGSMP)	14	3.8
	EHESP School of Public Health	14	3.8
	National Association of Hospital Directors (ANMDO)	12	3.3
	University of Bristol	11	3.0
	Federal Association of Physicians of German Public Health Departments (BVÖGD) (BVÖGD)	11	3.0

^*^Thematic content domains are not mutually exclusive; a single training initiative may address multiple domains.

Regarding the delivery modality, in-person formats accounted for the largest share of initiatives (*n* = 200, 54.5%), while over one third were delivered online (*n* = 127, 34.6%) and a smaller proportion adopted a hybrid format (*n* = 40, 10.9%).

Most initiatives were delivered by scientific associations or professional societies (*n* = 255, 69.5%), while academic institutions, including PH schools and universities, accounted for 30.5% of initiatives (*n* = 112). Regarding the geographical distribution of providers, initiatives were most frequently delivered by international organizations (*n* = 119, 32.4%). Among country-specific providers, Germany accounted for almost 20% of initiatives (*n* = 71, 19.3%), followed by Italy (*n* = 58, 15.8%), Spain (*n* = 52, 14.2%), France (*n* = 34, 9.3%), and the United Kingdom (*n* = 33, 9.0%).

During the direct contact phase, emails were sent to 226 providers across all countries, with an overall response rate of 8.4% (*n* = 19). A total of 26 initiatives (7.1%) included in the final dataset were identified through institutional contact. Response rates varied by country: Italy had the highest response rate (26.7%, 12 of 45), followed by the United Kingdom (7.3%, 3 of 41), France (4.3%, 2 of 47), Spain (4.3%, 2 of 46), and Germany (0%, 0 of 40). Seven emails were sent to international organizations, with no responses received. The proportion of initiatives ultimately added through direct institutional contact also varied considerably: in France, 41.2% (14 of 34) were identified following email contact, compared to 13.5% in Spain (7 of 52), 6.9% in Italy (4 of 58), 3.0% in the United Kingdom (1 of 33), and 0% in Germany and international organizations.

Analysis of training providers revealed a concentration of initiatives among a limited number of organizations (Table 1). The European Public Health Association (EUPHA) was the most frequent provider (*n* = 41, 11.2%), followed by the WHO Regional Office for Europe (*n* = 36, 9.8%) and the European Health Management Association (EHMA) (*n* = 24, 6.5%). Other recurrent providers included the Italian Society of Hygiene, Preventive Medicine and Public Health (SItI) (*n* = 19, 5.2%), the Spanish Society “*Asociación Juristas de la Salud”* (AJS) (*n* = 16, 4.4%), the German Society for Social Medicine and Prevention (DGSMP) (*n* = 14, 3.8%), the *Ecole des hautes études en santé publique* (EHESP) French School of Public Health (*n* = 14, 3.8%), the National Association of Hospital Directors (ANMDO) (*n* = 12, 3.3%), the Federal Association of Physicians of German Public Health Departments (BVÖGD) (*n* = 11, 3.0%), and the University of Bristol (*n* = 11, 3.0%).

Nearly half of initiatives were delivered in English (*n* = 181, 49.3%), followed by Italian (*n* = 58, 15.8%), Spanish (*n* = 52, 14.2%), German (*n* = 47, 12.8%), and French (*n* = 23, 6.3%), with a small number of bilingual initiatives delivered in both German and English (*n* = 3, 0.8%,), and English and French (*n* = 3, 0.8%).

### Training content domains

The distribution of training content domains across initiatives is shown in Table 1. Overall, the most frequently addressed content domain was the use of digital tools (*n* = 245, 66.8%), followed by leadership and management skills applied to digital transformation in health (*n* = 195, 53.1%). Health data management and governance (*n* = 152, 41.4%) and ethical and regulatory aspects of digital transformation (*n* = 152, 41.4%) were also commonly represented. The safe, ethical, and sustainable use of AI in health was addressed in 35.7% initiatives (*n* = 131), and health data collection and analysis in 35.1% initiatives (*n* = 129). The DH literacy and digital determinants of health domain was moderately represented (*n* = 85, 23.2%), while topics related to the infosphere and the spread of information over digital networks were rarely addressed (*n* = 9, 2.5%).

**Figure 2:**
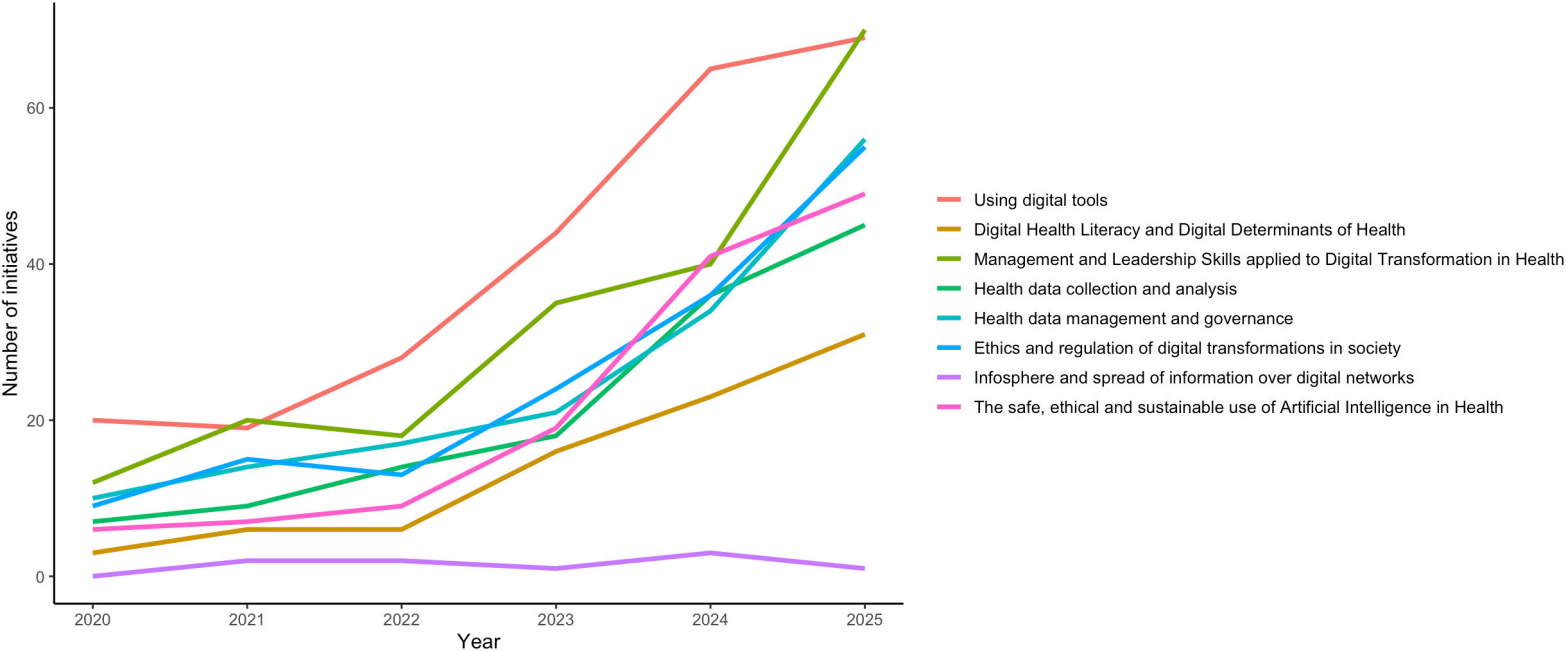
Distribution of thematic content domains (2020–2025)

As shown in Figure 2, most content domains increased over time, with a more pronounced rise from 2023 onwards. This pattern was particularly evident in leadership and management skills, the ethical and regulatory aspects of digital transformation, and AI-related content. In contrast, the number of initiatives addressing the infosphere and the spread of information over digital networks remained consistently low throughout the study period.

**Figure 3:**
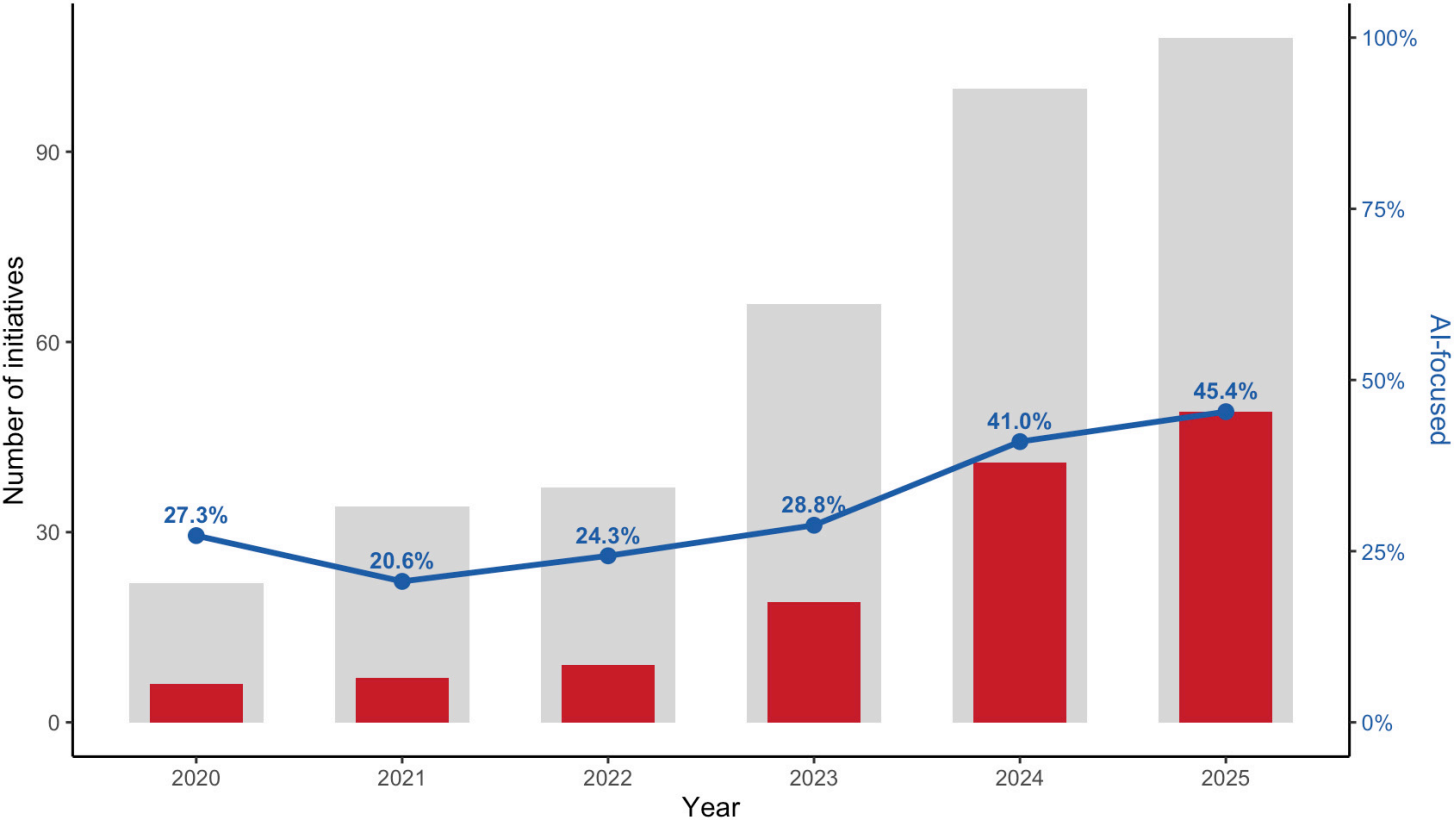
Trend in training initiatives for safe, ethical, and sustainable AI in health (2020–2025).

**Figure 4:**
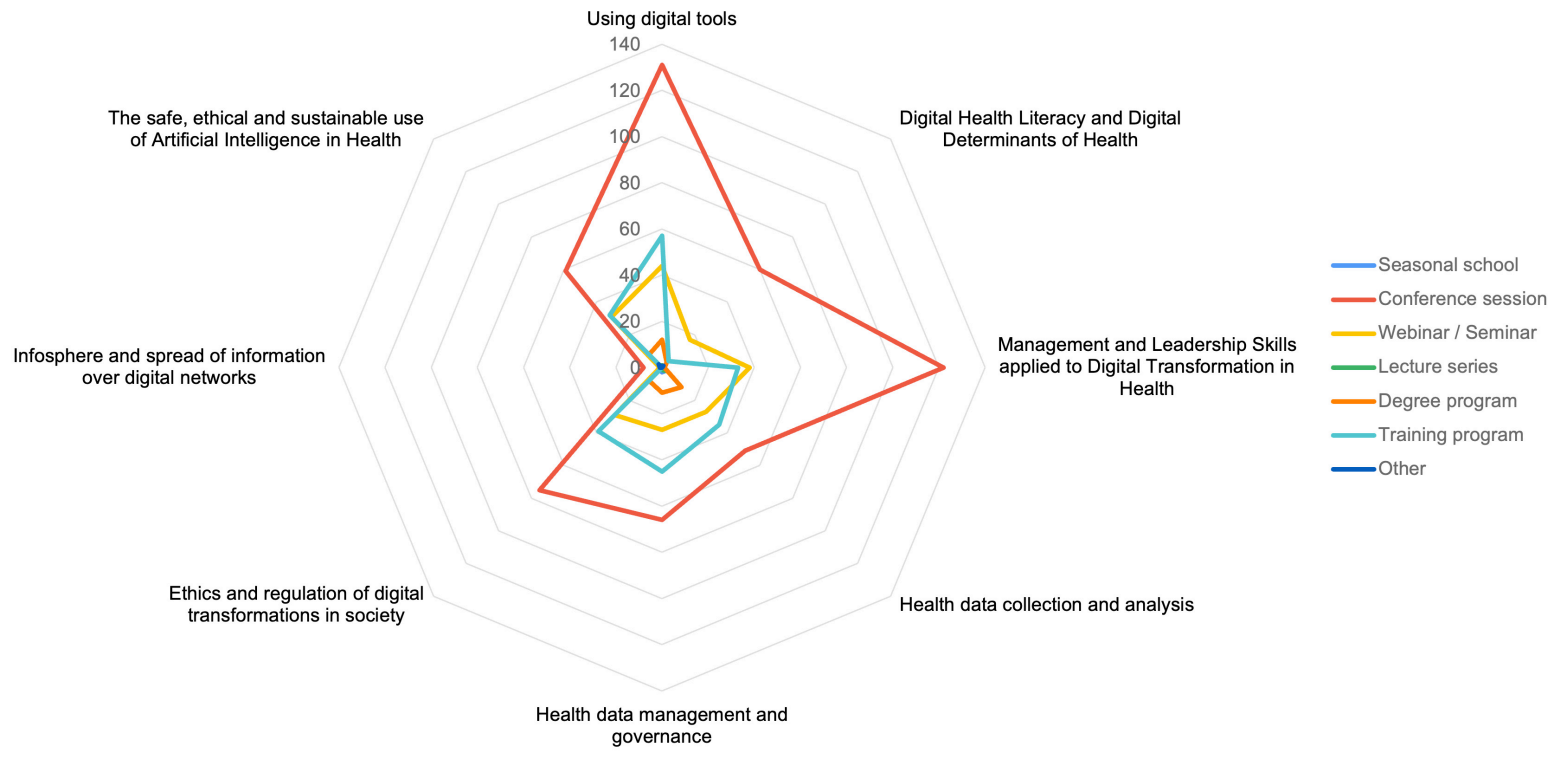
Distribution of thematic content domains by training format (2020–2025).

A closer examination of AI-focused initiatives is presented in Figure 3. The proportion of training programmes addressing the safe, ethical and sustainable use of AI in health remained relatively stable between 2020 and 2022, ranging from 27.3% in 2020 (6 of 22 initiatives) to 20.6% in 2021 (7 of 34) and 24.3% in 2022 (9 of 37). From 2023 onwards, a marked increase was observed, with AI-focused initiatives representing 28.8% in 2023 (19 of 66), 41.0% in 2024 (41 of 100), and 45.4% in 2025 (49 of 108). Figure 4 shows differences in the distribution of content domains by training format. Conference-related activities accounted for the largest proportion of initiatives across all content domains, particularly in the use of digital tools and leadership and management skills. Webinars and seminars exhibited a similar, albeit more selective, distribution of domains. Structured training programmes more frequently combined domains related to data governance, data collection and analysis, and ethical and regulatory aspects within the same initiative. Degree programmes and lecture series contributed only marginally to the overall distribution of domains.

In a sensitivity analysis excluding conference sessions, the overall distribution of content domains remained largely consistent. The most frequently addressed domain was the use of digital tools (68.3%), followed by health data management and governance (51.5%), health data collection and analysis (46.7%), the ethical and regulatory aspects of digital transformation (46.1%), and AI-related content (43.1%). Topics related to digital health literacy, the digital determinants of health, and the infosphere and spread of information remained marginal, accounting for 15% and 0.6% of initiatives, respectively.

## Discussion

This mapping review provides an overview of DPH training initiatives in five European countries and major international organizations between 2020 and 2025. Our findings suggest a rapidly expanding training offer, with a marked increase in initiatives after 2022. However, this growth is accompanied by a clear predominance of short, event-based formats with academic institutions contributing a smaller share of training initiatives compared to scientific associations and professional societies. Training provision is primarily driven by scientific associations and international organizations, whereas structured educational pathways within PH schools and universities remain relatively uncommon. Additionally, training content is distributed unevenly across thematic domains, frequently emphasizing digital tools and leadership for digital transformation while comparatively neglecting areas such as the infosphere and the spread of information over digital networks. Overall, despite the growing volume of training opportunities, the current educational landscape may not yet fully support the development of the sustained, comprehensive competencies required for effective PH practice in modern DH systems.

### A fragmented training landscape

The most salient finding of this review is the fragmented nature of DPH training for PH professionals. Over three-quarters of identified initiatives consisted of conference sessions or webinars, formats that, while valuable for awareness-raising and knowledge dissemination, are poorly suited for building the sustained competencies required for effective DH practice. Structured educational pathways, including courses and degree programmes, represented a minority of the total, with formal academic programmes being particularly rare.

This pattern is consistent with findings from other reviews. Charow et al., in their scoping review of AI education programmes for health professionals, identified only 13 unique programmes globally, ranging from 1-h journal clubs to multi-year certificates, and concluded that existing opportunities remain limited (22). Kaihlanen et al., mapping digital skills continuing education across 25 EU Member States, found similarly fragmented organization both within and between countries, with half of the surveyed countries lacking any national coordination or designated authority for digital competency training (24). Our findings confirm that this fragmentation extends specifically to the PH workforce. The implications warrant consideration. Conference sessions and webinars typically deliver information to relatively passive recipients rather than providing the sustained engagement necessary to develop practical skills in data analytics, AI tool evaluation, or digital epidemiology. The current landscape offers numerous opportunities to learn about DH concepts, but more limited opportunities to develop operational competence.

### The institutional gap between academia and professional training

A second major finding concerns the disproportionate role of scientific and professional associations compared to academic institutions. This imbalance suggests that a substantial share of training opportunities is embedded within professional or conference-related contexts rather than within formal academic curricula. While associations have demonstrated considerable agility in responding to emerging training needs, particularly visible in the sharp increase in offerings after 2022, this pattern raises questions about the sustainability and depth of workforce preparation.

Academic institutions, and specifically schools of PH, bear primary responsibility for foundational professional training. Yet our data suggest they have been slower to integrate DPH content into their curricula. This observation aligns with Maaß et al.'s analysis of German PH programmes, which found that only 16 of 79 programmes offered even a single module on DPH, and that existing modules focused narrowly on technical aspects rather than on integrated competencies (25). Hrzic et al. (7) reached similar conclusions, noting that digital skills are not yet systematically incorporated into PH curricula and recommending integration throughout the curriculum rather than isolation in standalone courses.

The possible consequences of this institutional gap merit attention. First, early-career professionals may enter the workforce without foundational digital competencies, creating a remedial training burden that falls disproportionately on employers and professional associations. Second, the current system may privilege those with resources, time, and institutional support to pursue voluntary continuing education, potentially widening inequities in workforce capability. Williams et al., in their European Observatory policy brief, caution against allowing digital skills education to remain solely a burden for employers or individual professionals and call for earmarked funding and the legislative embedding of digital competencies in core curricula (26).

### The inflection point

The temporal distribution of initiatives reveals a marked acceleration after 2022, with more than half of all mapped initiatives delivered in the final two years of the study period alone. The COVID-19 pandemic likely accelerated awareness of DH infrastructure needs from 2020 onwards, but the post-2022 surge coincides with the public release of large language models, particularly ChatGPT, in November 2022.

This pattern suggests that generative AI may have functioned as a catalyst for broader engagement with DPH training, drawing attention to digital competencies more generally. However, it also raises questions about depth vs. breadth: the already discussed predominance of short formats among post-2022 offerings suggests that at least some training activity may represent reactive programming rather than systematic competency development.

### Thematic imbalances in training content

The distribution of initiatives across thematic domains reveals notable imbalances. While “Use of Digital Tools” was included in over half of all initiatives, training related to the infosphere and misinformation, accounted for a minority of the total.

This gap is concerning, given the centrality of infodemics to contemporary PH practice. The COVID-19 pandemic demonstrated that health misinformation can directly undermine vaccination campaigns, erode public trust, and contribute to adverse health outcomes. DH competence for PH professionals should arguably include the ability to monitor, analyse, and counter health misinformation in digital environments. Yet our mapping suggests this remains a relatively neglected domain in both formal and informal training.

The deficit may partly reflect disciplinary boundaries: infodemic management sits at the intersection of communication science, behavioral science, and digital epidemiology, and may fall between traditional PH training categories. It is also possible that our search strategy, oriented primarily toward DH and AI terminology, may have underidentified training initiatives framed primarily in communication or media literacy terms. Regardless of the explanation, addressing this gap should be a priority for curriculum developers and training providers.

### Country variation and data accessibility

The distribution of initiatives across countries showed notable variation, with Germany and Italy showing higher representation than France and the United Kingdom. These differences may reflect genuine variation in training activity, differential institutional engagement with DPH topics, or variation in how training opportunities are documented and made publicly accessible. Our experience with France illustrates this complexity: a substantial portion of French initiatives was identified only through direct institutional contact rather than through systematic online searching, a proportion that is decisively higher than in other countries. This suggests that differences in public documentation practices across countries, in the absence of standardized reporting mechanisms, may influence the visibility of training activities independently of their actual volume. For countries and institutions, this observation raises questions about whether training offerings are reaching professionals beyond immediate institutional networks.

This observation aligns with findings from other European mapping studies. Kaihlanen et al., mapping continuing education in digital skills across 25 EU Member States, reported that half of the countries surveyed lacked national coordination or a systematic approach to organizing such training, with fragmented provision across diverse stakeholders and limited accreditation systems (24). The low public accessibility of training information observed in our study may partly reflect this fragmented organization. Moreover, it is likely that a proportion of digital skills development occurs through informal, workplace-based learning that is rarely documented or credited, yet may play a substantial role in building practical competencies (27). Future research should examine the extent and effectiveness of such informal learning pathways alongside formal training provision.

For policymakers and training providers, these findings highlight the importance of improving the discoverability of training opportunities. Centralized registries, standardized reporting, and coordinated communication strategies could help ensure that available training reaches the professionals who need it.

### Implications for policy and curriculum reform

Overall, these findings support several considerations for strengthening DPH training.

First, the current training landscape highlights the need for academic institutions, particularly schools of PH, to accelerate the integration of digital competencies into their core curricula. While the ASPHER Core Curriculum provides a framework for digital transformation in PH education (18), implementation will require accreditation bodies, programme directors, and faculty to prioritize digital competencies alongside more established areas of PH training. However, translating frameworks into practice will also depend on the development of shared standards. Although various competency frameworks have been proposed (7, 18–21), the field has not yet reached consensus on core digital public health competencies or on validated approaches to assess them (19, 28, 29). Furthermore, future research should examine the pedagogical effectiveness of different training formats and their capacity to build sustained competencies, questions that remain largely unanswered in this emerging field. Advancing this evidence base should be a priority alongside curricular reform efforts. In this context, some authors have proposed more formalized pathways, such as Car and Topol's recent call for a standardized Master of DH degree modeled on the MPH (30), although the applicability of such models may vary across different national and educational settings.

Second, the balance between associations and academic institutions may need reconsideration. Professional associations have demonstrated valuable responsiveness to emerging needs, but they cannot substitute for foundational academic training. A more sustainable model would position academic institutions as providers of baseline competencies, while professional associations support continuing education, updating competencies as technologies evolve.

Third, the content gaps identified in DPH training, particularly regarding the infosphere and misinformation, warrant targeted attention. This may involve developing dedicated training resources, integrating infodemic competencies into existing DPH curricula, or establishing partnerships with communication and behavioral science programmes.

Finally, sustainable workforce development will likely require policy action at national and European levels. As Williams et al. argue, this includes developing national strategies, legislative frameworks embedding digital competencies into training requirements, and dedicated funding (26). Without systemic investment, DH training risks remaining fragmented, unevenly distributed, and insufficient to meet the demands of an increasingly DH system.

### Strengths and limitations

This study has several limitations. As a mapping review focused on gray literature, it prioritized breadth over completeness, and some training initiatives are likely to have been missed. As the review relied on publicly accessible sources, training activities not present online may be underrepresented, a point exemplified by the identification of additional French initiatives through direct institutional contact. Language restrictions, aligned with the research team's competencies, may also have limited the inclusion of initiatives delivered in other languages. Additionally, the scope and methodological approach of this study also influenced the type of information that could be obtained. As the study focused on real-world provision, it was anticipated that many training providers would not report on aspects such as teaching methods, group size, and learning outcomes. Similarly, it was not possible to evaluate training quality or effectiveness. Finally, this study did not assess whether training initiatives effectively build the competencies required for digital public health practice. Available evidence suggests that competency measurement in this field relies predominantly on self-report approaches, with limited correspondence to objectively demonstrated proficiency (28, 31). These gaps represent broader challenges for research in this emerging field, and addressing them will require dedicated methodological work alongside continued mapping of training provision.

At the same time, this study has several important strengths. It presents a systematic overview of DPH training initiatives for the PH workforce in several European countries and international organizations. The emphasis on real-world training delivery offers a grounded view of what is currently available and highlights areas where further development may be needed. The use of an established and structured framework supported a transparent and coherent thematic classification, while the multilingual, multi-country search strategy, combined with the inclusion of gray literature and direct institutional contact, helped to identify training activities often overlooked in conventional reviews.

## Conclusion

This mapping review documents a rapidly expanding but structurally fragmented landscape of DPHtraining in Europe. The surge in activity since 2022 demonstrates growing recognition of digital competencies as essential to practice. Yet the predominance of short formats, the comparatively smaller contribution of academic institutions relative to event-based provision, and uneven coverage of critical domains such as infodemic management suggest that current efforts remain insufficient.

Turning this growing interest into sustainable workforce capacity will require systematic curricular integration, coordination between academic institutions and professional associations, attention to underrepresented content areas, and policy frameworks that support training at the system level. As the PH workforce of the coming decades will operate in environments shaped by AI-assisted decision-making, real-time data systems, and persistent digital misinformation, workforce training extends beyond curricular considerations and directly affects PH system resilience.

## Data Availability

**T**he original contributions presented in the study are included in the article/Supplementary material, further inquiries can be directed to the corresponding author.
