# Supplementary material for: Mapping digital public health training: are we preparing the European workforce?
Source: Front Public Health. 2026 Feb 11;14:1778953. doi: 10.3389/fpubh.2026.1778953 (PMC12932469; doi:10.3389/fpubh.2026.1778953)
Supplement: Supplementary file 1 [file Data_Sheet_1.docx]

**Supplementary Table S1.** List of public health institutions considered as providers of training initiatives in digital health and artificial intelligence for public health

| International  institutions | - Directorate-General for Health and Food Safety of the European Commission (DG SANTE) - EuroHealthNet - European Centre for Disease Prevention and Control (ECDC) - European Health Management Association (EHMA) - European Observatory on Health Systems and Policies - European Public Health Alliance (EPHA) - European Public Health Association (EUPHA) - WHO Regional Office for Europe (WHO Europe) |
| --- | --- |
| United Kingdom | - Association of Directors of Public Health (ADPH) - East Midlands Deanery, specialty training in public health - East of England Deanery, specialty training in public health Kent, Surrey and Sussex Deanery, KSS School of Public Health - London Deanery, specialty training in public health - North East Deanery, specialty training in public health - North West Deanery, specialty training in public health - Northern Ireland Deanery, specialty training in public health - Scotland Deanery, specialty training in public health - South West Deanery, specialty training in public health - Thames Valley Deanery, specialty training in public health - Wales Deanery, specialty training in public health - Wessex Deanery, specialty training in public health - West Midlands Deanery, specialty training in public health - Yorkshire and the Humber Deanery, specialty training in public health - Imperial College London, Master of Public Health (MPH) - London School of Hygiene and Tropical Medicine, MSc in Public Health - Middlesex University, MSc in Public Health - NHS Confederation - Society for Social Medicine and Population Health - The University of Edinburgh, Master of Public Health (MPH) - UK Faculty of Public Health - University College London (UCL), Master of Public Health (MPH) - University of Cambridge, Cambridge Institute of Public Health - University of Chester, Master of Public Health (MPH) - University of Edinburgh, Usher Institute - University of Liverpool, Department of Public Health and Policy - University of Nottingham, School of Medicine - University of Sheffield, Master of Public Health (MPH) - University of York, Master of Public Health (MPH) |
| France | - Aix-Marseille Université, master in public health - AP-HM (Marseille) / Aix-Marseille Université, postgraduate medical specialty training in public health - AP-HP (Paris) / Sorbonne Université / Université Paris Cité / Université Paris-Saclay, postgraduate medical specialty training in public health - CHRU de Besançon / Université de Franche-Comté, postgraduate medical specialty training in public health - CHRU de Brest / Université de Bretagne Occidentale, postgraduate medical specialty training in public health - CHRU de Lille / Université de Lille, postgraduate medical specialty training in public health - CHRU de Nancy / Université de Lorraine, postgraduate medical specialty training in public health - CHRU de Tours / Université de Tours, postgraduate medical specialty training in public health - CHU Amiens-Picardie / Université de Picardie Jules Verne, postgraduate medical specialty training in public health - CHU d’Angers / Université d’Angers, postgraduate medical specialty training in public health - CHU de Bordeaux / Université de Bordeaux (ISPED), postgraduate medical specialty training in public health - CHU de Caen Normandie / Université de Caen Normandie, postgraduate medical specialty training in public health - CHU de Clermont-Ferrand / Université Clermont Auvergne, postgraduate medical specialty training in public health - CHU de Guadeloupe / CHU de Martinique / Université des Antilles, postgraduate medical specialty training in public health - CHU de La Réunion / Université de La Réunion, postgraduate medical specialty training in public health - CHU de Limoges / Université de Limoges, postgraduate medical specialty training in public health - CHU de Montpellier / CHU de Nîmes / Université de Montpellier, postgraduate medical specialty training in public health - CHU de Nantes / Nantes Université, postgraduate medical specialty training in public health - CHU de Nice / Université Côte d’Azur, postgraduate medical specialty training in public health - CHU de Poitiers / Université de Poitiers, postgraduate medical specialty training in public health - CHU de Reims / Université de Reims Champagne-Ardenne, postgraduate medical specialty training in public health - CHU de Rennes / Université de Rennes (EHESP), postgraduate medical specialty training in public health - CHU de Rouen / Université de Rouen Normandie, postgraduate medical specialty training in public health - CHU de Saint-Étienne / Université Jean Monnet, postgraduate medical specialty training in public health - CHU de Toulouse / Université Toulouse III Paul Sabatier, postgraduate medical specialty training in public health - CHU Dijon Bourgogne / Université de Bourgogne, postgraduate medical specialty training in public health - CHU Grenoble Alpes / Université Grenoble Alpes, postgraduate medical specialty training in public health - Conférence des Directeurs Généraux de CHU - École des hautes études en santé publique (EHESP), school of public health - Hôpitaux Universitaires de Strasbourg / Université de Strasbourg, postgraduate medical specialty training in public health - Hospices Civils de Lyon (HCL) / Université Claude Bernard Lyon 1, postgraduate medical specialty training in public health - Société Française de Santé Publique - Sorbonne Université, master in public health - Université Claude Bernard Lyon 1, master in public health - Université de Bordeaux (ISPED), master in public health - Université de Caen Normandie, master in public health - Université de Limoges, master in public health - Université de Lorraine, master in public health - Université de Rennes, master in public health - Université de Strasbourg, master in public health - Université de Tours, master in public health - Université Paris Cité, master in public health - Université Paris-Est Créteil, master in public health - Université Toulouse III, master in public health - University of Bordeaux, ISPED School of Public Health - University of Lorraine, School of Public Health |
| Germany | - Academy of Public Health - Alice Salomon Hochschule (ASH) Berlin, MA in Public Health - Bundesverband der Ärztinnen und Ärzte des Öffentlichen Gesundheitsdienstes (BVÖGD) - Chemnitz University of Technology, MSc in Public Health - Deggendorf Institute of Technology, MSc in Global Public Health - Dresden University of Technology, Department of Health Sciences - Dresden University of Technology, Master of Public Health (MPH) - Fulda University of Applied Sciences, Department for Nursing and Health Sciences - Fulda University of Applied Sciences, MSc in Public Health - German Public Health Association (DGPH) - German Society for Social Medicine and Prevention (DGSMP) - Hamburg University of Applied Sciences, Master of Public Health - Hannover Medical School, MSc in Public Health - Heidelberg University Hospital, Institute of Public Health - Heinrich Heine University Düsseldorf, MSc in Public Health - IU Internationale Hochschule, Master of Public Health (MSc) - Jade University of Applied Sciences (Jade Hochschule), MSc in Public Health - Leuphana University of Lüneburg, Centre for Applied Health Sciences - Leuphana University of Lüneburg, Master of Public Health (MPH) - Ludwig Maximilian University of Munich, MSc in Public Health - Ludwig-Maximilians University Munich, Pettenkofer School of Public Health - Medical Faculty, Heinrich Heine University Düsseldorf, Düsseldorf School of Public Health - Charité, Universitätsmedizin Berlin, Berlin School of Public Health - NRW Centre for Health (LZG.NRW) - Protestant University of Applied Sciences, Institute of Applied Research - School of Public Health, Universität Bielefeld, MSc in Public Health - Technische Hochschule Mittelhessen (THM), MSc in Public Health - Technische Universität Berlin, MSc in Public Health - Universität Bremen, Public Health (Health Promotion and Prevention), MA - Universität Bremen, Public Health (Health Sciences), BA - University of Bielefeld, School of Public Health - University of Europe for Applied Sciences (UE), MSc in International Public Health Management - University of Siegen, MSc in Public Health - Verband der Krankenhausdirektoren Deutschlands (VKD) |
| Italy | - Associazione Nazionale Medici delle Direzioni Ospedaliere (ANMDO) - CEFPAS, Caltanissetta, Centre for Training and Research in Public Health - Confederazione Associazioni Regionali di Distretto (CARD) - Humanitas University, postgraduate medical specialty training in public health - Italian Society of Hygiene, Preventive Medicine and Public Health (SItI) - Università Cattolica del Sacro Cuore, postgraduate medical specialty training in public health - Università degli Studi “G. D’Annunzio” Chieti, postgraduate medical specialty training in public health - Università degli Studi “Magna Græcia” di Catanzaro, postgraduate medical specialty training in public health - Università degli Studi del Molise, postgraduate medical specialty training in public health - Università degli Studi dell’Aquila, postgraduate medical specialty training in public health - Università degli Studi dell’Insubria, postgraduate medical specialty training in public health - Università degli Studi della Campania “Luigi Vanvitelli”, postgraduate medical specialty training in public health - Università degli Studi di Bari Aldo Moro, postgraduate medical specialty training in public health - Università degli Studi di Brescia, postgraduate medical specialty training in public health - Università degli Studi di Cagliari, postgraduate medical specialty training in public health - Università degli Studi di Enna “Kore”, postgraduate medical specialty training in public health - Università degli Studi di Ferrara, postgraduate medical specialty training in public health - Università degli Studi di Firenze, postgraduate medical specialty training in public health - Università degli Studi di Messina, postgraduate medical specialty training in public health - Università degli Studi di Milano, postgraduate medical specialty training in public health - Università degli Studi di Milano-Bicocca, postgraduate medical specialty training in public health - Università degli Studi di Modena e Reggio Emilia, postgraduate medical specialty training in public health - Università degli Studi di Napoli Federico II, postgraduate medical specialty training in public health - Università degli Studi di Palermo, postgraduate medical specialty training in public health - Università degli Studi di Perugia, postgraduate medical specialty training in public health - Università degli Studi di Salerno, postgraduate medical specialty training in public health - Università degli Studi di Sassari, postgraduate medical specialty training in public health - Università degli Studi di Trieste, postgraduate medical specialty training in public health - Università degli Studi di Udine, postgraduate medical specialty training in public health - Università degli Studi di Verona, postgraduate medical specialty training in public health - Università del Piemonte Orientale, postgraduate medical specialty training in public health - Università di Bologna, postgraduate medical specialty training in public health - Università di Catania, postgraduate medical specialty training in public health - Università di Foggia, postgraduate medical specialty training in public health - Università di Genova, postgraduate medical specialty training in public health - Università di Padova, postgraduate medical specialty training in public health - Università di Parma, postgraduate medical specialty training in public health - Università di Pavia, postgraduate medical specialty training in public health - Università di Pisa, postgraduate medical specialty training in public health - Università di Roma Tor Vergata, postgraduate medical specialty training in public health - Sapienza Università di Roma, postgraduate medical specialty training in public health - Università di Siena, postgraduate medical specialty training in public health - Università di Torino, postgraduate medical specialty training in public health - Università Politecnica delle Marche, postgraduate medical specialty training in public health - Università Vita-Salute San Raffaele, postgraduate medical specialty training in public health |
| Spain | - Hospital del Mar, Barcelona, postgraduate medical specialty training in public health - Biosanitary Research Institute of Granada, Andalusian School of Public Health - Escola Valenciana d’Estudis de la Salut (EVES) - Escuela Andaluza de Salud Pública (EASP) - Escuela Nacional de Sanidad (ENS) - Pompeu Fabra University / Autonomous University of Barcelona, Master of Public Health (MPH) - Public University of Navarra, Faculty of Health Sciences - Sociedad Española de Directivos de la Salud (SEDISA) - Sociedad Española de Medicina Preventiva, Salud Pública y Gestión Sanitaria (SEMPSPGS) - Sociedad Española de Medicina Preventiva, Salud Pública e Higiene - Sociedad Española de Salud Pública y Administración Sanitaria (SESPAS) - UD MPYSP Andalucía, postgraduate medical specialty training in public health - UD MPYSP de Aragón, postgraduate medical specialty training in public health - UD MPYSP de Canarias, postgraduate medical specialty training in public health - UD MPYSP de Cantabria, postgraduate medical specialty training in public health - UD MPYSP de Castilla-La Mancha, postgraduate medical specialty training in public health - UD MPYSP de Castilla y León, postgraduate medical specialty training in public health - UD MPYSP de Euskadi, postgraduate medical specialty training in public health - UD MPYSP de Galicia, postgraduate medical specialty training in public health - UD MPYSP de la Región de Murcia, postgraduate medical specialty training in public health - UD MPYSP de Navarra, postgraduate medical specialty training in public health - UD MPYSP del Principado de Asturias, postgraduate medical specialty training in public health - Universidad de Alicante, master in public health - Universidad de Murcia, master in public health - Universidade de Santiago de Compostela, Master of Public Health (MPH) - Universitat de València, master in public health and health management - University of Granada, Europubhealth+, European Public Health Master |

**Supplementary Table S2.** Search strings used for web-based searches

| English | ("digital health" OR "eHealth" OR "health informatics" OR "telehealth" OR "artificial intelligence" OR "AI" OR "machine learning" OR "health data science")  AND ("public health" OR "epidemiology" OR "health promotion" OR "preventive medicine" OR "health management" OR "global health")  AND (course OR training OR workshop OR webinar OR seminar OR "summer school" OR "continuing education" OR "Continuing Medical Education" OR masterclass)  AND (2020 OR 2021 OR 2022 OR 2023 OR 2024 OR 2025) |
| --- | --- |
| French | ("santé numérique" OR "e-santé" OR "informatique de santé" OR "télésanté" OR "intelligence artificielle" OR "IA" OR "apprentissage automatique" OR "science des données de santé")  AND ("santé publique" OR "épidémiologie" OR "promotion de la santé" OR "médecine préventive" OR "gestion de la santé" OR "santé mondiale")  AND (cours OR formation OR atelier OR webinaire OR séminaire OR "université d'été" OR "formation continue" OR "Formation Médicale Continue" OR masterclass)  AND (2020 OR 2021 OR 2022 OR 2023 OR 2024 OR 2025) |
| German | ("digitale Gesundheit" OR "e-Health" OR "Gesundheitsinformatik" OR "Telemedizin" OR "künstliche Intelligenz" OR "KI" OR "maschinelles Lernen" OR "Gesundheitsdatenwissenschaft")  AND ("öffentliche Gesundheit" OR "Epidemiologie" OR "Gesundheitsförderung" OR "Präventivmedizin" OR "Gesundheitsmanagement" OR "globale Gesundheit")  AND (Kurs OR Schulung OR Fortbildung OR Workshop OR Webinar OR Seminar OR "Summer School" OR Weiterbildung)  AND (2020 OR 2021 OR 2022 OR 2023 OR 2024 OR 2025) |
| Italian | ("salute digitale" OR "sanità digitale" OR "informatica sanitaria" OR "telemedicina" OR "intelligenza artificiale" OR "IA" OR "apprendimento automatico" OR "scienza dei dati sanitari")  AND ("sanità pubblica" OR "epidemiologia" OR "promozione della salute" OR "medicina preventiva" OR "management sanitario" OR "salute globale")  AND (corso OR formazione OR workshop OR webinar OR seminario OR "summer school" OR "educazione continua" OR masterclass)  AND (2020 OR 2021 OR 2022 OR 2023 OR 2024 OR 2025) |
| Spanish | ("salud digital" OR "eSalud" OR "informática de la salud" OR "telesalud" OR "inteligencia artificial" OR "IA" OR "aprendizaje automático" OR "ciencia de datos en salud")  AND ("salud pública" OR "epidemiología" OR "promoción de la salud" OR "medicina preventiva" OR "gestión sanitaria" OR "salud global")  AND (curso OR formación OR taller OR webinar OR seminario OR "escuela de verano" OR "educación continua" OR "clase magistral")  AND (2020 OR 2021 OR 2022 OR 2023 OR 2024 OR 2025) |

**Supplementary Table S3.** Full dataset of mapped training initiatives

| **Original titl the training initiative** | **Provider** | **Country** | **Language** | **Category of provider** | **Initiative format** | **Context** | **Delivery** | **Year** | **Using digital tools** | **Digital Health Literacy and Digital Determinants of Health** | **Management and Leadership Skills applied to Digital Transformation in Health** | **Health data collection and analysis** | **Health data management and governance** | **Ethics and regulation of digital transformations in society** | **Infosphere and spread of information over digital networks** | **The safe, ethical and sustainable use of Artificial Intelligence in Health** | **Description** | **Source** | **Added following email communication** |
| --- | --- | --- | --- | --- | --- | --- | --- | --- | --- | --- | --- | --- | --- | --- | --- | --- | --- | --- | --- |
| Plenary 4: The Digital Information Revolution | European Public Health Association (EUPHA); World Federation of Public Health Associations (WFPHA) | International organisations | English | Scientific Association/Society | Conference session | 16th World Conference of Public Health 2020 | Online | 2020 | N | Y | Y | N | N | Y | N | N | Plenary session on the digital information revolution in public health. | <https://academic.oup.com/eurpub/issue/30/Supplement_5?login=false> | N |
| Pre-conference: Privacy by default, security by design. Effects of GDPR on public health research | European Public Health Association (EUPHA); World Federation of Public Health Associations (WFPHA) | International organisations | English | Scientific Association/Society | Conference session | 16th World Conference of Public Health 2020 | Online | 2020 | N | N | N | Y | Y | Y | N | N | Pre-conference jointly organised with EUPHA-DH, EUPHA-PHMR, EUPHA-ETH, EUPHA-HSR and EUPHA-LAW on privacy and data security in public health research under GDPR. | <https://eupha.org/repository/sections/dh/EUPHA-DH%20Annual%20report%202020.pdf> | N |
| Round table: Harnessing the digital transformation of disease prevention: a focus on m-health and genomics | European Public Health Association (EUPHA); World Federation of Public Health Associations (WFPHA) | International organisations | English | Scientific Association/Society | Conference session | 16th World Conference of Public Health 2020 | Online | 2020 | Y | N | Y | N | Y | N | N | N | Joint roundtable with EUPHA-DH, EUPHA-EPI, EUPHA-PHG and EUPHA-HP on digital transformation in disease prevention, focusing on m-health and genomics. | <https://academic.oup.com/eurpub/article/30/Supplement_5/ckaa165.967/5915814> | N |
| Workshop: Assessment, regulation and use of apps for health management: where we are and where we are going? | European Public Health Association (EUPHA); World Federation of Public Health Associations (WFPHA) | International organisations | English | Scientific Association/Society | Conference session | 16th World Conference of Public Health 2020 | Online | 2020 | Y | N | N | N | Y | Y | N | N | Joint workshop with EUPHA-DH and EUPHA-HTA on assessment and regulation of health management apps. | <https://eupha.org/repository/sections/dh/EUPHA-DH%20Annual%20report%202020.pdf> | N |
| Workshop: Digital Interventions in public mental health: current impact and future directions | European Public Health Association (EUPHA); World Federation of Public Health Associations (WFPHA) | International organisations | English | Scientific Association/Society | Conference session | 16th World Conference of Public Health 2020 | Online | 2020 | Y | Y | Y | N | N | Y | N | N | Joint workshop with EUPHA-DH and EUPHA-PMH on digital interventions in public mental health. | <https://academic.oup.com/eurpub/article/30/Supplement_5/ckaa165.1078/5915594> | N |
| Workshop: How are digital solutions contributing to enhance immunization programmes and policies? | European Public Health Association (EUPHA); World Federation of Public Health Associations (WFPHA) | International organisations | English | Scientific Association/Society | Conference session | 16th World Conference of Public Health 2020 | Online | 2020 | Y | N | Y | N | N | N | N | N | Joint workshop with EUPHA-DH and EUPHA-IDC on digital solutions for immunization programmes. | <https://academic.oup.com/eurpub/article/30/Supplement_5/ckaa165.005/5913699> | N |
| Plenary 3: Learning from the pandemic and getting ready for the next one | European Public Health Association (EUPHA) | International organisations | English | Scientific Association/Society | Conference session | 14th EPH Conference 2021 | Online | 2021 | Y | N | Y | N | Y | N | N | N | Plenary session on lessons learned from the pandemic and preparedness for the next one. | <https://academic.oup.com/eurpub/article/31/Supplement_3/ckab166.003/6406320> | N |
| Plenary 4: Capturing the breadth and depth of the digital health era - beyond the COVID-19 pandemic | European Public Health Association (EUPHA) | International organisations | English | Scientific Association/Society | Conference session | 14th EPH Conference 2021 | Online | 2021 | Y | N | Y | N | Y | Y | Y | N | Plenary session on the breadth and depth of the digital health era beyond the COVID-19 pandemic. | [https://eupha.org/repository/sections/dh/DH%20Annual%20report%20EUPHA%20sections%202021.pdf / https://db.eupha.org/repository/advocacy/2022/Track%20report%20-%20Digitalization%20in%20health%20in%202021.pdf](https://eupha.org/repository/sections/dh/DH%20Annual%20report%20EUPHA%20sections%202021.pdf) | N |
| Round table: Digital innovation to fight the pandemic, what is here to stay? | European Public Health Association (EUPHA) | International organisations | English | Scientific Association/Society | Conference session | 14th EPH Conference 2021 | Online | 2021 | Y | N | Y | N | Y | N | N | N | Roundtable organised by EUPHA-DH on digital innovation to fight the pandemic and what is here to stay. | <https://academic.oup.com/eurpub/article/31/Supplement_3/ckab164.625/6405781> | N |
| Round table: EU Digital COVID Certificate: Hope for restoring freedom or simply opening Pandora's box? | European Public Health Association (EUPHA) | International organisations | English | Scientific Association/Society | Conference session | 14th EPH Conference 2021 | Online | 2021 | N | N | N | N | Y | Y | N | N | Roundtable on the EU Digital COVID Certificate. | <https://academic.oup.com/eurpub/article/31/Supplement_3/ckab164.825/6405536> | N |
| Round table: Governing digital transformations in health: Shaping the digital future of Europe | European Public Health Association (EUPHA) | International organisations | English | Scientific Association/Society | Conference session | 14th EPH Conference 2021 | Online | 2021 | N | Y | Y | N | N | Y | N | N | Roundtable on governance of digital transformations in health. | <https://academic.oup.com/eurpub/article/31/Supplement_3/ckab164.549/6404880> | N |
| Round table: How to prepare the public health workforce for a digital health future? | European Public Health Association (EUPHA) | International organisations | English | Scientific Association/Society | Conference session | 14th EPH Conference 2021 | Online | 2021 | Y | N | Y | N | N | N | N | N | Roundtable on preparing the public health workforce for a digital health future. | <https://pmc.ncbi.nlm.nih.gov/articles/PMC8574714/> | N |
| Skill building seminar: AI for public health dummies - overview and a benchmark algorithm: Random Forests | European Public Health Association (EUPHA) | International organisations | English | Scientific Association/Society | Conference session | 14th EPH Conference 2021 | Online | 2021 | Y | N | N | Y | N | N | N | Y | Skill-building seminar on AI for public health beginners, with overview and benchmark algorithm (Random Forests). | <https://academic.oup.com/eurpub/article/31/Supplement_3/ckab164.568/6406020> | N |
| Workshop: Assessment, regulation and use of apps for health management: where we are and where we are going? | European Public Health Association (EUPHA) | International organisations | English | Scientific Association/Society | Conference session | 14th EPH Conference 2021 | Online | 2021 | N | N | Y | N | N | Y | N | N | Workshop on assessment and regulation of health management apps. | <https://academic.oup.com/eurpub/article/30/Supplement_5/ckaa165.1326/5915100> | N |
| Workshop: COVID-HL: A global survey on digital health literacy in university students during the pandemic | European Public Health Association (EUPHA) | International organisations | English | Scientific Association/Society | Conference session | 14th EPH Conference 2021 | Online | 2021 | N | Y | N | N | N | N | N | N | Workshop on COVID-HL, a global survey on digital health literacy among university students during the pandemic. | <https://academic.oup.com/eurpub/article/31/Supplement_3/ckab164.037/6405418> | N |
| Workshop: Developing a consent model for health information - challenges in a rapidly changing digital world | European Public Health Association (EUPHA) | International organisations | English | Scientific Association/Society | Conference session | 14th EPH Conference 2021 | Online | 2021 | N | N | Y | N | Y | Y | N | N | Workshop on developing consent models for health information in a digital world. | <https://academic.oup.com/eurpub/article/31/Supplement_3/ckab164.428/6405192> | N |
| Workshop: Looking beyond COVID-19: Putting people at the centre in the digital health era | European Public Health Association (EUPHA) | International organisations | English | Scientific Association/Society | Conference session | 14th EPH Conference 2021 | Online | 2021 | N | Y | Y | N | N | Y | Y | N | Workshop on putting people at the centre in the digital health era beyond COVID-19. | <https://pmc.ncbi.nlm.nih.gov/articles/PMC8574700/> | N |
| Workshop: The European Health Data Space (EHDS): future opportunities and current challenges | European Public Health Association (EUPHA) | International organisations | English | Scientific Association/Society | Conference session | 14th EPH Conference 2021 | Online | 2021 | N | N | N | N | Y | Y | N | N | Workshop on the European Health Data Space (EHDS): opportunities and challenges. | <https://pmc.ncbi.nlm.nih.gov/articles/PMC8574771/> | N |
| Workshop: The Role of Digital Public Health in the European Climate Pact and Green deal | European Public Health Association (EUPHA) | International organisations | English | Scientific Association/Society | Conference session | 14th EPH Conference 2021 | Online | 2021 | N | N | Y | N | N | N | N | N | Workshop on the role of digital public health in the European Climate Pact and Green Deal. | <https://pmc.ncbi.nlm.nih.gov/articles/PMC8574567/> | N |
| Plenary: Informing decisions across Europe: Building sustainable infrastructure for cross-country health information exchange | European Health Management Association (EHMA) | International organisations | English | Scientific Association/Society | Conference session | EHMA 2022 Annual Conference | In presence | 2022 | Y | N | Y | N | Y | N | N | N | Plenary session on cross-border health information exchange and digital infrastructure for public health data. | <https://2022.ehmaconference.org/ehma-2022-conference-programme/> | N |
| Workshop: Building resilient and sustainable health systems: Lessons from COVID-19 - insights from the State of Health in the EU Country Profiles | European Health Management Association (EHMA) | International organisations | English | Scientific Association/Society | Conference session | EHMA 2022 Annual Conference | In presence | 2022 | Y | Y | Y | N | N | N | N | N | Workshop on resilient health systems, digital innovation and workforce strategies post-COVID, with insights from the State of Health in the EU Country Profiles. | <https://2022.ehmaconference.org/ehma-2022-conference-programme/> | N |
| Interactive Session: COVIRNA: Understanding the role of health managers in implementing innovative diagnostic tools to improve patient-centred care | European Health Management Association (EHMA) | International organisations | English | Scientific Association/Society | Conference session | EHMA 2022 Annual Conference | In presence | 2022 | Y | N | Y | N | N | N | N | N | Interactive session on the role of health managers in implementing innovative diagnostic tools (COVIRNA project). | <https://2022.ehmaconference.org/ehma-2022-conference-programme/> | N |
| Workshop: JADECARE: Towards digitally enabled integrated person-centred care | European Health Management Association (EHMA) | International organisations | English | Scientific Association/Society | Conference session | EHMA 2022 Annual Conference | In presence | 2022 | Y | Y | Y | N | N | N | N | N | Workshop on transferring best practices for digitally-enabled integrated person-centred care (JADECARE project). | <https://2022.ehmaconference.org/ehma-2022-conference-programme/> | N |
| Closing Plenary: Leading healthcare into the future: Shaping tomorrow's healthcare today | European Health Management Association (EHMA) | International organisations | English | Scientific Association/Society | Conference session | EHMA 2022 Annual Conference | In presence | 2022 | Y | Y | Y | N | N | N | N | N | Closing plenary on drivers of change in health systems and the role of health leadership. | <https://2022.ehmaconference.org/ehma-2022-conference-programme/> | N |
| Workshop: International Health and Social Care Collaborative: Partnership working across borders | European Health Management Association (EHMA) | International organisations | English | Scientific Association/Society | Conference session | EHMA 2022 Annual Conference | In presence | 2022 | N | N | Y | N | N | N | N | N | Workshop on international collaboration in health and social care partnerships (USA, Belgium, UK). | <https://2022.ehmaconference.org/ehma-2022-conference-programme/> | N |
| Plenary 2: Benefits and challenges of the European Health Data Space | European Public Health Association (EUPHA) | International organisations | English | Scientific Association/Society | Conference session | 15th EPH Conference 2022 | Online | 2022 | N | N | N | N | Y | Y | N | N | Plenary session on benefits and challenges of the European Health Data Space. | <https://eupha.org/repository/sections/dh/DH_Annual%20report%20EUPHA%20sections%202022.pdf> | N |
| Round table: Better DiPH - To plan, implement, evaluate, and the future of digital public health interventions | European Public Health Association (EUPHA) | International organisations | English | Scientific Association/Society | Conference session | 15th EPH Conference 2022 | Online | 2022 | Y | N | Y | Y | N | N | N | N | Roundtable on planning, implementing and evaluating digital public health interventions (Better DiPH). | <https://eupha.org/repository/sections/dh/DH_Annual%20report%20EUPHA%20sections%202022.pdf> | N |
| Skills building seminar: Digital health, informatics, and public health | European Public Health Association (EUPHA) | International organisations | English | Scientific Association/Society | Conference session | 15th EPH Conference 2022 | Online | 2022 | Y | Y | Y | N | N | N | N | N | Seminar on digital health and informatics in public health. | <https://eupha.org/repository/sections/dh/DH_Annual%20report%20EUPHA%20sections%202022.pdf> | N |
| Skills building seminar: Health Data Pipelines: moving away from Excel to scalable, insightful and future-proof infrastructure | European Public Health Association (EUPHA) | International organisations | English | Scientific Association/Society | Conference session | 15th EPH Conference 2022 | Online | 2022 | Y | N | N | Y | Y | N | N | N | Seminar on health data pipelines: moving from Excel to scalable infrastructure. | <https://eupha.org/repository/sections/dh/DH_Annual%20report%20EUPHA%20sections%202022.pdf> | N |
| Skills building seminar: Podcast 101 | European Public Health Association (EUPHA) | International organisations | English | Scientific Association/Society | Conference session | 15th EPH Conference 2022 | Online | 2022 | Y | N | N | N | N | N | Y | N | Seminar introducing podcast creation for public health communication. | <https://eupha.org/repository/sections/dh/DH_Annual%20report%20EUPHA%20sections%202022.pdf> | N |
| Introductory open science workshop on research data management | DDS-MAP | International organisations | English | Scientific Association/Society | Seminar/Webinar | DDS-MAP Project | Online | 2023 | N | N | N | Y | Y | N | N | N | Hands-on online workshop on research data management for consortium partners and researchers. | <https://ddsmap.easpd.eu/category/news/> | N |
| Plenary 4: On the path to an equitable and sustainable digital future for European health systems | European Public Health Association (EUPHA) | International organisations | English | Scientific Association/Society | Conference session | 16th EPH Conference 2023 | In presence | 2023 | N | N | Y | N | N | Y | N | N | Plenary session on an equitable and sustainable digital future for European health systems. | <https://eupha.org/repository/sections/dh/DH_Annual%20report%20EUPHA%20sections%202023.pdf> | N |
| Pre-conference: Better public health: a data centred approach to interoperability, with international information standards | European Public Health Association (EUPHA) | International organisations | English | Scientific Association/Society | Conference session | 16th EPH Conference 2023 | In presence | 2023 | N | N | Y | Y | Y | Y | N | N | Pre-conference on data interoperability and international information standards for public health. | <https://eupha.org/repository/sections/dh/DH_Annual%20report%20EUPHA%20sections%202023.pdf> | N |
| Workshop: Digital Public Health and Climate change: The European Framework made easy | European Public Health Association (EUPHA) | International organisations | English | Scientific Association/Society | Conference session | 16th EPH Conference 2023 | In presence | 2023 | N | N | Y | N | N | Y | N | N | Workshop on the European framework for digital public health and climate change. | <https://eupha.org/repository/sections/dh/DH_Annual%20report%20EUPHA%20sections%202023.pdf> | N |
| Workshop: Digital public health in Europe - What is the new normal? | European Public Health Association (EUPHA) | International organisations | English | Scientific Association/Society | Conference session | 16th EPH Conference 2023 | In presence | 2023 | Y | N | Y | N | N | Y | N | N | Workshop on how COVID-19 changed health systems in Romania, Ireland, Slovenia and Germany, discussing which digital transformations should remain. | <https://pmc.ncbi.nlm.nih.gov/articles/PMC10596705/> | N |
| Workshop: Innovations for chronic diseases management: complementing usual care with digital therapeutics | European Public Health Association (EUPHA) | International organisations | English | Scientific Association/Society | Conference session | 16th EPH Conference 2023 | In presence | 2023 | Y | N | Y | N | N | Y | N | N | Workshop on innovations for chronic disease management with digital therapeutics. | <https://eupha.org/repository/sections/dh/DH_Annual%20report%20EUPHA%20sections%202023.pdf> | N |
| Workshop: Digital health literacy in childhood and adolescence: concepts, assessment tools, and study results | European Public Health Association (EUPHA) | International organisations | English | Scientific Association/Society | Conference session | 16th EPH Conference 2023 | In presence | 2023 | N | Y | N | N | N | N | N | N | Workshop on digital health literacy in childhood and adolescence: concepts, assessment tools, and representative data on DHL levels among students and teachers. | <https://doi.org/10.1093/eurpub/ckad160.120> | N |
| Universal access to care - How can we overcome barriers in the patient's care journey? | European Public Health Association (EUPHA); European Observatory on Health Systems and Policies | International organisations | English | Scientific Association/Society | Seminar/Webinar | European Public Health WEEK 2023 | Online | 2023 | Y | Y | Y | N | N | N | N | N | Webinar on universal access to care and overcoming barriers in the patient's care journey, held during European Public Health Week. | <https://eupha.org/access_to_care> | N |
| Advancing inclusivity in health care through the use of telehealth | WHO Regional Office for Europe | International organisations | English | Scientific Association/Society | Seminar/Webinar |  | Online | 2023 | Y | Y | Y | N | N | N | N | N | Webinar on how telehealth can promote inclusivity in healthcare. | <https://www.who.int/europe/news-room/events/item/2023/06/20/default-calendar/advancing-inclusivity-in-health-care-through-the-use-of-telehealth> | N |
| Fireside chat: Artificial intelligence and its regulation | WHO Regional Office for Europe | International organisations | English | Scientific Association/Society | Conference session | 2nd WHO Symposium on the Future of Health Systems in a Digital Era in the European Region | In presence | 2023 | N | N | N | N | N | Y | N | Y | Fireside chat on AI regulation in healthcare. | [https://www.who.int/europe/news-room/events/item/2023/09/05/default-calendar/second-who-symposium-on-the-future-of-digital-health-systems-in-the-european-region / https://www.who.int/europe/publications/second-who-symposium-on-the-future-of-digital-health-systems-in-the-european-region-provisional-programme](https://www.who.int/europe/news-room/events/item/2023/09/05/default-calendar/second-who-symposium-on-the-future-of-digital-health-systems-in-the-european-region) | N |
| Ministerial Panel: Trust and transformation of health systems in the digital age | WHO Regional Office for Europe | International organisations | English | Scientific Association/Society | Conference session | 2nd WHO Symposium on the Future of Health Systems in a Digital Era in the European Region | In presence | 2023 | N | Y | Y | N | N | Y | N | N | Ministerial panel on trust and digital transformation in health systems. | [https://www.who.int/europe/news-room/events/item/2023/09/05/default-calendar/second-who-symposium-on-the-future-of-digital-health-systems-in-the-european-region / https://www.who.int/europe/publications/second-who-symposium-on-the-future-of-digital-health-systems-in-the-european-region-provisional-programme](https://www.who.int/europe/news-room/events/item/2023/09/05/default-calendar/second-who-symposium-on-the-future-of-digital-health-systems-in-the-european-region) | N |
| Opening Plenary: Global developments in digital health | WHO Regional Office for Europe | International organisations | English | Scientific Association/Society | Conference session | 2nd WHO Symposium on the Future of Health Systems in a Digital Era in the European Region | In presence | 2023 | Y | Y | Y | N | N | N | N | N | Opening plenary on global developments in digital health, with launch of the WHO Europe report on digital transformation. | [https://www.who.int/europe/news-room/events/item/2023/09/05/default-calendar/second-who-symposium-on-the-future-of-digital-health-systems-in-the-european-region / https://www.who.int/europe/publications/second-who-symposium-on-the-future-of-digital-health-systems-in-the-european-region-provisional-programme](https://www.who.int/europe/news-room/events/item/2023/09/05/default-calendar/second-who-symposium-on-the-future-of-digital-health-systems-in-the-european-region) | N |
| Panel 1: Artificial Intelligence: gAIn or pAIn for European Health Systems? | WHO Regional Office for Europe | International organisations | English | Scientific Association/Society | Conference session | 2nd WHO Symposium on the Future of Health Systems in a Digital Era in the European Region | In presence | 2023 | N | N | Y | N | N | Y | N | Y | Panel on AI opportunities and challenges for European health systems. | [https://www.who.int/europe/news-room/events/item/2023/09/05/default-calendar/second-who-symposium-on-the-future-of-digital-health-systems-in-the-european-region / https://www.who.int/europe/publications/second-who-symposium-on-the-future-of-digital-health-systems-in-the-european-region-provisional-programme](https://www.who.int/europe/news-room/events/item/2023/09/05/default-calendar/second-who-symposium-on-the-future-of-digital-health-systems-in-the-european-region) | N |
| Panel 2: Transforming digital health practice: The critical role of governance, investment and evaluation | WHO Regional Office for Europe | International organisations | English | Scientific Association/Society | Conference session | 2nd WHO Symposium on the Future of Health Systems in a Digital Era in the European Region | In presence | 2023 | N | N | Y | N | Y | Y | N | N | Panel on governance, investment and evaluation in digital health. | [https://www.who.int/europe/news-room/events/item/2023/09/05/default-calendar/second-who-symposium-on-the-future-of-digital-health-systems-in-the-european-region / https://www.who.int/europe/publications/second-who-symposium-on-the-future-of-digital-health-systems-in-the-european-region-provisional-programme](https://www.who.int/europe/news-room/events/item/2023/09/05/default-calendar/second-who-symposium-on-the-future-of-digital-health-systems-in-the-european-region) | N |
| Panel 3: Putting equity and inclusion at the heart of European health systems of the future | WHO Regional Office for Europe | International organisations | English | Scientific Association/Society | Conference session | 2nd WHO Symposium on the Future of Health Systems in a Digital Era in the European Region | In presence | 2023 | N | Y | Y | N | N | Y | N | N | Panel on equity and inclusion in future European health systems. | [https://www.who.int/europe/news-room/events/item/2023/09/05/default-calendar/second-who-symposium-on-the-future-of-digital-health-systems-in-the-european-region / https://www.who.int/europe/publications/second-who-symposium-on-the-future-of-digital-health-systems-in-the-european-region-provisional-programme](https://www.who.int/europe/news-room/events/item/2023/09/05/default-calendar/second-who-symposium-on-the-future-of-digital-health-systems-in-the-european-region) | N |
| Session 1.1: Knowing me, Knowing you – patients at the centre of their own digital care | WHO Regional Office for Europe | International organisations | English | Scientific Association/Society | Conference session | 2nd WHO Symposium on the Future of Health Systems in a Digital Era in the European Region | In presence | 2023 | Y | Y | N | N | N | N | N | N | Session on patient-centred digital care approaches. | [https://www.who.int/europe/news-room/events/item/2023/09/05/default-calendar/second-who-symposium-on-the-future-of-digital-health-systems-in-the-european-region / https://www.who.int/europe/publications/second-who-symposium-on-the-future-of-digital-health-systems-in-the-european-region-provisional-programme](https://www.who.int/europe/news-room/events/item/2023/09/05/default-calendar/second-who-symposium-on-the-future-of-digital-health-systems-in-the-european-region) | N |
| Session 1.2: Voices of tomorrow: harnessing the power of youth innovation in digital health | WHO Regional Office for Europe | International organisations | English | Scientific Association/Society | Conference session | 2nd WHO Symposium on the Future of Health Systems in a Digital Era in the European Region | In presence | 2023 | Y | Y | Y | N | N | N | N | N | Session on youth-driven innovation in digital health. | [https://www.who.int/europe/news-room/events/item/2023/09/05/default-calendar/second-who-symposium-on-the-future-of-digital-health-systems-in-the-european-region / https://www.who.int/europe/publications/second-who-symposium-on-the-future-of-digital-health-systems-in-the-european-region-provisional-programme](https://www.who.int/europe/news-room/events/item/2023/09/05/default-calendar/second-who-symposium-on-the-future-of-digital-health-systems-in-the-european-region) | N |
| Session 1.3: From disconnection to empowerment in the digital age: Exploring age-friendly environments and technological solutions | WHO Regional Office for Europe | International organisations | English | Scientific Association/Society | Conference session | 2nd WHO Symposium on the Future of Health Systems in a Digital Era in the European Region | In presence | 2023 | Y | Y | N | N | N | N | N | N | Session on digital empowerment and age-friendly technological solutions. | [https://www.who.int/europe/news-room/events/item/2023/09/05/default-calendar/second-who-symposium-on-the-future-of-digital-health-systems-in-the-european-region / https://www.who.int/europe/publications/second-who-symposium-on-the-future-of-digital-health-systems-in-the-european-region-provisional-programme](https://www.who.int/europe/news-room/events/item/2023/09/05/default-calendar/second-who-symposium-on-the-future-of-digital-health-systems-in-the-european-region) | N |
| Session 1.4: High-tech & High-touch: Can digital technologies mitigate the health workforce crisis? | WHO Regional Office for Europe | International organisations | English | Scientific Association/Society | Conference session | 2nd WHO Symposium on the Future of Health Systems in a Digital Era in the European Region | In presence | 2023 | Y | N | Y | N | N | N | N | N | Session on whether digital technologies can mitigate the health workforce crisis. | [https://www.who.int/europe/news-room/events/item/2023/09/05/default-calendar/second-who-symposium-on-the-future-of-digital-health-systems-in-the-european-region / https://www.who.int/europe/publications/second-who-symposium-on-the-future-of-digital-health-systems-in-the-european-region-provisional-programme](https://www.who.int/europe/news-room/events/item/2023/09/05/default-calendar/second-who-symposium-on-the-future-of-digital-health-systems-in-the-european-region) | N |
| Session 1.5: Online health mis- and disinformation: how to be better prepared for the next Infodemic together | WHO Regional Office for Europe | International organisations | English | Scientific Association/Society | Conference session | 2nd WHO Symposium on the Future of Health Systems in a Digital Era in the European Region | In presence | 2023 | N | Y | N | N | N | N | Y | N | Session on online health misinformation and preparedness for future infodemics. | [https://www.who.int/europe/news-room/events/item/2023/09/05/default-calendar/second-who-symposium-on-the-future-of-digital-health-systems-in-the-european-region / https://www.who.int/europe/publications/second-who-symposium-on-the-future-of-digital-health-systems-in-the-european-region-provisional-programme](https://www.who.int/europe/news-room/events/item/2023/09/05/default-calendar/second-who-symposium-on-the-future-of-digital-health-systems-in-the-european-region) | N |
| Session 2.1: Unlocking the potential of digital health to fight noncommunicable disease | WHO Regional Office for Europe | International organisations | English | Scientific Association/Society | Conference session | 2nd WHO Symposium on the Future of Health Systems in a Digital Era in the European Region | In presence | 2023 | Y | N | Y | N | N | N | N | N | Session on digital health solutions for non-communicable diseases. | [https://www.who.int/europe/news-room/events/item/2023/09/05/default-calendar/second-who-symposium-on-the-future-of-digital-health-systems-in-the-european-region / https://www.who.int/europe/publications/second-who-symposium-on-the-future-of-digital-health-systems-in-the-european-region-provisional-programme](https://www.who.int/europe/news-room/events/item/2023/09/05/default-calendar/second-who-symposium-on-the-future-of-digital-health-systems-in-the-european-region) | N |
| Session 2.2: Digital health for health emergency preparedness 2.0 in the WHO European Region | WHO Regional Office for Europe | International organisations | English | Scientific Association/Society | Conference session | 2nd WHO Symposium on the Future of Health Systems in a Digital Era in the European Region | In presence | 2023 | Y | N | Y | Y | Y | N | N | N | Session on digital health for health emergency preparedness in the WHO European Region. | [https://www.who.int/europe/news-room/events/item/2023/09/05/default-calendar/second-who-symposium-on-the-future-of-digital-health-systems-in-the-european-region / https://www.who.int/europe/publications/second-who-symposium-on-the-future-of-digital-health-systems-in-the-european-region-provisional-programme](https://www.who.int/europe/news-room/events/item/2023/09/05/default-calendar/second-who-symposium-on-the-future-of-digital-health-systems-in-the-european-region) | N |
| Session 2.3: Digital technologies and One Health: balancing and optimizing the health of people, animals and the environment | WHO Regional Office for Europe | International organisations | English | Scientific Association/Society | Conference session | 2nd WHO Symposium on the Future of Health Systems in a Digital Era in the European Region | In presence | 2023 | Y | N | N | Y | N | N | N | N | Session on digital technologies and the One Health approach. | [https://www.who.int/europe/news-room/events/item/2023/09/05/default-calendar/second-who-symposium-on-the-future-of-digital-health-systems-in-the-european-region / https://www.who.int/europe/publications/second-who-symposium-on-the-future-of-digital-health-systems-in-the-european-region-provisional-programme](https://www.who.int/europe/news-room/events/item/2023/09/05/default-calendar/second-who-symposium-on-the-future-of-digital-health-systems-in-the-european-region) | N |
| Session 2.4: Digital health solutions in the fight against Tuberculosis, HIV, and viral hepatitis in the WHO European Region | WHO Regional Office for Europe | International organisations | English | Scientific Association/Society | Conference session | 2nd WHO Symposium on the Future of Health Systems in a Digital Era in the European Region | In presence | 2023 | Y | N | N | N | N | N | N | N | Session on digital health solutions for TB, HIV and viral hepatitis in the WHO European Region. | [https://www.who.int/europe/news-room/events/item/2023/09/05/default-calendar/second-who-symposium-on-the-future-of-digital-health-systems-in-the-european-region / https://www.who.int/europe/publications/second-who-symposium-on-the-future-of-digital-health-systems-in-the-european-region-provisional-programme](https://www.who.int/europe/news-room/events/item/2023/09/05/default-calendar/second-who-symposium-on-the-future-of-digital-health-systems-in-the-european-region) | N |
| Session 2.5: Harnessing the power of emerging technologies for better mental health | WHO Regional Office for Europe | International organisations | English | Scientific Association/Society | Conference session | 2nd WHO Symposium on the Future of Health Systems in a Digital Era in the European Region | In presence | 2023 | Y | Y | N | N | N | N | N | N | Session on emerging technologies for mental health. | [https://www.who.int/europe/news-room/events/item/2023/09/05/default-calendar/second-who-symposium-on-the-future-of-digital-health-systems-in-the-european-region / https://www.who.int/europe/publications/second-who-symposium-on-the-future-of-digital-health-systems-in-the-european-region-provisional-programme](https://www.who.int/europe/news-room/events/item/2023/09/05/default-calendar/second-who-symposium-on-the-future-of-digital-health-systems-in-the-european-region) | N |
| Session 3.1: From big data to personalized care: How artificial intelligence and precision medicine are revolutionizing care | WHO Regional Office for Europe | International organisations | English | Scientific Association/Society | Conference session | 2nd WHO Symposium on the Future of Health Systems in a Digital Era in the European Region | In presence | 2023 | Y | N | N | Y | Y | N | N | Y | Session on AI and precision medicine for personalised care. | [https://www.who.int/europe/news-room/events/item/2023/09/05/default-calendar/second-who-symposium-on-the-future-of-digital-health-systems-in-the-european-region / https://www.who.int/europe/publications/second-who-symposium-on-the-future-of-digital-health-systems-in-the-european-region-provisional-programme](https://www.who.int/europe/news-room/events/item/2023/09/05/default-calendar/second-who-symposium-on-the-future-of-digital-health-systems-in-the-european-region) | N |
| Session 3.2: Beyond the walls: How can telehealth revolutionize healthcare? | WHO Regional Office for Europe | International organisations | English | Scientific Association/Society | Conference session | 2nd WHO Symposium on the Future of Health Systems in a Digital Era in the European Region | In presence | 2023 | Y | N | Y | N | N | N | N | N | Session on how telehealth can revolutionise healthcare. | [https://www.who.int/europe/news-room/events/item/2023/09/05/default-calendar/second-who-symposium-on-the-future-of-digital-health-systems-in-the-european-region / https://www.who.int/europe/publications/second-who-symposium-on-the-future-of-digital-health-systems-in-the-european-region-provisional-programme](https://www.who.int/europe/news-room/events/item/2023/09/05/default-calendar/second-who-symposium-on-the-future-of-digital-health-systems-in-the-european-region) | N |
| Session 3.3: Revolutionizing health information systems for data-driven decision making | WHO Regional Office for Europe | International organisations | English | Scientific Association/Society | Conference session | 2nd WHO Symposium on the Future of Health Systems in a Digital Era in the European Region | In presence | 2023 | Y | N | Y | Y | Y | N | N | N | Session on health information systems for data-driven decision-making. | [https://www.who.int/europe/news-room/events/item/2023/09/05/default-calendar/second-who-symposium-on-the-future-of-digital-health-systems-in-the-european-region / https://www.who.int/europe/publications/second-who-symposium-on-the-future-of-digital-health-systems-in-the-european-region-provisional-programme](https://www.who.int/europe/news-room/events/item/2023/09/05/default-calendar/second-who-symposium-on-the-future-of-digital-health-systems-in-the-european-region) | N |
| Session 3.4: Transforming health systems together: Views of the private sector on the future of digital health | WHO Regional Office for Europe | International organisations | English | Scientific Association/Society | Conference session | 2nd WHO Symposium on the Future of Health Systems in a Digital Era in the European Region | In presence | 2023 | N | N | Y | N | N | N | N | N | Session on private sector perspectives on the future of digital health. | [https://www.who.int/europe/news-room/events/item/2023/09/05/default-calendar/second-who-symposium-on-the-future-of-digital-health-systems-in-the-european-region / https://www.who.int/europe/publications/second-who-symposium-on-the-future-of-digital-health-systems-in-the-european-region-provisional-programme](https://www.who.int/europe/news-room/events/item/2023/09/05/default-calendar/second-who-symposium-on-the-future-of-digital-health-systems-in-the-european-region) | N |
| Session 3.5: Digital for Quality: how technology can enable individual contributions for quality of care | WHO Regional Office for Europe | International organisations | English | Scientific Association/Society | Conference session | 2nd WHO Symposium on the Future of Health Systems in a Digital Era in the European Region | In presence | 2023 | Y | N | Y | N | N | N | N | N | Session on how technology enables individual contributions to quality of care. | [https://www.who.int/europe/news-room/events/item/2023/09/05/default-calendar/second-who-symposium-on-the-future-of-digital-health-systems-in-the-european-region / https://www.who.int/europe/publications/second-who-symposium-on-the-future-of-digital-health-systems-in-the-european-region-provisional-programme](https://www.who.int/europe/news-room/events/item/2023/09/05/default-calendar/second-who-symposium-on-the-future-of-digital-health-systems-in-the-european-region) | N |
| Informatics and Data Science for Health (IDASH) Fellowship Programme - Cohort 1 | WHO Regional Office for Europe; CDC Eastern Europe and Central Asia | International organisations | English | Scientific Association/Society | Training course | IDASH Fellowship Programme | Hybrid | 2023 | Y | N | N | Y | Y | N | N | N | Annual fellowship for mid-to-senior public health staff on data analysis, visualisation, disease surveillance platforms and reporting systems. Fellows from Georgia, Kazakhstan, Kyrgyzstan, Ukraine and Uzbekistan. | <https://www.who.int/europe/news-room/17-05-2024-first-cohort-complete-who-and-cdc-informatics-and-data-science-for-health-course> | N |
| GenEpi-BioTrain: Genomic Epidemiology & Public Health Bioinformatics | European Centre for Disease Prevention and Control (ECDC) | International organisations | English | Scientific Association/Society | Training course |  | Online | 2024 | Y | N | N | Y | Y | N | N | N | E-learning course on bioinformatics tools (INSaFLU-TELEVIR), R data analysis, sequencing and phylogenetics for infectious disease public health professionals. | <https://learning.ecdc.europa.eu/> | N |
| Leveraging Digitalisation & AI in Healthcare | European Health Management Association (EHMA) | International organisations | English | Scientific Association/Society | Training course |  | Online | 2024 | Y | N | Y | N | N | N | N | Y | Training module on digitalisation and AI in healthcare for professionals and health management leaders. | <https://ehma.org/bocconi-learning-modules/> | N |
| Basics of Data Analytics | European Health Management Association (EHMA); BeWell Project | International organisations | English | Scientific Association/Society | Training course | BeWell Project | Online | 2024 | Y | N | N | Y | Y | N | N | Y | Introduction to machine learning for processing medical datasets, covering regression, classification and model validation. | <https://bewell-project.eu/bewell-courses/> | N |
| Clinical Databases - Big Data & Analytics Series Part 1 | European Health Management Association (EHMA); BeWell Project | International organisations | English | Scientific Association/Society | Training course | BeWell Project | Online | 2024 | Y | N | N | Y | Y | N | N | N | First of a three-part Big Data & Analytics series: clinical databases, data extraction and digital analysis for data-driven decision-making. | <https://bewell-project.eu/bewell-courses/> | N |
| Data Analytics with R | European Health Management Association (EHMA); BeWell Project | International organisations | English | Scientific Association/Society | Training course | BeWell Project | Online | 2024 | Y | N | N | Y | Y | N | N | Y | Course on implementing machine learning with R, with step-by-step examples participants can apply to their own data. | <https://bewell-project.eu/bewell-courses/> | N |
| Electronic Health Records Application | European Health Management Association (EHMA); BeWell Project | International organisations | English | Scientific Association/Society | Training course | BeWell Project | Online | 2024 | Y | N | N | N | Y | N | N | N | Course on electronic health records for improving care, health services and health promotion. | <https://bewell-project.eu/bewell-courses/> | N |
| Extended Reality in Healthcare | European Health Management Association (EHMA); BeWell Project | International organisations | English | Scientific Association/Society | Training course | BeWell Project | Online | 2024 | Y | N | N | N | N | N | N | N | Course on how extended reality is transforming nursing and clinical workflows, with practical insights on ethical and safe XR implementation. | <https://bewell-project.eu/bewell-courses/> | N |
| Healthcare-ICT | European Health Management Association (EHMA); BeWell Project | International organisations | English | Scientific Association/Society | Training course | BeWell Project | Online | 2024 | Y | N | N | N | Y | N | N | N | Five-module programme on digital health systems: EHR, digitalisation benefits, clinical data management with RIS/PACS, system architecture and technical implementation. | <https://bewell-project.eu/bewell-courses/> | N |
| Introductory Training Programme on Cybersecurity for Healthcare Staff | European Health Management Association (EHMA); BeWell Project | International organisations | English | Scientific Association/Society | Training course | BeWell Project | Online | 2024 | N | N | N | N | N | Y | N | N | Five-module programme (45 min each) on cybersecurity for healthcare staff: cyber threats, GDPR, password security, mobile device security, incident response. Includes videos, quizzes and reflection sessions. EQF levels 3-5. | <https://bewell-project.eu/bewell-courses/> | N |
| Microsoft Office and Google Suite for Healthcare | European Health Management Association (EHMA); BeWell Project | International organisations | English | Scientific Association/Society | Training course | BeWell Project | Online | 2024 | Y | N | N | N | N | N | N | N | Six-module course on Microsoft Office and Google Suite for healthcare work, with practical exercises. | <https://bewell-project.eu/bewell-courses/> | N |
| Problem-Solving with Digital Tools | European Health Management Association (EHMA); BeWell Project | International organisations | English | Scientific Association/Society | Training course | BeWell Project | Online | 2024 | Y | N | N | N | N | N | N | N | Module on problem-solving and digital teamwork skills for healthcare environments. | <https://bewell-project.eu/bewell-courses/> | N |
| Sustainable Logistics in Healthcare | European Health Management Association (EHMA); BeWell Project | International organisations | English | Scientific Association/Society | Training course | BeWell Project | Online | 2024 | Y | N | Y | N | N | N | N | N | Course on sustainable logistics in healthcare: supply chain management, change management and continuous improvement. | <https://bewell-project.eu/bewell-courses/> | N |
| Telemedicine and Telehealth | European Health Management Association (EHMA); BeWell Project | International organisations | English | Scientific Association/Society | Training course | BeWell Project | Online | 2024 | Y | N | N | N | N | Y | N | N | Course on telemedicine applications across healthcare services, covering technology, practical use, security, privacy and telemedicine's role in innovation and sustainability. | <https://bewell-project.eu/bewell-courses/> | N |
| Artificial Intelligence in Healthcare | European Public Health Association (EUPHA) | International organisations | English | Scientific Association/Society | Seminar/Webinar | Sailing to Lisbon 2024 webinar series | Online | 2024 | Y | Y | Y | N | N | Y | Y | Y | Webinar on AI in healthcare: training future professionals, misinformation risks, and the regulatory landscape. | <https://ephconference.eu/2024-sailing-to-lisbon-2024-621> | N |
| Data and Digital Public Health | European Public Health Association (EUPHA) | International organisations | English | Scientific Association/Society | Seminar/Webinar | Sailing to Lisbon 2024 webinar series | Online | 2024 | N | N | Y | Y | Y | Y | N | N | Webinar on data exchange challenges, legal aspects, and the need for an EU public health data strategy within the European Health Data Space. | <https://ephconference.eu/2024-sailing-to-lisbon-2024-621> | N |
| Plenary 1: Navigating the Artificial Intelligence (AI) wave: overcoming barriers and unleashing the potential of AI in transforming European public health | European Public Health Association (EUPHA) | International organisations | English | Scientific Association/Society | Conference session | 17th EPH Conference 2024 | In presence | 2024 | Y | N | Y | N | N | Y | N | Y | Plenary session on overcoming barriers and unleashing AI potential in European public health. | <https://eupha.org/repository/conference/2024/Programme%20book%20Lisbon%202024-WEB2.pdf> | N |
| Pre-conference: Developing curricula that empower the public health workforce to lead digital transformations in health | European Public Health Association (EUPHA) | International organisations | English | Scientific Association/Society | Conference session | 17th EPH Conference 2024 | In presence | 2024 | Y | Y | Y | N | N | N | N | N | Pre-conference on developing curricula to prepare the public health workforce for digital transformations. | <https://eupha.org/repository/conference/2024/Programme%20book%20Lisbon%202024-WEB2.pdf> | N |
| Pre-conference: Navigating the digital tide: preparing public health for the AI Revolution | European Public Health Association (EUPHA) | International organisations | English | Scientific Association/Society | Conference session | 17th EPH Conference 2024 | In presence | 2024 | Y | N | Y | N | N | Y | N | Y | Pre-conference on preparing public health for the AI revolution. | <https://eupha.org/repository/conference/2024/Programme%20book%20Lisbon%202024-WEB2.pdf> | N |
| Data and Digital Public Health | European Public Health Association (EUPHA) | International organisations | English | Scientific Association/Society | Seminar/Webinar | Sailing to Lisbon 2024 webinar series | Online | 2024 | N | N | Y | N | Y | Y | N | N | Webinar on data exchange challenges, legal aspects, and the need for an EU public health data strategy within the European Health Data Space. | <https://ephconference.eu/2024-sailing-to-lisbon-2024-621> | N |
| Round table: Artificial Intelligence in Mobile Health Apps: Ethical, Legal, and Regulatory Challenges | European Public Health Association (EUPHA) | International organisations | English | Scientific Association/Society | Conference session | 17th EPH Conference 2024 | In presence | 2024 | N | N | N | N | N | Y | N | Y | Roundtable on ethical, legal and regulatory challenges of AI in mobile health apps. | <https://eupha.org/repository/conference/2024/Programme%20book%20Lisbon%202024-WEB2.pdf> | N |
| Round table: Closing the loop in the European Health Data Space: relation between primary and secondary data use | European Public Health Association (EUPHA) | International organisations | English | Scientific Association/Society | Conference session | 17th EPH Conference 2024 | In presence | 2024 | N | N | N | N | Y | Y | N | N | Roundtable on the relationship between primary and secondary data use in the European Health Data Space. | <https://eupha.org/repository/conference/2024/Programme%20book%20Lisbon%202024-WEB2.pdf> | N |
| Round table: Social media & smartphones: Threats to child & adolescent health and public health solutions | European Public Health Association (EUPHA) | International organisations | English | Scientific Association/Society | Conference session | 17th EPH Conference 2024 | In presence | 2024 | N | Y | N | N | N | N | Y | N | Roundtable on threats of social media and smartphones to child and adolescent health. | <https://eupha.org/repository/conference/2024/Programme%20book%20Lisbon%202024-WEB2.pdf> | N |
| Workshop: Promoting health in the digital age: a room to improve health literacy and digital health literacy? | European Public Health Association (EUPHA) | International organisations | English | Scientific Association/Society | Conference session | 17th EPH Conference 2024 | In presence | 2024 | N | Y | N | N | N | N | N | N | Workshop on health promotion in the digital age and health literacy. | <https://academic.oup.com/eurpub/article/34/Supplement_3/ckae144.552/7843787> | N |
| Strengthening telemedicine: sustainable practices and policy pathways for health systems | WHO Regional Office for Europe | International organisations | English | Scientific Association/Society | Seminar/Webinar | Decoding Data and Digital Health Webinar Series | Online | 2024 | Y | N | Y | N | Y | N | N | N | Webinar (WHO/Europe and OECD) on telemedicine frameworks, tools and best practices for sustainable implementation in post-pandemic health systems. | <https://www.who.int/europe/news-room/events/item/2025/03/26/default-calendar/strengthening-telemedicine--sustainable-practices-and-policy-pathways-for-health-systems> | N |
| Digital health reimbursement: towards systematic and sustainable solutions | WHO Regional Office for Europe | International organisations | English | Scientific Association/Society | Seminar/Webinar | Decoding Data and Digital Health Webinar Series | Online | 2024 | N | N | Y | N | N | Y | N | N | Webinar on digital health reimbursement and sustainable solutions. | <https://www.who.int/europe/news-room/events/item/2024/04/18/default-calendar/digital-health-reimbursement--towards-systematic-and-sustainable-solutions> | N |
| Enhancing cybersecurity in health: protecting digital health information systems | WHO Regional Office for Europe | International organisations | English | Scientific Association/Society | Seminar/Webinar | Decoding Data and Digital Health Webinar Series | Online | 2024 | N | N | N | N | Y | Y | N | N | Webinar on cybersecurity in health and protecting digital health information systems. | <https://www.who.int/europe/news-room/events/item/2024/07/18/default-calendar/enhancing-cybersecurity-in-health--protecting-digital-health-information-systems> | N |
| Enhancing trust in AI for health through good practices, transparency and governance | WHO Regional Office for Europe | International organisations | English | Scientific Association/Society | Seminar/Webinar | Decoding Data and Digital Health Webinar Series | Online | 2024 | N | N | N | N | N | Y | N | Y | Webinar on trust in AI for health through good practices, transparency and governance. | <https://www.who.int/europe/news-room/events/item/2024/02/28/default-calendar/enhancing-trust-in-ai-for-health-through-good-practices--transparency-and-governance> | N |
| Optimizing the health and care workforce through data and digital health | WHO Regional Office for Europe | International organisations | English | Scientific Association/Society | Seminar/Webinar | Decoding Data and Digital Health Webinar Series | Online | 2024 | Y | N | Y | Y | Y | N | N | N | Webinar on optimising the health and care workforce through data and digital health. | <https://www.who.int/europe/news-room/events/item/2024/06/06/default-calendar/optimizing-the-health-and-care-workforce-through-data-and-digital-health> | N |
| Promoting healthy ageing in a digital world | WHO Regional Office for Europe | International organisations | English | Scientific Association/Society | Seminar/Webinar | Decoding Data and Digital Health Webinar Series | Online | 2024 | Y | Y | N | N | N | N | N | N | Webinar on promoting healthy ageing in a digital world. | <https://www.who.int/europe/news-room/events/item/2024/09/17/default-calendar/promoting-healthy-ageing-in-a-digital-world> | N |
| Strengthening digital health literacy to empower people in the digital age | WHO Regional Office for Europe | International organisations | English | Scientific Association/Society | Seminar/Webinar | Decoding Data and Digital Health Webinar Series | Online | 2024 | N | Y | N | N | N | N | N | N | Webinar on digital health literacy to empower people in the digital age. | <https://www.who.int/europe/news-room/events/item/2024/11/21/default-calendar/strengthening-digital-health-literacy-to-empower-people-in-the-digital-age> | N |
| Informatics and Data Science for Health (IDASH) Fellowship Programme - Cohort 2 | WHO Regional Office for Europe; CDC Eastern Europe and Central Asia | International organisations | English | Scientific Association/Society | Training course | IDASH Fellowship Programme | Hybrid | 2024 | Y | N | N | Y | Y | N | N | N | Annual fellowship for mid-to-senior public health staff on data analysis, visualisation, disease surveillance platforms and reporting systems. Fellows from Georgia, Kazakhstan, Kyrgyzstan, Republic of Moldova, Ukraine and Uzbekistan. | <https://www.who.int/europe/news-room/17-05-2024-first-cohort-complete-who-and-cdc-informatics-and-data-science-for-health-course> | N |
| Workshop: Cybersecurity crisis management simulation | Brunswick Group | International organisations | English | Scientific Association/Society | Conference session | EHMA 2025 Annual Conference | In presence | 2025 | N | N | Y | N | N | Y | N | N | Interactive workshop for hospital management teams on cyber incident impacts, testing response protocols and crisis decision-making. | <https://ehmaconference.org/ehma-2025-conference-programme/> | N |
| Building a digitally ready health workforce: DDS-MAP Final European Conference | DDS-MAP; South East Technological University (SETU) | International organisations | English | School/University | Conference session | DDS-MAP Project Final Conference | In presence | 2025 | Y | Y | Y | N | Y | Y | N | N | Final DDS-MAP conference in Brussels on digital skills for healthcare professionals, cybersecurity, lifelong learning and pan-European micro-credentials framework. | <https://ddsmap.easpd.eu/2025/07/08/building-a-digitally-ready-health-workforce-dds-map-conference-wraps-up-in-brussels/> | N |
| TEHDAS2 Joint Action: Workshop on the ethical dimensions of the European Health Data Space (EHDS) | European Commission - DG SANTE | International organisations | English | Scientific Association/Society | Seminar/Webinar |  | Online | 2025 | N | N | N | N | Y | Y | N | N | TEHDAS2 workshop on ethical dimensions of the European Health Data Space: data privacy, consent and equitable access. | <https://health.ec.europa.eu/latest-updates/registration-tehdas2-joint-action-workshop-ethical-dimensions-european-health-data-space-ehds-23-2025-06-02_en> | N |
| EUHPP Webinar: Using GenAI to Unlock the Value of Unstructured Health Data for EHDS | European Commission - DG SANTE | International organisations | English | Scientific Association/Society | Seminar/Webinar |  | Online | 2025 | Y | N | N | Y | N | N | N | Y | Webinar on using generative AI to extract value from unstructured health data (clinical notes, reports) in the EHDS context. | <https://health.ec.europa.eu/latest-updates/agenda-euhpp-webinar-using-genai-unlock-value-unstructured-health-data-ehds-16-june-2025-1430-1530-2025-06-10_en> | N |
| European Health Data Space (EHDS) Webinar 1: Primary Use of Health Data and Electronic Health Record Systems | European Commission - DG SANTE | International organisations | English | Scientific Association/Society | Seminar/Webinar | European Health Data Space (EHDS) Webinar Series | Online | 2025 | N | N | N | Y | Y | Y | N | N | First EHDS webinar on primary use of health data and electronic health record systems. | <https://health.ec.europa.eu/ehealth-digital-health-and-care/european-health-data-space-regulation-ehds/european-health-data-space-ehds-webinar-series_en> | N |
| European Health Data Space (EHDS) Webinar 2: Secondary Use of Health Data | European Commission - DG SANTE | International organisations | English | Scientific Association/Society | Seminar/Webinar | European Health Data Space (EHDS) Webinar Series | Online | 2025 | N | N | N | Y | Y | Y | N | N | Second EHDS webinar on secondary use of health data. | <https://health.ec.europa.eu/ehealth-digital-health-and-care/european-health-data-space-regulation-ehds/european-health-data-space-ehds-webinar-series_en> | N |
| European Health Data Space (EHDS) Webinar 3: Implementation, Governance, and Responsibilities | European Commission - DG SANTE | International organisations | English | Scientific Association/Society | Seminar/Webinar | European Health Data Space (EHDS) Webinar Series | Online | 2025 | N | N | Y | N | Y | Y | N | N | Third EHDS webinar on EHDS implementation, governance and responsibilities. | <https://health.ec.europa.eu/ehealth-digital-health-and-care/european-health-data-space-regulation-ehds/european-health-data-space-ehds-webinar-series_en> | N |
| EU health security workshop: Methods and Tools for Evidence-Based Public Health (EBPH) | European Centre for Disease Prevention and Control (ECDC) | International organisations | English | Scientific Association/Society | Seminar/Webinar |  | In presence | 2025 | N | N | N | Y | N | N | N | Y | Four-day regional workshop in Stockholm on evidence-based public health methods, with key focus on AI use in evidence retrieval. | <https://www.ecdc.europa.eu/en/news-events/eu-health-security-workshop-strengthens-regional-skills-evidence-based-public-health> | N |
| Sessione parallela: Artificial Intelligence in Healthcare | European Health Management Association (EHMA) | International organisations | English | Scientific Association/Society | Conference session | EHMA 2025 Annual Conference | In presence | 2025 | Y | N | Y | N | N | Y | N | Y | Session on AI integration in hospital management, primary care, surgery, emergency and long-term care, including ethical-legal considerations and AI-based clinical decision support. | <https://ehmaconference.org/ehma-2025-conference-programme/> | N |
| Sessione parallela: Digital transformation in healthcare | European Health Management Association (EHMA) | International organisations | English | Scientific Association/Society | Conference session | EHMA 2025 Annual Conference | In presence | 2025 | Y | Y | Y | N | N | Y | N | Y | Session on how digital technologies reshape professional practices, organisational structures and patient experiences: AI, gamification in training, ethical complexities, technostress and professional role transformation. | <https://ehmaconference.org/ehma-2025-conference-programme/> | N |
| Sessione parallela: Data-driven healthcare - From data to insight and impact | European Health Management Association (EHMA) | International organisations | English | Scientific Association/Society | Conference session | EHMA 2025 Annual Conference | In presence | 2025 | Y | N | Y | Y | Y | N | N | N | Session on data-driven approaches to optimise healthcare: data acquisition, processing and integration. | <https://ehmaconference.org/ehma-2025-conference-programme/> | N |
| Sessione parallela: Digital solutions reshaping healthcare delivery | European Health Management Association (EHMA) | International organisations | English | Scientific Association/Society | Conference session | EHMA 2025 Annual Conference | In presence | 2025 | Y | N | Y | N | N | N | N | N | Session on digital solutions reshaping healthcare delivery: telemonitoring systems, digital transformation policies, telehealth integration, telemedicine and XR-telerehabilitation. | <https://ehmaconference.org/ehma-2025-conference-programme/> | N |
| Sessione parallela: Digital transformation and healthcare innovation | European Health Management Association (EHMA) | International organisations | English | Scientific Association/Society | Conference session | EHMA 2025 Annual Conference | In presence | 2025 | Y | N | Y | N | Y | Y | N | Y | Session on digital innovation impacts through case studies: EHR, AI applications, digital skills, value co-creation, regulatory compliance and rehabilitation platforms. | <https://ehmaconference.org/ehma-2025-conference-programme/> | N |
| Workshop: BeWell Blueprint - Digital and Green Skills Strategy | European Health Management Association (EHMA) | International organisations | English | Scientific Association/Society | Conference session | EHMA 2025 Annual Conference | In presence | 2025 | N | Y | Y | N | N | N | N | N | Workshop with WHO Regional Office for Europe on health workforce competencies, presenting BeWell initiative evidence: competency matrices, curricula and training programmes for digital and green skills. | <https://ehmaconference.org/ehma-2025-conference-programme/> | N |
| Keynote: Transforming Public Health Professions in the Digital Age | EuroNet MRPH | International organisations | English | Scientific Association/Society | Conference session | EuroNet MRPH Spring Meeting 2025 | In presence | 2025 | N | Y | Y | N | N | N | N | N | Keynote on transforming public health professions in the digital age. | <https://euronetmrph.org/wp-content/uploads/2025/05/Program-SM25-Paris.pdf> | N |
| Workshop: Building a Knowledge Foundation | EuroNet MRPH | International organisations | English | Scientific Association/Society | Conference session | EuroNet MRPH Spring Meeting 2025 | In presence | 2025 | Y | Y | Y | Y | Y | N | N | N | Workshop on building a knowledge foundation for digital transformation. | <https://euronetmrph.org/wp-content/uploads/2025/05/Program-SM25-Paris.pdf> | N |
| Roundtable: Is it ethical not to use new technologies in healthcare? | EuroNet MRPH | International organisations | English | Scientific Association/Society | Conference session | EuroNet MRPH Spring Meeting 2025 | In presence | 2025 | N | N | N | N | N | Y | N | N | Roundtable on the ethics of not using new technologies in healthcare. | <https://euronetmrph.org/wp-content/uploads/2025/05/Program-SM25-Paris.pdf> | N |
| Roundtable: Change and Innovation Management | EuroNet MRPH | International organisations | English | Scientific Association/Society | Conference session | EuroNet MRPH Spring Meeting 2025 | In presence | 2025 | N | N | Y | N | N | N | N | N | Roundtable on change and innovation management. | <https://euronetmrph.org/wp-content/uploads/2025/05/Program-SM25-Paris.pdf> | N |
| Roundtable: Toward a prevention-centered European health system - innovate under constraints | EuroNet MRPH | International organisations | English | Scientific Association/Society | Conference session | EuroNet MRPH Spring Meeting 2025 | In presence | 2025 | Y | Y | Y | N | N | N | N | N | Roundtable on innovating under constraints toward a prevention-centred European health system. | <https://euronetmrph.org/wp-content/uploads/2025/05/Program-SM25-Paris.pdf> | N |
| Workshop: Building the Roadmap for Transformation | EuroNet MRPH | International organisations | English | Scientific Association/Society | Conference session | EuroNet MRPH Spring Meeting 2025 | In presence | 2025 | N | Y | Y | N | N | N | N | N | Workshop on building the roadmap for digital transformation. | <https://euronetmrph.org/wp-content/uploads/2025/05/Program-SM25-Paris.pdf> | N |
| Webinar: Closing the digital skills gap for the European health and care workforce | European Observatory on Health Systems and Policies; European Health Management Association (EHMA) | International organisations | English | Scientific Association/Society | Seminar/Webinar |  | Online | 2025 | N | Y | Y | N | N | N | N | N | High-level webinar on upskilling the European healthcare workforce for digital transformation, with launch of Observatory policy brief. | <https://www.youtube.com/watch?v=K-23OqBIOHE> | N |
| Workshop: REBECCA 360 Platform - Real-World Data for breast cancer care | REBECCA Project Consortium | International organisations | English | Scientific Association/Society | Conference session | EHMA 2025 Annual Conference | In presence | 2025 | Y | N | Y | Y | Y | Y | N | N | World Café workshop on how real-world data improves post-treatment breast cancer care, discussing clinical workflow integration from perspectives of decision-makers, health authorities, tech companies and patient organisations. | <https://ehmaconference.org/ehma-2025-conference-programme/> | N |
| Understanding and addressing digital determinants of health | WHO Regional Office for Europe | International organisations | English | Scientific Association/Society | Seminar/Webinar |  | Online | 2025 | N | Y | N | N | N | N | N | N | Webinar on understanding and addressing digital determinants of health. | <https://www.who.int/europe/news-room/events/item/2025/01/29/default-calendar/understanding-and-addressing-digital-determinants-of-health> | N |
| Electronic health records in the age of AI | WHO Regional Office for Europe | International organisations | English | Scientific Association/Society | Seminar/Webinar | Decoding Data and Digital Health Webinar Series | Online | 2025 | N | N | N | Y | Y | Y | N | Y | Webinar on AI's role in electronic health records and how AI is redefining their future. | <https://www.who.int/europe/news-room/events/item/2025/10/08/default-calendar/electronic-health-records-in-the-age-of-ai> | N |
| Health data governance in the age of AI | WHO Regional Office for Europe | International organisations | English | Scientific Association/Society | Seminar/Webinar | Decoding Data and Digital Health Webinar Series | Online | 2025 | N | N | N | N | Y | Y | N | Y | Session on health data governance in the AI era, based on WHO/Europe policy brief. | <https://www.youtube.com/watch?v=RmSZTZTY4mw> | N |
| Strengthening telemedicine: sustainable practices and policy pathways for health systems | WHO Regional Office for Europe | International organisations | English | Scientific Association/Society | Seminar/Webinar | Decoding Data and Digital Health Webinar Series | Online | 2025 | Y | N | Y | N | N | N | N | N | Webinar (WHO/Europe and OECD) on telemedicine frameworks, tools and best practices for sustainable implementation. | <https://www.youtube.com/watch?v=58FLI1ugnt4> | N |
| Understanding and addressing digital determinants of health | WHO Regional Office for Europe | International organisations | English | Scientific Association/Society | Seminar/Webinar | Decoding Data and Digital Health Webinar Series | Online | 2025 | N | Y | N | N | N | N | N | N | Session on digital determinants of health (internet access, digital literacy, device availability) and health equity implications. | <https://eurohealthnet.eu/publication/who-webinar-on-understanding-and-addressing-digital-determinants-of-health/> | N |
| Mastère Spécialisé® Ingénierie et management des technologies de santé (IMTS) | EHESP School of Public Health | France | French | School/University | Training course |  | In presence | 2020 | Y | N | Y | Y | Y | Y | N | Y | Postgraduate degree (Mastère Spécialisé®) on health technology engineering and management. 'Numérique en santé' module covers IT, health data management, AI, modelling, simulation and cybersecurity. Also includes biomedical technologies, procurement and logistics. | <https://www.ehesp.fr/formation/formations-diplomantes/mastere-specialise-ingenierie-et-management-des-technologies-de-sante/> | N |
| Mastère Spécialisé® Ingénierie et management des technologies de santé (IMTS) | EHESP School of Public Health | France | French | School/University | Training course |  | In presence | 2021 | Y | N | Y | Y | Y | Y | N | Y | Postgraduate degree (Mastère Spécialisé®) on health technology engineering and management. 'Numérique en santé' module covers IT, health data management, AI, modelling, simulation and cybersecurity. Also includes biomedical technologies, procurement and logistics. | <https://www.ehesp.fr/formation/formations-diplomantes/mastere-specialise-ingenierie-et-management-des-technologies-de-sante/> | N |
| Harnessing Natural Language Processing on Biomedical Big Data for Pharmacovigilance | Isped - School of Public Health - Université de Bordeaux | France | French | School/University | Seminar/Webinar | Séminaires Isped 2021 | Online | 2021 | Y | N | N | Y | N | N | N | Y | Seminar on natural language processing applied to biomedical big data for pharmacovigilance. | <https://www.isped.u-bordeaux.fr/Isped/S%C3%A9minaires/S%C3%A9minaires-2021> | N |
| Sessioni su "Expériences de la télémédecine" | Société Française de Santé Publique (SFSP) | France | French | Scientific Association/Society | Conference session | Congrès SFSP 2021 | In presence | 2021 | Y | N | Y | N | N | N | N | N | Congress sessions on telemedicine experiences, focusing on e-therapeutic patient education. | <https://www.congres.sfsp.fr/presentations-2021/> | N |
| Mastère Spécialisé® Ingénierie et management des technologies de santé (IMTS) | EHESP School of Public Health | France | French | School/University | Degree programme |  | In presence | 2022 | Y | N | Y | Y | Y | Y | N | Y | Postgraduate degree (Mastère Spécialisé®) on health technology engineering and management. 'Numérique en santé' module covers IT, health data management, AI, modelling, simulation and cybersecurity. Also includes biomedical technologies, procurement and logistics. | <https://www.ehesp.fr/formation/formations-diplomantes/mastere-specialise-ingenierie-et-management-des-technologies-de-sante/> | N |
| Covid-19 : la télémédecine en mutation ? | EHESP School of Public Health | France | French | School/University | Seminar/Webinar | Webinaires du mardi | Online | 2022 | Y | N | Y | N | N | N | N | N | Seminar on telemedicine transformation during COVID-19. | <https://www.ehesp.fr/campus/webinaires-du-mardi/archives-seminaires-du-mardi/> | N |
| Mastère Spécialisé® Ingénierie et management des technologies de santé (IMTS) | EHESP School of Public Health | France | French | School/University | Training course |  | In presence | 2023 | Y | N | Y | Y | Y | Y | N | Y | Postgraduate degree (Mastère Spécialisé®) on health technology engineering and management. 'Numérique en santé' module covers IT, health data management, AI, modelling, simulation and cybersecurity. Also includes biomedical technologies, procurement and logistics. | <https://www.ehesp.fr/formation/formations-diplomantes/mastere-specialise-ingenierie-et-management-des-technologies-de-sante/> | N |
| Le potentiel de l'IA en établissement de santé | EHESP School of Public Health | France | French | School/University | Seminar/Webinar | DINUSA - Programme national (France 2030) | Online | 2023 | N | N | Y | N | N | Y | N | Y | Continuing education on AI potential in healthcare facilities. Part of DINUSA national programme (France 2030). | <https://www.ehesp.fr/formation/formations-en-ligne/plateforme-dinusa-numerique-en-sante/> | N |
| Cybersécurité en santé | EHESP School of Public Health | France | French | School/University | Seminar/Webinar | DINUSA - Programme national (France 2030) | Online | 2023 | N | N | Y | N | Y | Y | N | N | Continuing education on cybersecurity in healthcare: data protection and cyber risk management. Part of DINUSA programme. | <https://www.ehesp.fr/formation/formations-en-ligne/plateforme-dinusa-numerique-en-sante/> | N |
| La télémédecine, un outil d'équité d'accès aux soins et services de santé en Afrique subsaharienne | Isped - School of Public Health - Université de Bordeaux | France | French | School/University | Seminar/Webinar | Séminaires Isped 2023 | Online | 2023 | Y | Y | Y | N | N | N | N | N | Seminar on telemedicine as a tool for equitable access to healthcare in sub-Saharan Africa. | <https://www.isped.u-bordeaux.fr/Isped/S%C3%A9minaires/S%C3%A9minaires-2023> | N |
| Congrès SFSP 2023 - Thematic Session 36: Numérique : leviers et inégalités | Société Française de Santé Publique (SFSP) | France | French | Scientific Association/Society | Conference session | Congrès SFSP 2023 | In presence | 2023 | N | Y | N | N | N | Y | N | N | Annual SFSP congress with thematic session 'Digital: levers and inequalities' on the dual role of digital technologies in improving health outcomes and creating inequalities. | <https://www.sfsp.fr/content-page/item/63195-congres-sfsp-2023> | N |
| Mastère Spécialisé® Ingénierie et management des technologies de santé (IMTS) | EHESP School of Public Health | France | French | School/University | Training course |  | In presence | 2024 | Y | N | Y | Y | Y | Y | N | Y | Postgraduate degree (Mastère Spécialisé®) on health technology engineering and management. 'Numérique en santé' module covers IT, health data management, AI, modelling, simulation and cybersecurity. Also includes biomedical technologies, procurement and logistics. | <https://www.ehesp.fr/formation/formations-diplomantes/mastere-specialise-ingenierie-et-management-des-technologies-de-sante/> | N |
| Le potentiel de l'IA en établissement de santé | EHESP School of Public Health | France | French | School/University | Seminar/Webinar | DINUSA - Programme national (France 2030) | Online | 2024 | N | N | Y | N | N | Y | N | Y | Continuing education on AI potential in healthcare facilities. Part of DINUSA national programme (France 2030). | <https://www.ehesp.fr/formation/formations-en-ligne/plateforme-dinusa-numerique-en-sante/> | N |
| Cybersécurité en santé | EHESP School of Public Health | France | French | School/University | Seminar/Webinar | DINUSA - Programme national (France 2030) | Online | 2024 | N | N | Y | N | Y | Y | N | N | Continuing education on cybersecurity in healthcare: data protection and cyber risk management. Part of DINUSA programme. | <https://www.ehesp.fr/formation/formations-en-ligne/plateforme-dinusa-numerique-en-sante/> | N |
| Santé digitale : des politiques de santé européanisée | EHESP School of Public Health | France | French | School/University | Seminar/Webinar | Webinaires du mardi | Online | 2024 | N | N | Y | N | N | Y | N | N | Seminar on European digital health policies. | <https://www.ehesp.fr/campus/webinaires-du-mardi/archives-seminaires-du-mardi/> | N |
| Mastère Spécialisé® Ingénierie et management des technologies de santé (IMTS) | EHESP School of Public Health | France | French | School/University | Training course |  | In presence | 2025 | Y | N | Y | Y | Y | Y | N | Y | Postgraduate degree (Mastère Spécialisé®) on health technology engineering and management. 'Numérique en santé' module covers IT, health data management, AI, modelling, simulation and cybersecurity. Also includes biomedical technologies, procurement and logistics. | <https://www.ehesp.fr/formation/formations-diplomantes/mastere-specialise-ingenierie-et-management-des-technologies-de-sante/> | N |
| Le potentiel de l'IA en établissement de santé | EHESP School of Public Health | France | French | School/University | Seminar/Webinar | DINUSA - Programme national (France 2030) | Online | 2025 | N | N | N | N | Y | Y | N | Y | Continuing education on AI potential in healthcare facilities. Part of DINUSA national programme (France 2030). | <https://www.ehesp.fr/formation/formations-en-ligne/plateforme-dinusa-numerique-en-sante/> | N |
| Cybersécurité en santé | EHESP School of Public Health | France | French | School/University | Seminar/Webinar | DINUSA - Programme national (France 2030) | Online | 2025 | Y | N | Y | N | Y | Y | N | N | Continuing education on cybersecurity in healthcare: data protection and cyber risk management. Part of DINUSA programme. | <https://www.ehesp.fr/formation/formations-en-ligne/plateforme-dinusa-numerique-en-sante/> | N |
| Applied Medical Informatics | Berlin School of Public Health (BSPH) - Charité | Germany | English | School/University | Training course | MSc Epidemiology | In presence | 2020 | Y | N | N | Y | Y | Y | N | N | Elective course within the Master in Epidemiology on practical application of medical informatics to epidemiological research and public health. | <https://www.moritzqueisner.de/events/> | N |
| Didattica digitale – Potenzialità, sfide e prospettive (in sanità pubblica) | Deutsche Gesellschaft für Public Health (DGPH) | Germany | German | Scientific Association/Society | Conference session | DGPH Jahrestagung 2020 | Online | 2020 | Y | Y | Y | N | N | N | N | N | Session on digital education: potential, challenges and perspectives for public health. | <https://www.dgph.info/fileadmin/user_upload/PDF/Newsletter/DGPH-Newsletter_Oktober_2021.pdf> | N |
| Applied Medical Informatics | Berlin School of Public Health (BSPH) - Charité | Germany | English | School/University | Training course | MSc Epidemiology | In presence | 2021 | Y | N | N | Y | Y | Y | N | N | Elective course within the Master in Epidemiology on practical application of medical informatics to epidemiological research and public health. | <https://www.moritzqueisner.de/events/> | N |
| Partecipazione e divisione digitale - Sfide della prevenzione e della cura nella trasformazione digitale | Deutsche Gesellschaft für Public Health (DGPH) | Germany | German | Scientific Association/Society | Conference session | DGPH Jahrestagung 2021 | Online | 2021 | N | Y | Y | N | N | Y | N | N | Session on participation and digital divide as challenges for prevention and care in digital transformation. | <https://www.dgph.info/fileadmin/user_upload/PDF/Newsletter/DGPH-Newsletter_Maerz_2021.pdf> | N |
| Introduction to Medical Informatics | Berlin School of Public Health (BSPH) - Charité | Germany | English | School/University | Training course |  | Online | 2022 | Y | N | N | Y | Y | Y | N | N | Intensive course on medical informatics: information systems, standards, data processing, security, privacy, software development, open science in medicine. | <https://bsph.charite.de/studienangebot/intensive_short_courses/archiv> | N |
| Introduction to Medical Informatics | Berlin School of Public Health (BSPH) - Charité | Germany | English | School/University | Training course |  | Online | 2022 | Y | N | N | Y | Y | Y | N | N | Five-day intensive course on medical informatics fundamentals: health information systems and standards, data processing and integration, information security and data protection. | <https://bsph.charite.de/en/academic_programs/intensive_short_courses/> | N |
| Applied Medical Informatics | Berlin School of Public Health (BSPH) - Charité | Germany | English | School/University | Training course | MSc Epidemiology | In presence | 2022 | Y | N | N | Y | Y | Y | N | N | Elective course within the Master in Epidemiology on practical application of medical informatics to epidemiological research and public health. | <https://www.moritzqueisner.de/events/> | N |
| 4th RUHR School of Modern Epidemiology - introduction to machine learning and causal inference | Universitätsklinikum Essen (AöR) | Germany | English | School/University | Training course | RUHR School of Modern Epidemiology | In presence | 2022 | Y | N | N | Y | N | N | N | Y | Introductory course on machine learning and causal inference for epidemiologists. | <https://imibe.uk-essen.de/wp-content/uploads/2022/03/Flyer4thRuhrSchool_2022_ain.pdf> | N |
| Introduction to Medical Informatics | Berlin School of Public Health (BSPH) - Charité | Germany | English | School/University | Training course |  | Online | 2022 | Y | N | N | Y | Y | Y | N | N | Intensive course on medical informatics: information systems, standards, data processing, security, privacy, software development, open science in medicine. | <https://bsph.charite.de/studienangebot/intensive_short_courses/archiv> | N |
| Introduction to Medical Informatics | Berlin School of Public Health (BSPH) - Charité | Germany | English | School/University | Training course |  | Online | 2023 | Y | N | N | Y | Y | Y | N | N | Intensive course on medical informatics: information systems, standards, data processing, security, privacy, software development, open science in medicine. | <https://bsph.charite.de/studienangebot/intensive_short_courses/archiv> | N |
| Introduction to Medical Informatics | Berlin School of Public Health (BSPH) - Charité | Germany | English | School/University | Training course |  | Online | 2025 | Y | N | N | Y | Y | Y | N | N | Intensive course on medical informatics: information systems, standards, data processing, security, privacy, software development, open science in medicine. | <https://bsph.charite.de/studienangebot/intensive_short_courses/archiv> | N |
| Applied Medical Informatics | Berlin School of Public Health (BSPH) - Charité | Germany | English | School/University | Training course | MSc Epidemiology | In presence | 2023 | Y | N | N | Y | Y | Y | N | N | Elective course within the Master in Epidemiology on practical application of medical informatics to epidemiological research and public health. | <https://www.moritzqueisner.de/events/> | N |
| Bedarfsorientierte Unterstützung und Qualifizierung für digitale Transformationsprozesse im Öffentlichen Gesundheitsdienst (BUDDI) | Bundesverband der Ärztinnen und Ärzte des Öffentlichen Gesundheitsdienstes (BVÖGD) | Germany | German | Scientific Association/Society | Conference session | 72. Wissenschaftlicher Kongress BVÖGD 2023 | In presence | 2023 | Y | N | Y | N | N | N | N | N | Presentation of the BUDDI project on needs-based support and qualification for digital transformation in German public health services. | <https://www.congress-compact.de/pdf/2023-04-26-29-BVOEGD_Kongress-Programm.pdf> | N |
| Der Pakt für den Öffentlichen Gesundheitsdienst und seine Umsetzung - AG Digitalisierung | Bundesverband der Ärztinnen und Ärzte des Öffentlichen Gesundheitsdienstes (BVÖGD); Bundesministerium für Gesundheit (BMG) | Germany | German | Scientific Association/Society | Conference session | 72. Wissenschaftlicher Kongress BVÖGD 2023 | In presence | 2023 | Y | N | Y | N | N | N | N | N | Lunch symposium on the Pact for German Public Health Service and its implementation, including the Digitalisation Working Group. | <https://www.congress-compact.de/pdf/2023-04-26-29-BVOEGD_Kongress-Programm.pdf> | N |
| International Germany Alumni Seminar: Digital Transformation in Global Health + DMEA 2023 | Heidelberg Institute of Global Health (HIGH) / DAAD | Germany | English | School/University | Seminar/Webinar |  | Hybrid | 2023 | Y | Y | Y | N | N | N | N | N | Seminar for DAAD alumni from LMIC countries on digital transformation in global health, followed by DMEA fair participation. | <https://www.klinikum.uni-heidelberg.de/fileadmin/inst_hygiene/tropenhygiene/Teaching/Alumni/Call_for_applications_DMEA_v4.pdf> | N |
| Designing, evaluating and implementing digital public health interventions | Leibniz ScienceCampus Digital Public Health (LSC DiPH) | Germany | English | School/University | Training course |  | In presence | 2023 | Y | N | Y | N | N | N | N | N | Five-day summer school on designing, evaluating and implementing digital public health interventions. | <https://www.lsc-digital-public-health.de/en/about-ecra/summer-school.html> | N |
| AI-Supported Decision Making in Public Health | Robert Koch Institute (RKI); Zentrum für Künstliche Intelligenz in der Public Health-Forschung (ZKI-PH) | Germany | English | Scientific Association/Society | Conference session | ZKI-PH Symposium 2023 | Hybrid | 2023 | Y | N | Y | Y | N | N | N | N | Session on AI-supported decision-making in public health. | <https://www.rki.de/EN/Institute/Organisation/Departments/ZKI-PH/Events/AIinPH-Symposium.html> | N |
| AI-Supported Diagnostic and Imaging Tools | Robert Koch Institute (RKI); Zentrum für Künstliche Intelligenz in der Public Health-Forschung (ZKI-PH) | Germany | English | Scientific Association/Society | Conference session | ZKI-PH Symposium 2023 | Hybrid | 2023 | Y | N | N | N | N | N | N | N | Session on AI-supported diagnostic and imaging tools. | <https://www.rki.de/EN/Institute/Organisation/Departments/ZKI-PH/Events/AIinPH-Symposium.html> | N |
| Technology Showcase | Robert Koch Institute (RKI); Zentrum für Künstliche Intelligenz in der Public Health-Forschung (ZKI-PH) | Germany | English | Scientific Association/Society | Conference session | ZKI-PH Symposium 2023 | Hybrid | 2023 | Y | N | N | N | N | N | N | N | Showcase of innovative AI technologies for public health. | <https://www.rki.de/EN/Institute/Organisation/Departments/ZKI-PH/Events/AIinPH-Symposium.html> | N |
| Privacy Preserving AI Tools in Public Health Research | Robert Koch Institute (RKI); Zentrum für Künstliche Intelligenz in der Public Health-Forschung (ZKI-PH) | Germany | English | Scientific Association/Society | Conference session | ZKI-PH Symposium 2023 | Hybrid | 2023 | N | N | N | N | Y | Y | N | Y | Session on privacy-preserving AI tools in public health research. | <https://www.rki.de/EN/Institute/Organisation/Departments/ZKI-PH/Events/AIinPH-Symposium.html> | N |
| Das Frankfurter Gesundheitsdashboard zur integrierten Gesundheitsberichterstattung | Bundesverband der Ärztinnen und Ärzte des Öffentlichen Gesundheitsdienstes (BVÖGD) | Germany | German | Scientific Association/Society | Conference session | 73. Wissenschaftlicher Kongress BVÖGD 2024 | In presence | 2024 | Y | N | N | Y | Y | N | N | N | Presentation on Frankfurt's digital health dashboard for integrated health reporting. | <https://www.congress-compact.de/pdf/2024-04-24-27-BVOEGD_Kongress-Programm.pdf> | N |
| Ein interaktives Dashboard Kindergesundheit zur städtischen Ressourcensteuerung in Leipzig | Bundesverband der Ärztinnen und Ärzte des Öffentlichen Gesundheitsdienstes (BVÖGD) | Germany | German | Scientific Association/Society | Conference session | 73. Wissenschaftlicher Kongress BVÖGD 2024 | In presence | 2024 | Y | N | N | Y | Y | N | N | N | Presentation on an interactive child health dashboard for urban resource management in Leipzig. | <https://www.congress-compact.de/pdf/2024-04-24-27-BVOEGD_Kongress-Programm.pdf> | N |
| Was hätte eine an der Ebola-Epidemie trainierte KI für unser Verhalten in der Corona-Pandemie vorhergesagt? | Bundesverband der Ärztinnen und Ärzte des Öffentlichen Gesundheitsdienstes (BVÖGD) | Germany | German | Scientific Association/Society | Conference session | 73. Wissenschaftlicher Kongress BVÖGD 2024 | In presence | 2024 | N | N | N | Y | N | N | N | Y | Presentation on what AI trained on Ebola epidemic would have predicted for COVID-19 pandemic behaviour. | <https://www.congress-compact.de/pdf/2024-04-24-27-BVOEGD_Kongress-Programm.pdf> | N |
| Digitalisierung im ÖGD - Block 1: Digitale Kompetenzen und Infrastruktur | Bundesverband der Ärztinnen und Ärzte des Öffentlichen Gesundheitsdienstes (BVÖGD); Deutsche Gesellschaft für Öffentliche Gesundheit (DGÖG) | Germany | German | Scientific Association/Society | Conference session | 73. Wissenschaftlicher Kongress BVÖGD 2024 | In presence | 2024 | Y | Y | Y | N | N | N | N | N | Session on digitalisation in German public health services: digital competency needs (BUDDI project), interoperable interfaces, telematics infrastructure connection, digitalisation in Bavaria. | <https://www.congress-compact.de/pdf/2024-04-24-27-BVOEGD_Kongress-Programm.pdf> | N |
| Digitalisierung im ÖGD - Block 2: Aspetti pratici della digitalizzazione | Bundesverband der Ärztinnen und Ärzte des Öffentlichen Gesundheitsdienstes (BVÖGD); Deutsche Gesellschaft für Öffentliche Gesundheit (DGÖG) | Germany | German | Scientific Association/Society | Conference session | 73. Wissenschaftlicher Kongress BVÖGD 2024 | In presence | 2024 | Y | Y | Y | Y | N | N | Y | N | Session on practical digitalisation in health offices: digitalisation in Magdeburg, digital competency strengthening in Jena, digital parent communication via Instagram, HuGO online health panel in Lower Saxony. | <https://www.congress-compact.de/pdf/2024-04-24-27-BVOEGD_Kongress-Programm.pdf> | N |
| Digitalisierung im ÖGD - Block 3: DEMIS e competenze digitali | Bundesverband der Ärztinnen und Ärzte des Öffentlichen Gesundheitsdienstes (BVÖGD); Deutsche Gesellschaft für Öffentliche Gesundheit (DGÖG) | Germany | German | Scientific Association/Society | Conference session | 73. Wissenschaftlicher Kongress BVÖGD 2024 | In presence | 2024 | Y | N | Y | Y | Y | N | N | N | Session on digital competency assessment of health office staff, DEMIS system evaluation, Baden-Württemberg digitalisation progress, and DEMIS development roadmap. | <https://www.congress-compact.de/pdf/2024-04-24-27-BVOEGD_Kongress-Programm.pdf> | N |
| Debatte: KI in der Versorgungsforschung | Deutscher Kongress für Versorgungsforschung (DKVF); Deutsches Netzwerk Versorgungsforschung (DNVF) | Germany | German | Scientific Association/Society | Conference session | DKVF 2024 | In presence | 2024 | Y | N | N | Y | N | N | N | Y | Debate on AI use and implications in health services research. | <https://www.dkvf.de/de/newsletter-archive/dkvf-programm-jetzt-online.html> | N |
| Sessione V01: Nutzung von KI in der Versorgungsforschung | Deutscher Kongress für Versorgungsforschung (DKVF); Deutsches Netzwerk Versorgungsforschung (DNVF) | Germany | German | Scientific Association/Society | Conference session | DKVF 2024 | In presence | 2024 | Y | N | N | Y | N | N | N | Y | Session on AI applications in health services research. | <https://www.egms.de/dynamic/en/meetings/dkvf2024/index.htm> | N |
| Sessione V41: Digitale Interventionen: Entwicklung und Nutzung von DiGAs | Deutscher Kongress für Versorgungsforschung (DKVF); Deutsches Netzwerk Versorgungsforschung (DNVF) | Germany | German | Scientific Association/Society | Conference session | DKVF 2024 | In presence | 2024 | Y | N | Y | N | N | Y | N | N | Session on development and use of Digital Health Applications (DiGA). | <https://www.egms.de/dynamic/en/meetings/dkvf2024/index.htm> | N |
| Sessione S1: Digitale Innovationen in der Gesundheitsversorgung | Deutsche Gesellschaft für Sozialmedizin und Prävention (DGSMP); Deutsche Gesellschaft für Epidemiologie (DGEpi); Deutsche Gesellschaft für Medizinische Soziologie (DGMS); Deutsche Gesellschaft für Public Health (DGPH) | Germany | German | Scientific Association/Society | Conference session | DGSMP/DGEpi/DGMS/DGPH Tagung 2024 | In presence | 2024 | Y | N | Y | N | N | N | N | N | Session on digital innovations in healthcare. | <https://gesundheit-gemeinsam.de/wp-content/uploads/2024/09/Programm_Kooperationstagung_Freitag_13.09.2024.pdf> | N |
| Sessione S45: Digitale Technologien in der Gesundheitsversorgung | Deutsche Gesellschaft für Sozialmedizin und Prävention (DGSMP); Deutsche Gesellschaft für Epidemiologie (DGEpi); Deutsche Gesellschaft für Medizinische Soziologie (DGMS); Deutsche Gesellschaft für Public Health (DGPH) | Germany | German | Scientific Association/Society | Conference session | DGSMP/DGEpi/DGMS/DGPH Tagung 2024 | In presence | 2024 | Y | Y | Y | N | N | Y | N | N | Session on digital technologies for equity and participation in healthcare. | <https://gesundheit-gemeinsam.de/wp-content/uploads/2024/09/Programm_Kooperationstagung_Freitag_13.09.2024.pdf> | N |
| Sessione V13: Digital Health Literacy und digitale Technologien in der Pflege | Deutsche Gesellschaft für Sozialmedizin und Prävention (DGSMP); Deutsche Gesellschaft für Epidemiologie (DGEpi); Deutsche Gesellschaft für Medizinische Soziologie (DGMS); Deutsche Gesellschaft für Public Health (DGPH) | Germany | German | Scientific Association/Society | Conference session | DGSMP/DGEpi/DGMS/DGPH Tagung 2024 | In presence | 2024 | Y | Y | N | N | N | N | N | N | Session on digital health literacy and digital technologies in nursing care. | <https://gesundheit-gemeinsam.de/wp-content/uploads/2024/09/Programm_Kooperationstagung_Mittwoch_11.09.2024-2.pdf> | N |
| Sessione V29: Digitale Interventionen | Deutsche Gesellschaft für Sozialmedizin und Prävention (DGSMP); Deutsche Gesellschaft für Epidemiologie (DGEpi); Deutsche Gesellschaft für Medizinische Soziologie (DGMS); Deutsche Gesellschaft für Public Health (DGPH) | Germany | German | Scientific Association/Society | Conference session | DGSMP/DGEpi/DGMS/DGPH Tagung 2024 | In presence | 2024 | Y | N | N | N | N | N | N | N | Session on digital interventions in healthcare. | <https://gesundheit-gemeinsam.de/wp-content/uploads/2024/09/Programm_Kooperationstagung_Donnerstag_12.09.2024-2.pdf> | N |
| Sessione V54: Digital-Health-Tools I Digitalisierung im Gesundheitswesen | Deutsche Gesellschaft für Sozialmedizin und Prävention (DGSMP); Deutsche Gesellschaft für Epidemiologie (DGEpi); Deutsche Gesellschaft für Medizinische Soziologie (DGMS); Deutsche Gesellschaft für Public Health (DGPH) | Germany | German | Scientific Association/Society | Conference session | DGSMP/DGEpi/DGMS/DGPH Tagung 2024 | In presence | 2024 | Y | N | Y | N | N | N | N | N | Session on digital health tools and healthcare digitalisation. | <https://gesundheit-gemeinsam.de/wp-content/uploads/2024/09/Programm_Kooperationstagung_Donnerstag_12.09.2024-2.pdf> | N |
| Symposium S11: Wen erreichen wir mit digitaler prävention und Gesundheitsförderung? Und wie? | Deutsche Gesellschaft für Sozialmedizin und Prävention (DGSMP); Deutsche Gesellschaft für Epidemiologie (DGEpi); Deutsche Gesellschaft für Medizinische Soziologie (DGMS); Deutsche Gesellschaft für Public Health (DGPH) | Germany | German | Scientific Association/Society | Conference session | DGSMP/DGEpi/DGMS/DGPH Tagung 2024 | In presence | 2024 | N | Y | N | N | N | Y | N | N | Symposium on reach and methods of digital prevention and health promotion, with focus on equity. | <https://gesundheit-gemeinsam.de/wp-content/uploads/2024/09/Programm_Kooperationstagung_Donnerstag_12.09.2024-2.pdf> | N |
| Workshop W10: Digitale Gesundheitskompetenz | Deutsche Gesellschaft für Sozialmedizin und Prävention (DGSMP); Deutsche Gesellschaft für Epidemiologie (DGEpi); Deutsche Gesellschaft für Medizinische Soziologie (DGMS); Deutsche Gesellschaft für Public Health (DGPH) | Germany | German | Scientific Association/Society | Conference session | DGSMP/DGEpi/DGMS/DGPH Tagung 2024 | In presence | 2024 | N | Y | N | N | N | N | N | N | Workshop on digital health competence: definition, measurement and promotion. | <https://gesundheit-gemeinsam.de/wp-content/uploads/2024/09/Programm_Kooperationstagung_Mittwoch_11.09.2024-2.pdf> | N |
| Session V22: KI und maschinelles Lernen | Deutsche Gesellschaft für Sozialmedizin und Prävention (DGSMP); Deutsche Gesellschaft für Epidemiologie (DGEpi); Deutsche Gesellschaft für Medizinische Soziologie (DGMS); Deutsche Gesellschaft für Public Health (DGPH) | Germany | German | Scientific Association/Society | Conference session | DGSMP/DGEpi/DGMS/DGPH Tagung 2024 | In presence | 2024 | Y | N | N | Y | N | N | N | Y | Session on AI and machine learning at the joint annual conference of German societies for social medicine, epidemiology, medical sociology and public health. | <https://gesundheit-gemeinsam.de/wp-content/uploads/2024/09/Programm_Kooperationstagung_Donnerstag_12.09.2024-2.pdf> | N |
| Vortragssession (V1): Digitale Transformation | Deutsche Gesellschaft für Sozialmedizin und Prävention (DGSMP); Deutsche Gesellschaft für Epidemiologie (DGEpi); Deutsche Gesellschaft für Medizinische Soziologie (DGMS); Deutsche Gesellschaft für Public Health (DGPH) | Germany | German | Scientific Association/Society | Conference session | DGSMP/DGEpi/DGMS/DGPH Tagung 2024 | In presence | 2024 | Y | N | Y | N | N | N | N | N | Oral presentation session on digital transformation. | <https://gesundheit-gemeinsam.de/dgsmp-dgepi-dgms-und-dgph/programm-dgsmp-dgepi-dgms-dgph/> | N |
| Vortragssession (V2): Semantische Interoperabilität und Terminologien | Deutsche Gesellschaft für Sozialmedizin und Prävention (DGSMP); Deutsche Gesellschaft für Epidemiologie (DGEpi); Deutsche Gesellschaft für Medizinische Soziologie (DGMS); Deutsche Gesellschaft für Public Health (DGPH) | Germany | German | Scientific Association/Society | Conference session | DGSMP/DGEpi/DGMS/DGPH Tagung 2024 | In presence | 2024 | Y | N | Y | N | Y | N | N | N | Oral presentation session on semantic interoperability and terminologies in digital health systems. | <https://gesundheit-gemeinsam.de/dgsmp-dgepi-dgms-und-dgph/programm-dgsmp-dgepi-dgms-dgph/> | N |
| Künstliche Intelligenz im ÖGD – KI verstehen | Akademie für Öffentliches Gesundheitswesen (Akademie ÖGW) | Germany | German | Scientific Association/Society | Seminar/Webinar | DGSMP/DGEpi/DGMS/DGPH Tagung 2024 | Online | 2025 | Y | N | N | Y | N | N | N | Y | Overview of AI fundamentals and applications in German public health services (data analysis, communication). First session of a new series. | <https://www.akademie-oegw.de/fortbildung/details/d-3412025> | N |
| Medizinprodukte im Focus von Software, Apps und KI | Akademie für Öffentliches Gesundheitswesen (Akademie ÖGW) | Germany | German | Scientific Association/Society | Seminar/Webinar |  | Online | 2025 | Y | N | Y | Y | N | Y | N | Y | Two-day online seminar for public health surveillance staff on software, apps and AI as medical devices: medical device software, cybersecurity, DiGA, and AI's role. | <https://www.akademie-oegw.de/fortbildung/details/q-32025> | N |
| Introduction to Medical Informatics | Berlin School of Public Health (BSPH) - Charité | Germany | English | School/University | Training course |  | Online | 2025 | N | N | Y | Y | Y | Y | N | N | Five-day intensive course on medical informatics fundamentals: health information systems and standards, data processing and integration, information security and data protection. | <https://bsph.charite.de/en/academic_programs/intensive_short_courses/> | N |
| Intelligente Patientensteuerung | BMC Kongress für Managed Care | Germany | German | Scientific Association/Society | Conference session | BMC Kongress 2025 | In presence | 2025 | Y | N | Y | Y | Y | N | N | Y | Session on intelligent patient management for efficiency in future healthcare systems. | <https://bmckongress.de/> | N |
| Managed Care Lecture - Digitalizzazione e KI nel sistema sanitario | BMC Kongress für Managed Care | Germany | German | Scientific Association/Society | Conference session | BMC Kongress 2025 | In presence | 2025 | N | N | Y | N | N | Y | N | Y | Managed Care Lecture on digitalisation and AI in healthcare. | <https://bmckongress.de/> | N |
| Pflege vernetzt | BMC Kongress für Managed Care | Germany | German | Scientific Association/Society | Conference session | BMC Kongress 2025 | In presence | 2025 | Y | N | Y | N | N | N | N | N | Session on networked care with focus on digital innovations and cross-sector coordination. | <https://bmckongress.de/> | N |
| ePA + KI + Plattformen + digitale Identitäten | BMC Kongress für Managed Care | Germany | German | Scientific Association/Society | Conference session | BMC Kongress 2025 | In presence | 2025 | Y | N | Y | N | Y | Y | N | Y | Session on electronic patient records (ePA) integrated with AI, platforms and digital identities. | <https://bmckongress.de/> | N |
| From data to decision - Utilizzo dati per l'assistenza | BMC Kongress für Managed Care | Germany | German | Scientific Association/Society | Conference session | BMC Kongress 2025 | In presence | 2025 | N | N | Y | Y | Y | N | N | N | Session on using data for clinical and managerial decisions in healthcare. | <https://bmckongress.de/> | N |
| Blitzlichter - KI in der Versorgung | BMC Kongress für Managed Care | Germany | German | Scientific Association/Society | Conference session | BMC Kongress 2025 | In presence | 2025 | Y | N | N | Y | Y | N | N | Y | Lightning session on practical AI applications in healthcare: automated data extraction. | <https://bmckongress.de/> | N |
| Hindernislauf für digitale Innovationen | BMC Kongress für Managed Care | Germany | German | Scientific Association/Society | Conference session | BMC Kongress 2025 | In presence | 2025 | Y | N | Y | N | N | Y | N | Y | Session on obstacles and barriers for digital innovations in healthcare. | <https://bmckongress.de/> | N |
| Next Generation Healthcare - Gen Z rimodella il sistema sanitario | BMC Kongress für Managed Care | Germany | German | Scientific Association/Society | Conference session | BMC Kongress 2025 | In presence | 2025 | Y | Y | Y | N | N | N | N | N | Session on how Generation Z is transforming healthcare with new digital expectations. | <https://bmckongress.de/> | N |
| Einheitliche Fachanwendung der Gesundheitsämter | Bundesverband der Ärztinnen und Ärzte des Öffentlichen Gesundheitsdienstes (BVÖGD) | Germany | German | Scientific Association/Society | Conference session | 74. Wissenschaftlicher Kongress BVÖGD 2025 | In presence | 2025 | Y | N | Y | N | Y | N | N | N | Session on unified software for German health offices (GA-Lotse): automated data visualisation, digital inspection checklists, Baden-Württemberg software platform, digital citizen services in Bavaria, unified open-source ÖGD architecture. | <https://www.congress-compact.de/pdf/2025_04_01-04_BVOEGD_Kongress_Programm.pdf> | N |
| ÖGDnet/Pakt ÖGD | Bundesverband der Ärztinnen und Ärzte des Öffentlichen Gesundheitsdienstes (BVÖGD) | Germany | German | Scientific Association/Society | Conference session | 74. Wissenschaftlicher Kongress BVÖGD 2025 | In presence | 2025 | Y | N | Y | N | Y | N | N | N | Session on digital support for public health offices: Agora platform, EMIGA@ÖGDnet for infection protection, DEMIS development, ÖGD Connect coordination platform, Pakt ÖGD digitalisation funding analysis. | <https://www.congress-compact.de/pdf/2025_04_01-04_BVOEGD_Kongress_Programm.pdf> | N |
| Interoperabilität: Datenvereinheitlichung und -zusammenführung | Bundesverband der Ärztinnen und Ärzte des Öffentlichen Gesundheitsdienstes (BVÖGD) | Germany | German | Scientific Association/Society | Conference session | 74. Wissenschaftlicher Kongress BVÖGD 2025 | In presence | 2025 | Y | N | N | Y | Y | N | N | N | Session on data interoperability in public health: automated school entry examination data, state-level data system in Brandenburg, LGL database for Bavarian health regions, RKI surveillance interoperability, MUT-ATLAS mental health network, Germany Health Panel for BIPAM. | <https://www.congress-compact.de/pdf/2025_04_01-04_BVOEGD_Kongress_Programm.pdf> | N |
| Artificial Intelligence and Digitalization in Public Health: Opportunities, Limits, Vision | Deutsche Gesellschaft für Public Health (DGPH) | Germany | German, English | Scientific Association/Society | Conference session | DGPH Jahrestagung 2025 | In presence | 2025 | Y | N | Y | N | N | Y | N | Y | Annual DGPH conference on AI and digitalisation in public health: opportunities, limits, visions. | <https://www.dgph.info/en/news/dgph-annual-conference-2025/> | N |
| Keynote: Handlungsfähigkeit in der digitalen und planetaren Umbruchszeit | Deutsche Gesellschaft für Sozialmedizin und Prävention (DGSMP) | Germany | German | Scientific Association/Society | Conference session | DGSMP Jahrestagung 2025 | In presence | 2025 | N | Y | N | N | N | Y | N | N | Opening keynote examining the dual impact of planetary crisis and uncontrolled technological innovation on public health, contrasting collective solutions with the individualistic focus of 'digital health'. | <https://access.online-registry.net/dgsmp2025/download/sessionlist/overview.html> | N |
| Pre-Conference: Klimagesundheitsförderung | Deutsche Gesellschaft für Sozialmedizin und Prävention (DGSMP) | Germany | German | Scientific Association/Society | Conference session | DGSMP Jahrestagung 2025 | In presence | 2025 | Y | N | N | N | N | Y | N | N | Pre-conference on climate health promotion, including digital tools. | <https://www.dgsmp-kongress.de/online-programm/> | N |
| Symposium: Gesundheits(kompetenz)förderung bei Menschen mit intellektueller Beeinträchtigung | Deutsche Gesellschaft für Sozialmedizin und Prävention (DGSMP) | Germany | German | Scientific Association/Society | Conference session | DGSMP Jahrestagung 2025 | In presence | 2025 | N | Y | N | N | N | Y | N | N | Symposium on health literacy promotion for people with intellectual disabilities, relevant for digital inclusion. | <https://www.dgsmp-kongress.de/online-programm/> | N |
| Pre-Conference: Einfach, interaktiv, individuell: Digitale Gesundheitsinterventionen selbst entwickeln – mit CIAS (UMG) | Deutsche Gesellschaft für Sozialmedizin und Prävention (DGSMP); Universitätsmedizin Göttingen (UMG) | Germany | German | Scientific Association/Society | Conference session | DGSMP Jahrestagung 2025 | In presence | 2025 | Y | N | Y | N | N | N | N | N | Hands-on pre-conference workshop on creating digital health interventions without programming using the CIAS open-source platform. | <https://www.dgsmp-kongress.de/online-programm/> | N |
| DIGITALISIERUNG UND KI IN PUBLIC HEALTH: Chancen, Grenzen, Visionen | Deutsche Gesellschaft für Public Health (DGPH) | Germany | German | Scientific Association/Society | Conference session | DGPH Jahrestagung 2025 | In presence | 2025 | Y | Y | Y | Y | Y | Y | N | Y | Plenary session on digitalisation and AI in public health: opportunities, limits, visions. | <https://www.dgph.info/fileadmin/user_upload/PDF/Tagungen_Seminare/Programm_Jahrestagung_2025_aktualisiert.pdf> | N |
| Grundlagen der Umsetzung digitaler Public-Health Maßnahmen: eine globale Perspektive | Deutsche Gesellschaft für Public Health (DGPH) | Germany | German | Scientific Association/Society | Conference session | DGPH Jahrestagung 2025 | In presence | 2025 | Y | Y | Y | N | Y | N | N | N | Workshop on fundamentals of implementing digital public health measures from a global perspective. | <https://www.dgph.info/fileadmin/user_upload/PDF/Tagungen_Seminare/Programm_Jahrestagung_2025_aktualisiert.pdf> | N |
| Implementing Digital Public Health Content in Public Health Education | Deutsche Gesellschaft für Public Health (DGPH) | Germany | English | Scientific Association/Society | Conference session | DGPH Jahrestagung 2025 | In presence | 2025 | Y | Y | Y | N | N | N | N | N | Workshop on implementing digital public health content in public health education. | <https://www.dgph.info/fileadmin/user_upload/PDF/Tagungen_Seminare/Programm_Jahrestagung_2025_aktualisiert.pdf> | N |
| Mind the gap: Wie lässt sich eine digitale Spaltung bei digitalen Gesundheitsförderungs- und Präventionsmaßnahmen vermeiden? | Deutsche Gesellschaft für Public Health (DGPH) | Germany | German | Scientific Association/Society | Conference session | DGPH Jahrestagung 2025 | In presence | 2025 | Y | Y | N | N | N | Y | N | N | Workshop on avoiding digital divide in digital health promotion and prevention measures. | <https://www.dgph.info/fileadmin/user_upload/PDF/Tagungen_Seminare/Programm_Jahrestagung_2025_aktualisiert.pdf> | N |
| Chancen und Potenziale von Digital Public Health für die Förderung betrieblicher Gesundheitskompetenz | Deutsche Gesellschaft für Public Health (DGPH) | Germany | German | Scientific Association/Society | Conference session | DGPH Jahrestagung 2025 | In presence | 2025 | Y | Y | Y | N | N | N | N | N | Workshop on digital public health potential for promoting workplace health literacy. | <https://www.dgph.info/fileadmin/user_upload/PDF/Tagungen_Seminare/Programm_Jahrestagung_2025_aktualisiert.pdf> | N |
| Four micro-credentials for cross-professional digital skills development | Ostfalia University of Applied Sciences; DDS-MAP | Germany | German, English | School/University | Conference session | Future of Care Cluster Conference | In presence | 2025 | Y | Y | Y | N | N | N | N | N | Interactive session on four training modules for cross-professional digital skills development in healthcare. | <https://ddsmap.easpd.eu/2025/03/18/dds-map-at-the-heath-summit-conference-in-germany/> | N |
| Thinking care digitally: DDS-MAP presentation at Health Summit Weimar | Ostfalia University of Applied Sciences; DDS-MAP | Germany | German, English | School/University | Conference session | Health Summit Weimar | In presence | 2025 | Y | Y | Y | N | Y | N | N | N | Presentation on standardised care language as foundation for digitalisation, showcasing DDS-MAP modules and VR environment. | <https://ddsmap.easpd.eu/2025/03/18/dds-map-at-the-heath-summit-conference-in-germany/> | N |
| AI-Supported Decision-Making in Public Health | Robert Koch Institute (RKI); Zentrum für Künstliche Intelligenz in der Public Health-Forschung (ZKI-PH) | Germany | English | Scientific Association/Society | Conference session | ZKI-PH Symposium 2025 | In presence | 2025 | Y | N | Y | Y | Y | N | N | Y | Session on AI-supported decision-making in public health with focus on early detection of disease patterns. | <https://www.rki.de/EN/Institute/Organisation/Departments/ZKI-PH/Events/AIinPH-Symposium.html> | N |
| AI-based Strategies to Overcome AMR Challenges | Robert Koch Institute (RKI); Zentrum für Künstliche Intelligenz in der Public Health-Forschung (ZKI-PH) | Germany | English | Scientific Association/Society | Conference session | ZKI-PH Symposium 2025 | In presence | 2025 | N | N | N | Y | N | N | N | Y | Session on AI-based strategies to overcome antimicrobial resistance: reducing antibiotic use and vaccine development. | <https://www.rki.de/EN/Institute/Organisation/Departments/ZKI-PH/Events/AIinPH-Symposium.html> | N |
| Regulatory Framework for AI and ML in Public Health | Robert Koch Institute (RKI); Zentrum für Künstliche Intelligenz in der Public Health-Forschung (ZKI-PH) | Germany | English | Scientific Association/Society | Conference session | ZKI-PH Symposium 2025 | In presence | 2025 | N | N | Y | N | Y | Y | N | Y | Session on regulatory framework for AI and machine learning in public health: EU AI Act and European Health Data Space. | <https://www.rki.de/EN/Institute/Organisation/Departments/ZKI-PH/Events/AIinPH-Symposium.html> | N |
| WebTalk: Cybersicherheit im Krankenhaus | Verband der Krankenhausdirektoren Deutschlands (VKD) | Germany | German | Scientific Association/Society | Seminar/Webinar | VKD-WebTalk | Online | 2025 | N | N | Y | N | Y | Y | N | N | Webinar on hospital cybersecurity: role and responsibilities of clinical managers. | <https://www.vkd-online.de/die-veranstaltungen/> | N |
| Management-Jour-Fixe: ePA – mehr Bürokratie oder echter Fortschritt? | Verband der Krankenhausdirektoren Deutschlands (VKD) | Germany | German | Scientific Association/Society | Seminar/Webinar | Management-Jour-Fixe | Online | 2025 | Y | N | Y | N | Y | Y | N | N | Online meeting for young hospital managers on electronic patient records (ePA): real progress or more bureaucracy? | <https://www.vkd-online.de/die-veranstaltungen/> | N |
| Management-Jour-Fixe: Robotik im Krankenhaus – Zukunftsvision oder bereits Alltag? | Verband der Krankenhausdirektoren Deutschlands (VKD) | Germany | German | Scientific Association/Society | Seminar/Webinar | Management-Jour-Fixe | Online | 2025 | Y | N | Y | N | N | N | N | Y | Online meeting for young hospital managers on robotics in hospitals: future vision or already routine? | <https://www.vkd-online.de/die-veranstaltungen/> | N |
| Telemedicina e digitalizzazione della sanità nei distretti | Confederazione Associazioni Regionali di Distretto (CARD) | Italy | Italian | Scientific Association/Society | Conference session | XIX Congresso Nazionale CARD / VI Conferenza Nazionale sulle Cure Domiciliari | In presence | 2021 | Y | N | Y | N | Y | Y | N | N | Session on electronic health records, PNRR, remote care, and telemedicine's role in chronic disease management and district service integration. | <http://www.carditalia.com/wp-content/uploads/2021/10/Il-Programma-CARD-Nazionale-Napoli-21-23-Ottobre-2021.pdf> | N |
| Impatto protocollo digitale su food literacy ed engagement pazienti diabetici | Società Italiana di Igiene, Medicina Preventiva e Sanità Pubblica (SItI) | Italy | Italian | Scientific Association/Society | Conference session | 54° Congresso Nazionale SItI 2021 | In presence | 2021 | N | Y | N | N | N | N | N | N | Presentation on a technological platform for diabetic patient education, with results from four Italian regions. | <http://www.sitinazionale.org/site/new/images/docs/atticongressi/2021/lecceprog.pdf> | N |
| Il ruolo delle tecnologie avanzate per il contact tracing | Società Italiana di Igiene, Medicina Preventiva e Sanità Pubblica (SItI) | Italy | Italian | Scientific Association/Society | Conference session | 54° Congresso Nazionale SItI 2021 | In presence | 2021 | Y | N | Y | N | N | N | N | N | Presentation on advanced technologies for contact tracing in pandemic prevention and response. | <http://www.sitinazionale.org/site/new/images/docs/atticongressi/2021/lecceprog.pdf> | N |
| Telemedicina per l'assistenza domiciliare e l'integrazione | Società Italiana di Igiene, Medicina Preventiva e Sanità Pubblica (SItI) | Italy | Italian | Scientific Association/Society | Conference session | 54° Congresso Nazionale SItI 2021 | In presence | 2021 | Y | N | Y | N | N | Y | N | N | Presentation on telemedicine for home care and integration, aligned with PNRR territorial healthcare objectives. | <http://www.sitinazionale.org/site/new/images/docs/atticongressi/2021/lecceprog.pdf> | N |
| Alfabetizzazione sanitaria digitale del personale comparto sanità | Società Italiana di Igiene, Medicina Preventiva e Sanità Pubblica (SItI) | Italy | Italian | Scientific Association/Society | Conference session | 55° Congresso Nazionale SItI 2022 | In presence | 2022 | N | Y | N | N | N | N | N | N | Presentation on digital health literacy of healthcare personnel in the context of healthcare-associated infections. | <http://www.sitinazionale.org/site/new/images/docs/atticongressi/2022/padovaprog.pdf> | N |
| Applicazioni GIS in Sanità pubblica: dall'emergenza alla funzionalità di routine | Società Italiana di Igiene, Medicina Preventiva e Sanità Pubblica (SItI) | Italy | Italian | Scientific Association/Society | Conference session | 55° Congresso Nazionale SItI 2022 | In presence | 2022 | Y | N | N | Y | Y | N | N | N | Presentation on GIS applications in public health: from emergency response to routine functionality. | <http://www.sitinazionale.org/site/new/images/docs/atticongressi/2022/padovaprog.pdf> | N |
| Big data e privacy in sanità pubblica: ostacolo o opportunità | Società Italiana di Igiene, Medicina Preventiva e Sanità Pubblica (SItI) | Italy | Italian | Scientific Association/Society | Conference session | 55° Congresso Nazionale SItI 2022 | In presence | 2022 | N | N | N | N | Y | Y | N | Y | Presentation on big data and privacy in public health: obstacle or opportunity. | <http://www.sitinazionale.org/site/new/images/docs/atticongressi/2022/padovaprog.pdf> | N |
| Comunicazione dati nell'era digitale ed e-Health delle reti ospedaliere e territoriali | Società Italiana di Igiene, Medicina Preventiva e Sanità Pubblica (SItI) | Italy | Italian | Scientific Association/Society | Conference session | 55° Congresso Nazionale SItI 2022 | In presence | 2022 | N | N | N | Y | Y | N | N | N | Presentation on data communication in the digital era and e-health of hospital and territorial networks. | <http://www.sitinazionale.org/site/new/images/docs/atticongressi/2022/padovaprog.pdf> | N |
| E-Health e integrazione alla luce del PNRR | Società Italiana di Igiene, Medicina Preventiva e Sanità Pubblica (SItI) | Italy | Italian | Scientific Association/Society | Conference session | 55° Congresso Nazionale SItI 2022 | In presence | 2022 | Y | N | N | N | N | Y | N | N | Presentation on e-health and integration in light of PNRR. | <http://www.sitinazionale.org/site/new/images/docs/atticongressi/2022/padovaprog.pdf> | N |
| Evoluzione digitale del management sanitario nella sfida alle ICA | Società Italiana di Igiene, Medicina Preventiva e Sanità Pubblica (SItI) | Italy | Italian | Scientific Association/Society | Conference session | 55° Congresso Nazionale SItI 2022 | In presence | 2022 | Y | N | Y | N | N | N | N | N | Presentation on digital management evolution in challenging healthcare-associated infections. | <http://www.sitinazionale.org/site/new/images/docs/atticongressi/2022/padovaprog.pdf> | N |
| Implementazione strumento monitoraggio digitale in tempo reale per percorso clinico tempo-dipendente | Società Italiana di Igiene, Medicina Preventiva e Sanità Pubblica (SItI) | Italy | Italian | Scientific Association/Society | Conference session | 55° Congresso Nazionale SItI 2022 | In presence | 2022 | Y | N | Y | N | N | N | N | N | Presentation on implementing real-time digital monitoring tools for time-dependent clinical pathways. | <http://www.sitinazionale.org/site/new/images/docs/atticongressi/2022/padovaprog.pdf> | N |
| Promesse e limitazioni delle applicazioni della Intelligenza Artificiale allo sviluppo dei trial clinici: revisione della letteratura in un'ottica di HTA | Società Italiana di Igiene, Medicina Preventiva e Sanità Pubblica (SItI) | Italy | Italian | Scientific Association/Society | Conference session | 55° Congresso Nazionale SItI 2022 | In presence | 2022 | Y | N | N | Y | Y | N | N | Y | Presentation on promises and limitations of AI applications in clinical trials from an HTA perspective. | <http://www.sitinazionale.org/site/new/images/docs/atticongressi/2022/padovaprog.pdf> | N |
| Digital Health e sanità Pubblica: prospettive e opportunità | Società Italiana di Igiene, Medicina Preventiva e Sanità Pubblica (SItI) | Italy | Italian | Scientific Association/Society | Conference session | 55° Congresso Nazionale SItI 2022 | In presence | 2022 | Y | Y | Y | N | N | N | Y | N | Presentation on digital health and public health: perspectives and opportunities. | <http://www.sitinazionale.org/site/new/images/docs/atticongressi/2022/padovaprog.pdf> | N |
| La sanità digitale nella evoluzione dei sistemi sanitari | Società Italiana di Igiene, Medicina Preventiva e Sanità Pubblica (SItI) | Italy | Italian | Scientific Association/Society | Conference session | 55° Congresso Nazionale SItI 2022 | In presence | 2022 | Y | N | Y | N | N | N | N | N | Presentation on digital health in the evolution of health systems. | <http://www.sitinazionale.org/site/new/images/docs/atticongressi/2022/padovaprog.pdf> | N |
| Explainable artificial intelligence per l'analisi esplorativa di dati epidemiologici | Associazione Italiana di Epidemiologia (AIE) | Italy | Italian | Scientific Association/Society | Seminar/Webinar | Convegno AIE Autunno 2023 - AI-Epidemiologia | In presence | 2023 | Y | N | N | Y | Y | N | N | Y | Workshop on explainable AI for exploratory analysis of epidemiological data. | <https://www.epidemiologia.it/notizie/convegno-aie-autunno-2023> | N |
| (Chat)GPT: understand the engine mastering its usage | Associazione Italiana di Epidemiologia (AIE) | Italy | Italian | Scientific Association/Society | Seminar/Webinar | Convegno AIE Autunno 2023 - AI-Epidemiologia | In presence | 2023 | Y | N | N | N | N | N | N | Y | Workshop on understanding and using ChatGPT and large language model technologies. | <https://www.epidemiologia.it/notizie/convegno-aie-autunno-2023> | N |
| Ricerca epidemiologica e Big Data: lo stato dell'arte | Associazione Italiana di Epidemiologia (AIE) | Italy | Italian | Scientific Association/Society | Seminar/Webinar | Convegno AIE Autunno 2023 - AI-Epidemiologia | In presence | 2023 | N | N | N | Y | Y | N | N | N | Presentation on the state of the art of epidemiological research with big data. | <https://www.epidemiologia.it/notizie/convegno-aie-autunno-2023> | N |
| Metodologie di intelligenza artificiale rilevanti in ambito epidemiologico | Associazione Italiana di Epidemiologia (AIE) | Italy | Italian | Scientific Association/Society | Seminar/Webinar | Convegno AIE Autunno 2023 - AI-Epidemiologia | In presence | 2023 | N | N | N | Y | Y | N | N | Y | Presentation on AI methodologies applicable to epidemiology. | <https://www.epidemiologia.it/notizie/convegno-aie-autunno-2023> | N |
| Utilizzi dell'intelligenza artificiale nell'epidemiologia delle malattie infettive | Associazione Italiana di Epidemiologia (AIE) | Italy | Italian | Scientific Association/Society | Seminar/Webinar | Convegno AIE Autunno 2023 - AI-Epidemiologia | In presence | 2023 | Y | N | N | Y | N | N | N | Y | Presentation on AI applications in infectious disease epidemiology. | <https://www.epidemiologia.it/notizie/convegno-aie-autunno-2023> | N |
| Master di II livello in Management dell'Innovazione in Sanità | ALTEMS - Università Cattolica del Sacro Cuore | Italy | Italian | School/University | Training course |  | Hybrid | 2024 | N | N | Y | N | Y | Y | N | Y | Executive master (post-master's) on healthcare innovation management with 'Digital Health' module, training professionals in evidence-based technology adoption. | <https://altems.unicatt.it/altems-master-management-dell-innovazione-in-sanita> | N |
| Valutazione delle applicazioni dell'intelligenza artificiale in sanità | ALTEMS - Università Cattolica del Sacro Cuore | Italy | Italian | School/University | Training course |  | Hybrid | 2024 | N | N | N | N | Y | Y | N | Y | Advanced training course on evaluating AI applications in healthcare using HTA methodology adapted for AI and machine learning, covering regulatory processes and data management in the EHDS context. | <https://altems.unicatt.it/altems-corsi-di-alta-formazione-valutazione-delle-applicazioni-dell-intelligenza-artificiale-in-sanita> | N |
| Sanità 4.0 - Tecnologie e IA per migliore assistenza al paziente | ALTIS Graduate School of Sustainable Management - Università Cattolica del Sacro Cuore | Italy | Italian | School/University | Training course |  | Hybrid | 2024 | Y | N | Y | N | N | N | N | Y | Course on Healthcare 4.0: technologies and AI for improved patient care. | <https://altis.unicatt.it/altis-2024-sanita-4-0-tecnologie-e-ia-per-una-migliore-assistenza-al-paziente> | N |
| Quali innovazioni tecnologiche nei distretti? | Confederazione Associazioni Regionali di Distretto (CARD) | Italy | Italian | Scientific Association/Society | Conference session | XXII Congresso Nazionale CARD | In presence | 2024 | Y | N | Y | N | N | N | N | Y | Session on technological innovations in health districts and related organisational changes. | <http://www.carditalia.com/wp-content/uploads/2024/04/Il-Programma-Genova-17-19-Ottobre-2024.pdf> | N |
| Innovazione e digitale: percorsi e obiettivi per disegnare il futuro della salute | Confederazione Associazioni Regionali di Distretto - Puglia (CARD Puglia) | Italy | Italian | Scientific Association/Society | Conference session | XIX Congresso CARD Puglia | In presence | 2024 | Y | N | Y | N | N | Y | N | Y | Session on how innovation and digital tools can define pathways and objectives for the future of the health system. | <https://www.e20econvegni.it/wp-content/uploads/2024/10/brochure-CARD-9.pdf> | N |
| Sanità digitale e telemedicina | E-Campus Università - CERFAS | Italy | Italian | School/University | Training course |  | Online | 2024 | Y | Y | Y | Y | Y | Y | N | N | Professional master (post-bachelor's) on digital health and telemedicine. | [https://ecm.uniecampus.it/master/sanita-digitale-e-telemedicina.asp](https://ecm.uniecampus.it/master/sanita-digitale-e-telemedicina.asp?adsmonitorid=122420&gad_source=1&gclid=Cj0KCQjw2N2_BhCAARIsAK4pEkUX-i9Ul0f_7xy1EfNpI-6NGkNem49h7TM_FqfCzoK_ouctErLbzTUaApZYEALw_wcB) | N |
| Introduzione all'Intelligenza Artificiale per operatori sanitari | EduISS - Istituto Superiore di Sanità | Italy | Italian | Scientific Association/Society | Training course |  | Online | 2024 | Y | N | N | N | N | N | N | Y | Introductory course on AI for healthcare workers: concepts, applications and basic tools. | <https://www.eduiss.it/theme/tcontinuum/infocourse.php?course=535&popup=1> | N |
| Master: Sanità digitale e supporto all'attività territoriale | IGEA Centro Promozione Salute | Italy | Italian | School/University | Training course |  | Online | 2024 | Y | N | Y | Y | Y | N | N | N | Professional master (post-bachelor's, online) on digital health for community and territorial services: telemedicine, AI and data management. | <https://www.igeacps.it/sanita-digitale-e-supporto-allattivita-territoriale-master-di-1-livello/> | N |
| La digitalizzazione a supporto degli obiettivi di Sanità pubblica: governare l'innovazione tecnologica verso il miglior modello di prevenzione | Società Italiana di Igiene, Medicina Preventiva e Sanità Pubblica (SItI) | Italy | Italian | Scientific Association/Society | Conference session | 57° Congresso Nazionale SItI 2024 | In presence | 2024 | Y | N | Y | N | N | N | N | Y | Plenary session on governance of digital and technological innovation for prevention. | <https://pro.campus.sanofi/dam/Portal/Italy/eventi/Congressi---eventi-2024/SITI--Palermo-.pdf> | N |
| Digital Public Health: esplorare il futuro della sanità pubblica con l'intelligenza artificiale | Società Italiana di Igiene, Medicina Preventiva e Sanità Pubblica (SItI) | Italy | Italian | Scientific Association/Society | Conference session | 57° Congresso Nazionale SItI 2024 | In presence | 2024 | Y | Y | Y | N | N | N | N | Y | Workshop on large language model potential in public health with practical applications of LLMs, AI chatbots and CustomGPT development. | <https://pro.campus.sanofi/dam/Portal/Italy/eventi/Congressi---eventi-2024/SITI--Palermo-.pdf> | N |
| Prevedere per prevenire: Epidemic Intelligence | Società Italiana di Igiene, Medicina Preventiva e Sanità Pubblica (SItI); Regione Lombardia | Italy | Italian | Scientific Association/Society | Conference session | Innovazione in Prevenzione | Hybrid | 2024 | Y | N | N | Y | N | N | N | Y | Session on epidemic intelligence and predictive alert systems in the Lombardy Region. | <https://eventi.regione.lombardia.it/it/innovazione-in-prevenzione> | N |
| Smart prevention: l'AI al servizio della salute | Società Italiana di Igiene, Medicina Preventiva e Sanità Pubblica (SItI); Regione Lombardia | Italy | Italian | Scientific Association/Society | Conference session | Innovazione in Prevenzione | Hybrid | 2024 | Y | N | N | Y | N | N | N | Y | Session on AI in research and prevention with focus on Lombardy applications. | <https://eventi.regione.lombardia.it/it/innovazione-in-prevenzione> | N |
| Personalized Training of Professional Competencies with AI | Università del Piemonte Orientale (UPO) | Italy | Italian | School/University | Training course |  | Hybrid | 2024 | Y | N | N | N | N | N | N | Y | Course on AI in healthcare for personalised training of professional competencies. | <https://mediacentre.uniupo.it/it/agenda/corso-sullapplicazione-dellintelligenza-artificiale-sanita-personalized-training-professional> | N |
| Master in Digital Transformation nella Sanità | ALTEMS - Università Cattolica del Sacro Cuore | Italy | Italian | School/University | Training course |  | In presence | 2024 | Y | Y | Y | Y | N | N | N | Y | Master providing multidisciplinary training in big data analysis, AI for diagnostics and treatment, and digital health management strategies for graduates in medicine, biomedical engineering and health economics. | <https://www.masterin.it/start/5439-l-importanza-della-digital-transformation-nella-sanita-focus-sul-master-dell-universita-cattolica/> | N |
| Intelligenza artificiale per prevenire emergenze sanitarie - progetto Trust Alert | Università di Torino (UniTo) | Italy | Italian | School/University | Seminar/Webinar |  | Online | 2024 | Y | N | N | N | N | N | N | Y | Presentation of Trust Alert project initial results on using AI to prevent health emergencies. | <https://www.unito.it/comunicati_stampa/intelligenza-artificiale-prevenire-le-emergenze-sanitarie-i-primi-risultati-del> | N |
| Master di II livello in Management dell'Innovazione in Sanità | ALTEMS - Università Cattolica del Sacro Cuore | Italy | Italian | School/University | Training course |  | Hybrid | 2025 | N | N | Y | N | Y | Y | N | Y | Executive master (post-master's) on healthcare innovation management with 'Digital Health' module, training professionals in evidence-based technology adoption. | <https://altems.unicatt.it/altems-master-management-dell-innovazione-in-sanita> | N |
| Valutazione delle applicazioni dell'intelligenza artificiale in sanità | ALTEMS - Università Cattolica del Sacro Cuore | Italy | Italian | School/University | Training course |  | Hybrid | 2025 | N | N | Y | N | Y | Y | N | Y | Advanced training course on evaluating AI applications in healthcare using HTA methodology adapted for AI and machine learning, covering regulatory processes and data management in the EHDS context. | <https://altems.unicatt.it/altems-corsi-di-alta-formazione-valutazione-delle-applicazioni-dell-intelligenza-artificiale-in-sanita> | N |
| Intelligenza umana e intelligenza artificiale: quale futuro? | Associazione Nazionale Medici Direzioni Ospedaliere (ANMDO) | Italy | Italian | Scientific Association/Society | Conference session | 50° Congresso Nazionale ANMDO | In presence | 2025 | Y | N | Y | N | N | Y | N | Y | Lecture comparing human and artificial intelligence and future perspectives. | <https://www.anmdo.org/wp-content/uploads/2025/07/25-07-08-Programma-Avanzato_rev.pdf> | N |
| I nuovi trasporti sanitari: l'utilizzo dei droni nelle operazioni di soccorso | Associazione Nazionale Medici Direzioni Ospedaliere (ANMDO) | Italy | Italian | Scientific Association/Society | Conference session | 50° Congresso Nazionale ANMDO | In presence | 2025 | Y | N | Y | N | N | N | N | N | Presentation on technological innovation in emergency healthcare through drone use for rescue operations. | <https://www.anmdo.org/wp-content/uploads/2025/07/25-07-08-Programma-Avanzato_rev.pdf> | N |
| Robotica a supporto dell'accoglienza dei pazienti fragili | Associazione Nazionale Medici Direzioni Ospedaliere (ANMDO) | Italy | Italian | Scientific Association/Society | Conference session | 50° Congresso Nazionale ANMDO | In presence | 2025 | Y | Y | Y | N | N | N | N | N | Presentation on robotics for improving reception and care for frail patients. | <https://www.anmdo.org/wp-content/uploads/2025/07/25-07-08-Programma-Avanzato_rev.pdf> | N |
| L'ospedale virtuale e digitale: esperienze e prospettive | Associazione Nazionale Medici Direzioni Ospedaliere (ANMDO) | Italy | Italian | Scientific Association/Society | Conference session | 50° Congresso Nazionale ANMDO | In presence | 2025 | Y | Y | Y | N | Y | N | N | N | Presentation on experiences and perspectives of digital and virtual hospitals. | <https://www.anmdo.org/wp-content/uploads/2025/07/25-07-08-Programma-Avanzato_rev.pdf> | N |
| L'intelligenza artificiale a supporto del medico per generare più tempo per i pazienti | Associazione Nazionale Medici Direzioni Ospedaliere (ANMDO) | Italy | Italian | Scientific Association/Society | Conference session | 50° Congresso Nazionale ANMDO | In presence | 2025 | Y | N | Y | Y | N | N | N | N | Lecture on how AI can optimise physician time to dedicate more to patients. | <https://www.anmdo.org/wp-content/uploads/2025/07/25-07-08-Programma-Avanzato_rev.pdf> | N |
| La prevenzione delle cadute accidentali dei pazienti, l'efficacia dell'intelligenza artificiale | Associazione Nazionale Medici Direzioni Ospedaliere (ANMDO) | Italy | Italian | Scientific Association/Society | Conference session | 50° Congresso Nazionale ANMDO | In presence | 2025 | Y | N | N | Y | N | N | N | N | Lecture on AI for preventing accidental patient falls in hospitals. | <https://www.anmdo.org/wp-content/uploads/2025/07/25-07-08-Programma-Avanzato_rev.pdf> | N |
| Miglioramento della efficienza operativa tramite intelligenza artificiale | Associazione Nazionale Medici Direzioni Ospedaliere (ANMDO) | Italy | Italian | Scientific Association/Society | Conference session | 50° Congresso Nazionale ANMDO | In presence | 2025 | Y | N | Y | Y | Y | N | N | N | Lecture on using AI to optimise operational efficiency in healthcare. | <https://www.anmdo.org/wp-content/uploads/2025/07/25-07-08-Programma-Avanzato_rev.pdf> | N |
| La minaccia cyber alla sanità: dalla gestione dei rischi alla gestione delle emergenze | Associazione Nazionale Medici Direzioni Ospedaliere (ANMDO) | Italy | Italian | Scientific Association/Society | Conference session | 50° Congresso Nazionale ANMDO | In presence | 2025 | Y | Y | Y | N | Y | Y | N | N | Presentation on cyber threat management in healthcare: risk management and emergency response. | <https://www.anmdo.org/wp-content/uploads/2025/07/25-07-08-Programma-Avanzato_rev.pdf> | N |
| La Governance centrale della sanità digitale: aspetti etici | Associazione Nazionale Medici Direzioni Ospedaliere (ANMDO) | Italy | Italian | Scientific Association/Society | Conference session | 50° Congresso Nazionale ANMDO | In presence | 2025 | N | N | Y | N | Y | Y | N | Y | Presentation on central governance of digital health with focus on ethical aspects. | <https://www.anmdo.org/wp-content/uploads/2025/07/25-07-08-Programma-Avanzato_rev.pdf> | N |
| Intelligenza artificiale in sanità: attualità e prospettive | Associazione Nazionale Medici Direzioni Ospedaliere (ANMDO) | Italy | Italian | Scientific Association/Society | Conference session | 50° Congresso Nazionale ANMDO | In presence | 2025 | Y | Y | Y | Y | Y | N | N | Y | Presentation on current state and future perspectives of AI in healthcare. | <https://www.anmdo.org/wp-content/uploads/2025/07/25-07-08-Programma-Avanzato_rev.pdf> | N |
| Inquadramento normativo e IA | Associazione Nazionale Medici Direzioni Ospedaliere (ANMDO) | Italy | Italian | Scientific Association/Society | Conference session | 50° Congresso Nazionale ANMDO | In presence | 2025 | N | N | N | N | N | Y | N | Y | Presentation on the legal and regulatory framework for AI in healthcare. | <https://www.anmdo.org/wp-content/uploads/2025/07/25-07-08-Programma-Avanzato_rev.pdf> | N |
| La Direzione Sanitaria tra ricerca, innovazione e decisioni strategiche: dal dato al valore | Associazione Nazionale Medici Direzioni Ospedaliere (ANMDO) | Italy | Italian | Scientific Association/Society | Seminar/Webinar | Webinar FAD di 4h | Online | 2025 | N | Y | Y | N | Y | Y | N | N | Webinar on using clinical, managerial and epidemiological data to create value, ensuring ethics, privacy and sustainability. Covers dashboards, predictive tools, legal aspects, information systems and e-health. | <https://www.anmdo.org/wp-content/uploads/2025/11/programma-FAD-467640-r6.pdf> | N |
| Introduzione all'Intelligenza Artificiale in Sanità | Azienda Ospedaliero-Universitaria delle Marche | Italy | Italian | School/University | Training course |  | In presence | 2025 | Y | N | N | N | N | N | N | Y | Introductory course on AI in healthcare for hospital staff. | <https://portale.ospedaliriuniti.marche.it/archivio3_congressi-seminari-e-corsi_0_267.html> | N |
| WORKSHOP: IA, Formazione e ricerca: sfide ed opportunità per la sanità pubblica | Società Italiana di Igiene, Medicina Preventiva e Sanità Pubblica (SItI) | Italy | Italian | Scientific Association/Society | Conference session | 58° Congresso Nazionale SItI 2025 | In presence | 2025 | Y | Y | Y | N | N | Y | N | Y | Workshop on AI in public health training and research, focusing on evaluation and scientific publishing. | <https://siti2025.it/Download/EasyCms/Programma%20Gestionale%20R21_NS_67754.pdf> | N |
| WORKSHOP: Intelligenza Artificiale nella sintesi delle evidenze: uno skill building workshop per supportare la ricerca, le decisioni e gli interventi di sanità pubblica | Società Italiana di Igiene, Medicina Preventiva e Sanità Pubblica (SItI) | Italy | Italian | Scientific Association/Society | Conference session | 58° Congresso Nazionale SItI 2025 | In presence | 2025 | Y | Y | Y | Y | Y | N | Y | Y | Practical workshop on AI for evidence synthesis, prompt engineering and named entity recognition. | <https://siti2025.it/Download/EasyCms/Programma%20Gestionale%20R21_NS_67754.pdf> | N |
| Corso di Alta formazione: Valutazione delle applicazioni dell'intelligenza artificiale in sanità | ALTEMS - Università Cattolica del Sacro Cuore | Italy | Italian | School/University | Training course |  | Hybrid | 2025 | N | N | Y | Y | Y | Y | N | Y | Advanced training course (ALTEMS) on evaluating AI applications in healthcare from an HTA perspective. | <https://altems.unicatt.it/altems-corsi-di-alta-formazione-valutazione-delle-applicazioni-dell-intelligenza-artificiale-in-sanita> | N |
| Master: DAI4Health - Innovazione digitale e intelligenza artificiale nel settore sanitario | Università di Milano-Bicocca (UniMiB) | Italy | Italian | School/University | Training course |  | In presence | 2025 | Y | N | Y | Y | Y | Y | N | Y | Master on digital innovation and AI: machine learning, NLP, big data, robotics and ethical-legal challenges in healthcare. | <https://academy.unimib.it/dai4health-innovazione-digitale-e-intelligenza-artificiale-nel-settore-sanitario-0> | N |
| Master: Intelligenza artificiale e telemedicina | Università di Parma (UniPR) | Italy | Italian | School/University | Training course |  | Online | 2025 | Y | N | Y | Y | Y | Y | N | Y | Professional master (post-bachelor's, online) on AI and telemedicine: applications, opportunities and challenges in healthcare. | <https://corsi.unipr.it/it/node/16656> | N |
| Digital Health, intelligenza artificiale e big data. Opportunità e rischi, tenendo conto della tutela della privacy | Università di Parma (UniPR) | Italy | Italian | School/University | Seminar/Webinar |  | In presence | 2024 | N | Y | N | N | N | Y | N | Y | Seminar on digital health, AI and big data: opportunities and risks considering privacy protection. | NA | Y |
| Digital Health, intelligenza artificiale e big data. Opportunità e rischi, tenendo conto della tutela della privacy | Università di Parma (UniPR) | Italy | Italian | School/University | Seminar/Webinar |  | In presence | 2025 | N | Y | N | N | N | Y | N | Y | Seminar on digital health, AI and big data: opportunities and risks considering privacy protection. | NA | Y |
| Sistemi decisionali in medicina | Università di Pavia (UniPV) | Italy | Italian | School/University | Lecture series |  | In presence | 2022 | Y | N | Y | Y | N | N | N | N | Course on modelling decision-making processes in medicine: decision trees, influence diagrams and dedicated software. | [http://www-3.unipv.it/ingserv//didattica/schedacorso0607.php?cod=064091.](http://www-3.unipv.it/ingserv/didattica/schedacorso0607.php?cod=064091.) | Y |
| The European Health Data Space | Università di Pavia (UniPV) | Italy | Italian | School/University | Lecture series |  | In presence | 2022 | N | N | N | N | Y | Y | N | N | Peer-to-peer presentation on the European Health Data Space. | NA | Y |
| Applications of AI in Healthcare | Escuela Andaluza de Salud Pública (EASP) | Spain | Spanish | School/University | Training course |  | Online | 2020 | Y | N | N | N | N | N | N | Y | MOOC on AI applications in healthcare. | <https://medicinaprecisionandalucia.easp.es/course/view.php?id=230&section=1#tabs-tree-start> | N |
| Health Services Management and Digital Transformation | Escuela Andaluza de Salud Pública (EASP) | Spain | Spanish | School/University | Training course |  | In presence | 2020 | Y | N | Y | N | Y | Y | N | Y | Second-year specialisation of Europubhealth+ Master on health services management and digital transformation, combining traditional management with AI and digitalisation training. | [https://www.europubhealth.org/second-year-specialisations/healthservicesmanagement/ / https://www.europubhealth.org/wp-content/uploads/2024/10/Syllabus_-Granada-Y2_.pdf](https://www.europubhealth.org/second-year-specialisations/healthservicesmanagement/) | N |
| Health Services Management and Digital Transformation | Escuela Andaluza de Salud Pública (EASP) | Spain | Spanish | School/University | Training course |  | In presence | 2021 | Y | N | Y | N | Y | Y | N | Y | Second-year specialisation of Europubhealth+ Master on health services management and digital transformation, combining traditional management with AI and digitalisation training. | [https://www.europubhealth.org/second-year-specialisations/healthservicesmanagement/ / https://www.europubhealth.org/wp-content/uploads/2024/10/Syllabus_-Granada-Y2_.pdf](https://www.europubhealth.org/second-year-specialisations/healthservicesmanagement/) | N |
| Evaluando la inteligencia artificial: ¿es adecuado aplicar el enfoque metodológico tradicional de evaluación de tecnologías sanitarias? | Asociación de Economía de la Salud (AES) | Spain | Spanish | Scientific Association/Society | Conference session | XL Jornadas de Economía de la Salud | Online | 2021 | N | N | N | N | N | N | N | Y | Session on appropriateness of applying traditional HTA methodology to AI evaluation. | <https://www.aes.es/Jornadas2021/es/> | N |
| El derecho ante la inteligencia articial en el àmbito sanitario | Asociación Juristas de la Salud (AJS) | Spain | Spanish | Scientific Association/Society | Conference session | XXIX Congreso Derecho y Salud | In presence | 2021 | N | N | N | N | Y | N | N | Y | Session on use of patients' personal and health data in AI, privacy and fundamental rights of patients in AI applications and Ethical-legal overview of AI in health from big data to robotics. | <https://www.ajs.es/index.php/es/index-congresos/xxix-congreso-derecho-y-salud> | N |
| Health Services Management and Digital Transformation | Escuela Andaluza de Salud Pública (EASP) | Spain | Spanish | School/University | Training course |  | In presence | 2022 | Y | N | Y | N | Y | Y | N | Y | Second-year specialisation of Europubhealth+ Master on health services management and digital transformation, combining traditional management with AI and digitalisation training. | [https://www.europubhealth.org/second-year-specialisations/healthservicesmanagement/ / https://www.europubhealth.org/wp-content/uploads/2024/10/Syllabus_-Granada-Y2_.pdf](https://www.europubhealth.org/second-year-specialisations/healthservicesmanagement/) | N |
| Machine Learning para analizar con eficiencia la variabilidad clìnic | Asociación de Economía de la Salud (AES) | Spain | Spanish | Scientific Association/Society | Conference session | XL+1 Jornadas AES | In presence | 2022 | Y | N | N | N | N | N | N | Y | Session on machine learning as clinical decision support to analyse clinical variability. | <https://www.aes.es/Jornadas2022/es/> | N |
| Data governance for RWD/E Management: optimising its use in HTA and regulatory decision-making | Asociación de Economía de la Salud (AES) | Spain | Spanish | Scientific Association/Society | Conference session | XL+1 Jornadas AES | In presence | 2022 | N | N | Y | N | Y | N | N | N | Session on data governance for RWD/E management in HTA and regulatory decision-making. | <https://www.aes.es/Jornadas2022/es/> | N |
| La inteligencia artificial (IA) como aplicaciòn juridìdica plausibile | Asociación Juristas de la Salud (AJS) | Spain | Spanish | Scientific Association/Society | Conference session | XXX Congreso Derecho y Salud | In presence | 2022 | N | N | N | N | N | Y | N | Y | Session on AI as a plausible legal application. | <https://www.ajs.es/sites/default/files/2022-09/programa%202022%20web.pdf> | N |
| Health Services Management and Digital Transformation | Escuela Andaluza de Salud Pública (EASP) | Spain | Spanish | School/University | Training course |  | In presence | 2023 | Y | N | N | N | Y | Y | N | Y | Second-year specialisation of Europubhealth+ Master on health services management and digital transformation, combining traditional management with AI and digitalisation training. | [https://www.europubhealth.org/second-year-specialisations/healthservicesmanagement/ / https://www.europubhealth.org/wp-content/uploads/2024/10/Syllabus_-Granada-Y2_.pdf](https://www.europubhealth.org/second-year-specialisations/healthservicesmanagement/) | N |
| Evaluación de tecnologías sanitarias: aportaciones de la IA y la medicina de precisión | Asociación de Economía de la Salud (AES) | Spain | Spanish | Scientific Association/Society | Conference session | XLIV Jornadas AES | In presence | 2023 | N | N | Y | N | N | N | N | Y | Session on technology evauations and precision medicine. | <https://www.aes.es/jornadas/es/> | N |
| Inteligencia Artificial aplicada a la salud. Oportunidades para la Enfermería Familiar y Comunitaria. | Asociación de Enfermería Comunitaria (AEC) | Spain | Spanish | Scientific Association/Society | Conference session | VII Congreso Internacional AEC | In presence | 2023 | Y | N | N | N | N | N | N | Y | Session on AI applied to health as an opportunity for community nurses. | <https://www.enfermeriacomunitaria.org/web/index.php/congreso2023-programa> | N |
| Sesion Pleanria: Inteligencia artificial en sanidad | Asociación Juristas de la Salud (AJS) | Spain | Spanish | Scientific Association/Society | Conference session | XXXI Congreso Derecho y Salud | In presence | 2023 | Y | N | N | N | N | Y | N | Y | Plenary session on AI application to health. | <https://www.ajs.es/sites/default/files/2023-05/Programa%20Congreso%202023_05_26.pdf> | N |
| Mesa redonda: Investigación con datos digitales y nuevas tecnologías | Escuela Nacional de Sanidad (ENS); Instituto de Salud Carlos III (ISCIII) | Spain | Spanish | School/University | Conference session | VII Congreso sobre Ética de la Investigación Biomédica | In presence | 2023 | N | N | N | N | Y | Y | N | Y | Session on digital data and new technologies in research: ethics and human rights. | <https://ens.isciii.es/documents/d/guest/programa_definitivo_2023> | N |
| Digital health | Universidad Pública de Navarra (UPNA) | Spain | Spanish | School/University | Seminar/Webinar | II Seminario Abierto de Salud Digital | Hybrid | 2023 | Y | Y | Y | N | N | N | N | N | Seminar on digital health. | <https://www2.unavarra.es/gesadj/seccionActualidad/Agenda/II%20SEMINARIO%20ABIERTO%20DE%20SALUD%20DIGITAL%20PROGRAMA.pdf> | N |
| Liderando la democratización de los datos: la inteligencia artificial para la Medicina Preventiva y la Salud Pública | Sociedad Española de Medicina Preventiva, Salud Pública y Gestión Sanitaria (SEMPSPGS) | Spain | Spanish | Scientific Association/Society | Conference session | XXII Congreso Nacional y XI Congreso Internacional de la SEMPSPGS | In presence | 2023 | N | N | Y | Y | Y | Y | N | Y | Roundtable on data democratisation and AI in preventive medicine and public health, with big data use cases. | <https://sempspgs.es/wp-content/uploads/2025/05/XXII-Congreso-Nacional-XI-Internacional-Palma-2023.pdf> | N |
| Manos Seguras 3.0: Una App capaz de generar informes automáticos, divulgar, formar y avanzar en la prevención de la transmisión de IRAS mediante Inteligencia Artificial | Sociedad Española de Medicina Preventiva, Salud Pública y Gestión Sanitaria (SEMPSPGS) | Spain | Spanish | Scientific Association/Society | Conference session | XXII Congreso Nacional y XI Congreso Internacional de la SEMPSPGS | In presence | 2023 | Y | N | N | N | N | N | N | N | Symposium on 'Manos Seguras 3.0' app using AI for automatic reports and prevention of healthcare-associated infections. | <https://sempspgs.es/wp-content/uploads/2025/05/XXII-Congreso-Nacional-XI-Internacional-Palma-2023.pdf> | N |
| Health Services Management and Digital Transformation | Escuela Andaluza de Salud Pública (EASP) | Spain | Spanish | School/University | Training course |  | In presence | 2024 | N | N | Y | N | N | Y | N | Y | Second-year specialisation of Europubhealth+ Master on health services management and digital transformation, combining traditional management with AI and digitalisation training. | [https://www.europubhealth.org/second-year-specialisations/healthservicesmanagement/ / https://www.europubhealth.org/wp-content/uploads/2024/10/Syllabus_-Granada-Y2_.pdf](https://www.europubhealth.org/second-year-specialisations/healthservicesmanagement/) | N |
| Causalidad y Machine Learning en R | Asociación de Economía de la Salud (AES) | Spain | Spanish | Scientific Association/Society | Training course | XLIII Jornadas AES | In presence | 2024 | Y | N | N | Y | Y | N | N | N | Full-day session on causality and machine learning in R. | <https://www.aes.es/Jornadas2024/es/> | N |
| Ciberseguridad, intrusiones ilícitas y riesgos para el funcionamiento de los servicios sanitarios | Asociación Juristas de la Salud (AJS) | Spain | Spanish | Scientific Association/Society | Conference session | XXXI Congreso Derecho y Salud | In presence | 2023 | Y | N | Y | N | Y | N | N | N | Plenary session on cybersecurity, illicit intrusions and risks to healthcare service operations. | <https://www.ajs.es/sites/default/files/2023-05/Programa%20Congreso%202023_05_26.pdf> | N |
| Inteligencia Artificial en Sanidad | Asociación Juristas de la Salud (AJS) | Spain | Spanish | Scientific Association/Society | Conference session | XXXI Congreso Derecho y Salud | In presence | 2023 | N | N | N | Y | N | Y | N | Y | Plenary session on AI in healthcare. | <https://www.ajs.es/sites/default/files/2023-05/Programa%20Congreso%202023_05_26.pdf> | N |
| Inteligencia Artificial y Genómica: Aspectos Éticos y Legales | Asociación Juristas de la Salud (AJS) | Spain | Spanish | Scientific Association/Society | Conference session | XXXII Congreso Derecho y Salud "Salud Digital: Oportunidades vs Riesgos" | In presence | 2024 | N | N | N | Y | Y | Y | N | Y | Plenary session on ethical and legal aspects of AI applied to genomics. | <https://ajs.es/sites/default/files/2024-05/Programa%20Congreso%202024.pdf> | N |
| Salud Digital y Seguridad del Paciente | Asociación Juristas de la Salud (AJS); Asociación Española de Gestión de Riesgos Sanitarios (AEGRIS) | Spain | Spanish | Scientific Association/Society | Conference session | XXXII Congreso Derecho y Salud "Salud Digital: Oportunidades vs Riesgos" | In presence | 2024 | Y | N | Y | N | N | Y | N | N | Working table on digital health and patient safety: ethical, medico-legal aspects and impact of health insurance in the digital era. | <https://ajs.es/sites/default/files/2024-05/Programa%20Congreso%202024.pdf> | N |
| La Ciberseguridad en las Instituciones: Diversas Experiencias en la Defensa de los Datos | Asociación Juristas de la Salud (AJS) | Spain | Spanish | Scientific Association/Society | Conference session | XXXII Congreso Derecho y Salud "Salud Digital: Oportunidades vs Riesgos" | In presence | 2024 | Y | N | Y | N | Y | N | N | N | Working table on institutional cybersecurity with practical experiences from Valencia Community, Alicante Municipality and Grupo Ribera on digital transformation and health data protection. | <https://ajs.es/sites/default/files/2024-05/Programa%20Congreso%202024.pdf> | N |
| La Transformación Digital en el Ámbito Sanitario | Asociación Juristas de la Salud (AJS) | Spain | Spanish | Scientific Association/Society | Conference session | XXXII Congreso Derecho y Salud "Salud Digital: Oportunidades vs Riesgos" | In presence | 2024 | Y | N | Y | N | Y | N | N | N | Plenary session on digital transformation in healthcare. | <https://ajs.es/sites/default/files/2024-05/Programa%20Congreso%202024.pdf> | N |
| Inteligencia Artificial, Injusticia Algorítmica y Salud Pública | Asociación Juristas de la Salud (AJS); Sociedad Española de Salud Pública y Administración Sanitaria (SESPAS) | Spain | Spanish | Scientific Association/Society | Conference session | XXXII Congreso Derecho y Salud "Salud Digital: Oportunidades vs Riesgos" | In presence | 2024 | N | Y | N | Y | N | Y | N | Y | Working table on AI, algorithmic injustice and public health. | <https://ajs.es/sites/default/files/2024-05/Programa%20Congreso%202024.pdf> | N |
| La Garantía Judicial del Ordenamiento Sanitario en Tiempos Digitales | Asociación Juristas de la Salud (AJS) | Spain | Spanish | Scientific Association/Society | Conference session | XXXII Congreso Derecho y Salud "Salud Digital: Oportunidades vs Riesgos" | In presence | 2024 | N | N | N | N | N | Y | N | N | Plenary session on judicial guarantee of health regulations in digital times. | <https://ajs.es/sites/default/files/2024-05/Programa%20Congreso%202024.pdf> | N |
| Datos y Secretos del Paciente, su Protección en la Red Sanitaria y Repercusiones Penales ante Incumplimientos | Asociación Juristas de la Salud (AJS) | Spain | Spanish | Scientific Association/Society | Conference session | XXXII Congreso Derecho y Salud "Salud Digital: Oportunidades vs Riesgos" | In presence | 2024 | N | N | N | N | Y | Y | N | N | Plenary session on patient data protection in health networks and criminal consequences for violations. | <https://ajs.es/sites/default/files/2024-05/Programa%20Congreso%202024.pdf> | N |
| Los Datos de Salud ante el Nuevo Reglamento de Inteligencia Artificial | Asociación Juristas de la Salud (AJS) | Spain | Spanish | Scientific Association/Society | Conference session | XXXII Congreso Derecho y Salud "Salud Digital: Oportunidades vs Riesgos" | In presence | 2024 | N | N | N | Y | Y | Y | N | Y | Working table on health data under the EU AI Act. | <https://ajs.es/sites/default/files/2024-05/Programa%20Congreso%202024.pdf> | N |
| La Aplicación de la Inteligencia Artificial a la Sanidad y Problemáticas Jurídicas que Plantea | Asociación Juristas de la Salud (AJS) | Spain | Spanish | Scientific Association/Society | Conference session | XXXII Congreso Derecho y Salud "Salud Digital: Oportunidades vs Riesgos" | In presence | 2024 | N | N | N | N | N | Y | N | Y | Working table on AI application in healthcare and legal issues. | <https://ajs.es/sites/default/files/2024-05/Programa%20Congreso%202024.pdf> | N |
| Derechos Fundamentales en el Ámbito de la Salud Digital | Asociación Juristas de la Salud (AJS) | Spain | Spanish | Scientific Association/Society | Conference session | XXXII Congreso Derecho y Salud "Salud Digital: Oportunidades vs Riesgos" | In presence | 2024 | N | Y | N | N | N | Y | N | N | Plenary session on fundamental rights in digital health. | <https://ajs.es/sites/default/files/2024-05/Programa%20Congreso%202024.pdf> | N |
| Nuevos Paradigmas: Evidencia del mundo real e inteligencia artificial, el cambio de paradigma en la generación de conocimiento | Sociedad Española de Epidemiología (SEE); Associação Portuguesa de Epidemiologia (APE) | Spain | Spanish | Scientific Association/Society | Conference session | XLII Reunión Anual SEE / XIX Congresso APE | In presence | 2024 | N | N | N | Y | N | N | N | Y | Presentation on AI and real-world evidence as a paradigm shift in knowledge generation for public health. | <https://www.reunionanualsee.org/2024/documentos/ProgramaEpi24.pdf> | N |
| Nuevos Paradigmas: Medicina y Salud Pública de Precisión | Sociedad Española de Epidemiología (SEE); Associação Portuguesa de Epidemiologia (APE) | Spain | Spanish | Scientific Association/Society | Conference session | XLII Reunión Anual SEE / XIX Congresso APE | In presence | 2024 | N | N | N | Y | N | N | N | N | Presentation on precision medicine and precision public health: molecular biology, genomic sequencing, bioinformatics and big data. | <https://www.reunionanualsee.org/2024/documentos/ProgramaEpi24.pdf> | N |
| Las posibilidades de la inteligencia artificial en el abordaje de enfermedades tropicales desatendida | Escuela Nacional de Sanidad (ENS); Instituto de Salud Carlos III (ISCIII) | Spain | Spanish | School/University | Seminar/Webinar |  | Online | 2024 | Y | N | N | N | N | N | N | N | Seminar on AI for addressing neglected tropical diseases. | <https://www.isciii.es/en/w/el-isciii-explora-las-posibilidades-de-la-inteligencia-artificial-en-el-abordaje-de-enfermedades-tropicales-desatendidas-1> | N |
| Máster en Dirección de Sistemas y TIC para la Salud y en Digitalización Sanitaria (Master in “Management of Information and Communication Technologies and Healthcare Digitalization”) | Escuela Nacional de Sanidad (ENS) | Spain | Spanish | School/University | Training course | Máster en Dirección de Sistemas y TIC para la Salud | Hybrid | 2020 | Y | N | Y | N | Y | Y | N | N | Master on digital transformation in healthcare: information systems governance, ICT management, data security, interoperability and telemedicine. | <https://seis.es/master-seis-7a-edicion-2020-2021/> | Y |
| Máster en Dirección de Sistemas y TIC para la Salud y en Digitalización Sanitaria (Master in “Management of Information and Communication Technologies and Healthcare Digitalization”) | Escuela Nacional de Sanidad (ENS) | Spain | Spanish | School/University | Training course | Máster en Dirección de Sistemas y TIC para la Salud | Hybrid | 2021 | Y | N | Y | N | Y | Y | N | N | Master on digital transformation in healthcare: information systems governance, ICT management, data security, interoperability, telemedicine and emerging data analytics applications. | <https://seis.es/master-seis-8a-edicion-2021-2022/> | Y |
| Máster en Dirección de Sistemas y TIC para la Salud y en Digitalización Sanitaria (Master in “Management of Information and Communication Technologies and Healthcare Digitalization”) | Escuela Nacional de Sanidad (ENS) | Spain | Spanish | School/University | Training course | Máster en Dirección de Sistemas y TIC para la Salud | Hybrid | 2022 | Y | N | Y | N | Y | Y | N | N | Master on digital transformation in healthcare: information systems governance, ICT management, data security, interoperability, telemedicine and emerging data analytics applications. | <https://seis.es/master-seis-9a-edicion-2022-2023/> | Y |
| Máster en Dirección de Sistemas y TIC para la Salud y en Digitalización Sanitaria (Master in “Management of Information and Communication Technologies and Healthcare Digitalization”) | Escuela Nacional de Sanidad (ENS) | Spain | Spanish | School/University | Training course | Máster en Dirección de Sistemas y TIC para la Salud | Hybrid | 2023 | Y | N | Y | N | Y | Y | N | N | Master on digital transformation in healthcare: information systems governance, ICT management, data security, interoperability, telemedicine, data analytics and AI applications. | <https://seis.es/master-seis-xi-edicion-2024-2025/> | Y |
| Máster en Dirección de Sistemas y TIC para la Salud y en Digitalización Sanitaria (Master in “Management of Information and Communication Technologies and Healthcare Digitalization”) | Escuela Nacional de Sanidad (ENS) | Spain | Spanish | School/University | Training course | Máster en Dirección de Sistemas y TIC para la Salud | Hybrid | 2024 | Y | N | Y | N | Y | Y | N | N | Master on digital transformation in healthcare: information systems governance, ICT management, data security, interoperability, telemedicine, data analytics and AI applications. | <https://seis.es/master-seis-xi-edicion-2024-2025/> | Y |
| Máster en Dirección de Sistemas y TIC para la Salud y en Digitalización Sanitaria (Master in “Management of Information and Communication Technologies and Healthcare Digitalization”) | Escuela Nacional de Sanidad (ENS) | Spain | Spanish | School/University | Training course | Máster en Dirección de Sistemas y TIC para la Salud | Hybrid | 2025 | Y | N | Y | Y | Y | Y | N | N | Master on digital transformation in healthcare: information systems governance, ICT management, data security, interoperability, telemedicine, data analytics and AI applications. | <https://seis.es/master-seis-xii-edicion-2025-2026/> | Y |
| Digital health e AI | Universidad Pública de Navarra (UPNA) | Spain | Spanish | School/University | Seminar/Webinar | Máster en Dirección de Sistemas y TIC para la Salud | Hybrid | 2024 | Y | N | N | N | N | N | N | Y | Hybrid seminar on digital health and AI. | <https://www.unavarra.es/sites/actualidad/contents/noticias/2024/10/03/congreso_inteligencia_artificial.html> | N |
| ¿Cómo incorporar en vigilancia salud pública los cambios tecnológicos?: La inteligencia artificial. | Sociedad Española de Epidemiología (SEE) | Spain | Spanish | Scientific Association/Society | Conference session | X Jornada sobre Vigilancia en Salud Pública | Hybrid | 2024 | Y | N | N | Y | N | N | N | Y | Session on integrating AI and technological changes into public health surveillance. | <https://seepidemiologia.es/wp-content/uploads/2024/05/PROGRAMA_X-Jornada-sobre-Vigilancia-en-Salud-Publica-.-pdf.pdf> | N |
| Health Services Management and Digital Transformation | Escuela Andaluza de Salud Pública (EASP) | Spain | Spanish | School/University | Training course |  | In presence | 2025 | N | N | Y | N | N | Y | N | Y | Second-year specialisation of Europubhealth+ Master on health services management and digital transformation, combining traditional management with AI and digitalisation training. | [https://www.europubhealth.org/second-year-specialisations/healthservicesmanagement/ / https://www.europubhealth.org/wp-content/uploads/2024/10/Syllabus_-Granada-Y2_.pdf](https://www.europubhealth.org/second-year-specialisations/healthservicesmanagement/) | N |
| Datos, evidencia, decisiones: generando valor para la gestión y las políticas sanitarias | Asociación de Economía de la Salud (AES) | Spain | Spanish | Scientific Association/Society | Conference session | XLII Jornadas AES | In presence | 2025 | N | N | Y | Y | Y | N | N | N | Plenary session on data-driven digital transformation in healthcare. | <https://www.aes.es/jornadas/es/> | N |
| Taller IV. Inteligencia artificial | Asociación de Enfermería Comunitaria (AEC) | Spain | Spanish | Scientific Association/Society | Conference session | VIII Congreso Internacional AEC | In presence | 2025 | Y | N | N | N | N | N | N | Y | Workshop on AI. | <https://www.enfermeriacomunitaria.org/web/index.php/congreso2025-programa> | N |
| El futuro de las terapias digitales en el entorno del espacio europeo de datos sanitarios | Asociación Juristas de la Salud (AJS) | Spain | Spanish | Scientific Association/Society | Conference session | XXXIII Congreso Derecho y Salud | In presence | 2025 | Y | N | N | Y | Y | Y | N | Y | Session on the future of digital therapeutics in the European Health Data Space. | <https://www.ajs.es/sites/default/files/2025-04/Programa%20Preliminar%207.pdf> | N |
| Jornada de Formación “Inteligencia Artificial y Gestión Sanitaria” | Sociedad Española de Directivos de la Salud (SEDISA) | Spain | Spanish | Scientific Association/Society | Seminar/Webinar |  | Online | 2025 | Y | N | Y | Y | N | Y | N | Y | Online training day on AI and healthcare management for health executives. | <https://sedisa.net/pagina-eventos/> | N |
| Taller Gestión Sanitaria Basada en Valor. Digitalización de la Gestión del Medicamento en el Hospital | Sociedad Española de Directivos de la Salud (SEDISA) | Spain | Spanish | Scientific Association/Society | Seminar/Webinar |  | Online | 2025 | Y | N | Y | N | Y | Y | N | N | Workshop on value-based healthcare management: digitalisation of hospital medication management. | <https://sedisa.net/pagina-eventos/> | N |
| XXIII Congreso Nacional y XII Internacional de la SEMPSPGS | Sociedad Española de Medicina Preventiva, Salud Pública y Gestión Sanitaria (SEMPSPGS) | Spain | Spanish | Scientific Association/Society | Conference session | XXIII Congreso Nacional y XII Internacional de la SEMPSPGS | In presence | 2025 | Y | N | Y | Y | Y | Y | N | Y | National and international congress with sessions on AI, vaccinations, biosecurity and climate change. | <https://www.congresosempspgspamplona.com/programa/> | N |
| Mesa Redonda: Inteligencia Artificial aplicada a la salud | Sociedad Española de Medicina Preventiva, Salud Pública y Gestión Sanitaria (SEMPSPGS) | Spain | Spanish | Scientific Association/Society | Conference session | XXIII Congreso Nacional y XII Internacional de la SEMPSPGS | In presence | 2025 | Y | N | Y | Y | Y | Y | N | Y | Roundtable on AI in healthcare: implications for management, research and clinical practice. | <https://www.congresosempspgspamplona.com/programa/> | N |
| Eficacia de ChatGPT-4 en vigilancia de infección de localización quirúrgica colorrectal a partir de la historia electrónica | Sociedad Española de Medicina Preventiva, Salud Pública y Gestión Sanitaria (SEMPSPGS) | Spain | Spanish | Scientific Association/Society | Conference session | XXIII Congreso Nacional y XII Internacional de la SEMPSPGS | In presence | 2025 | Y | N | N | Y | Y | N | N | N | Oral communication on ChatGPT-4 efficacy for colorectal surgical site infection surveillance using electronic health records. | <https://www.congresosempspgspamplona.com/programa/> | N |
| R programming and artificial intelligence in biomedical research | Unidad Docente de Medicina Preventiva y Salud Pública - Hospital Clínic Barcelona | Spain | Spanish | School/University | Lecture series | aprile - maggio 2024, ciclo di lezioni per medici in formazione (specializzandi) | In presence | 2024 | N | N | N | Y | N | N | N | N | Lecture series on research methodology applied to AI and R, with practical exercises. | NA | Y |
| MSc Health Data Science | London School of Hygiene & Tropical Medicine (LSHTM) | UK | English | School/University | Degree programme |  | Hybrid | 2020 | Y | N | N | Y | Y | Y | N | Y | MSc in Health Data Science: quantitative and computational programme integrating mathematics, programming, statistics, epidemiology and informatics, including machine learning and EHR analysis. | [https://www.postgrad.com/london-school-of-hygiene-tropical-medicine-university-of-london-interdepartmental-health-data-science/course/ / https://www.lshtm.ac.uk/study/courses/health-and-data-programmes](https://www.postgrad.com/london-school-of-hygiene-tropical-medicine-university-of-london-interdepartmental-health-data-science/course/) | N |
| Training healthcare professionals to work with intelligent robots | Middlesex University | UK | English | School/University | Training course |  | Online | 2020 | Y | N | Y | N | N | N | N | Y | Course training healthcare professionals to work with intelligent robots. | <https://www.digitalhealth.net/2022/11/middlesex-university-leads-training-course-on-working-with-robots/> | N |
| Tech-for-health - why so much is rubbish and what we can do | University of Bristol | UK | English | School/University | Seminar/Webinar | CDT Digital Health Seminar Series | Online | 2020 | Y | N | Y | N | N | N | N | N | Seminar on why many health technologies are ineffective and what can be done. | <https://www.bristol.ac.uk/cdt/digital-health/seminar-series/past-seminars/> | N |
| Diabetes - The Digital era - Hype or Reality? | University of Bristol | UK | English | School/University | Seminar/Webinar | CDT Digital Health Seminar Series | Online | 2020 | Y | N | N | N | N | N | N | N | Seminar on diabetes in the digital era: hype or reality. | <https://www.bristol.ac.uk/cdt/digital-health/seminar-series/past-seminars/> | N |
| Remote patient monitoring - From concept to clinic | University of Bristol | UK | English | School/University | Seminar/Webinar | CDT Digital Health Seminar Series | Online | 2020 | Y | N | N | N | N | N | N | N | Seminar on remote patient monitoring from concept to clinic. | <https://www.bristol.ac.uk/cdt/digital-health/seminar-series/past-seminars/> | N |
| Digital Health & Care at Home - Firm foundations or wishful thinking? | University of Bristol | UK | English | School/University | Seminar/Webinar | CDT Digital Health Seminar Series | Online | 2020 | Y | N | N | N | N | N | N | N | Seminar on digital health and care at home: firm foundations or wishful thinking. | <https://www.bristol.ac.uk/cdt/digital-health/seminar-series/past-seminars/> | N |
| Delivering digital health programmes at scale - hope hype or headache? | University of Bristol | UK | English | School/University | Seminar/Webinar | CDT Digital Health Seminar Series | Online | 2020 | Y | N | Y | N | N | N | N | N | Seminar on delivering digital health programmes at scale: hope, hype or headache. | <https://www.bristol.ac.uk/cdt/digital-health/seminar-series/past-seminars/> | N |
| Connecting Care - Condivisione informazioni tra salute e assistenza sociale | University of Bristol | UK | English | School/University | Seminar/Webinar | CDT Digital Health Seminar Series | Online | 2020 | Y | N | Y | Y | Y | N | N | N | Programme on information sharing between health and social care through new technological solutions. | <https://www.bristol.ac.uk/cdt/digital-health/seminar-series/past-seminars/> | N |
| MSc Health Data Science | London School of Hygiene & Tropical Medicine (LSHTM) | UK | English | School/University | Degree programme |  | Hybrid | 2021 | Y | N | N | Y | Y | Y | N | Y | MSc in Health Data Science: quantitative and computational programme integrating mathematics, programming, statistics, epidemiology and informatics, including machine learning and EHR analysis. | [https://www.postgrad.com/london-school-of-hygiene-tropical-medicine-university-of-london-interdepartmental-health-data-science/course/ / https://www.lshtm.ac.uk/study/courses/health-and-data-programmes](https://www.postgrad.com/london-school-of-hygiene-tropical-medicine-university-of-london-interdepartmental-health-data-science/course/) | N |
| Does my smartphone know I'm sad? - Valutare benessere mentale tramite mobile sensing | University of Bristol | UK | English | School/University | Seminar/Webinar | CDT Digital Health Seminar Series | Online | 2021 | Y | N | N | Y | N | N | N | N | Seminar on assessing mental wellbeing through mobile sensing. | <https://www.bristol.ac.uk/cdt/digital-health/seminar-series/past-seminars/> | N |
| Challenges and Opportunities in Digital Health and Care - working with aliens | University of Bristol | UK | English | School/University | Seminar/Webinar | CDT Digital Health Seminar Series | Online | 2021 | Y | N | Y | N | N | N | N | N | Seminar on challenges and opportunities in digital health and care. | <https://www.bristol.ac.uk/cdt/digital-health/seminar-series/past-seminars/> | N |
| People Drive Digital | University of Bristol | UK | English | School/University | Seminar/Webinar | CDT Digital Health Seminar Series | Online | 2021 | N | Y | Y | N | N | N | N | N | Seminar on how people drive digital. | <https://www.bristol.ac.uk/cdt/digital-health/seminar-series/past-seminars/> | N |
| Operations Research e stato dei dati nell'assistenza sanitaria | University of Bristol | UK | English | School/University | Seminar/Webinar | CDT Digital Health Seminar Series | Online | 2021 | N | N | N | Y | Y | N | N | N | Seminar on operations research modelling and the state of data in healthcare. | <https://www.bristol.ac.uk/cdt/digital-health/seminar-series/past-seminars/> | N |
| Making digital innovation work for the NHS | University of Bristol | UK | English | School/University | Seminar/Webinar | CDT Digital Health Seminar Series | Online | 2021 | Y | N | Y | N | N | N | N | N | Seminar on making digital innovation work for the NHS. | <https://www.bristol.ac.uk/cdt/digital-health/seminar-series/past-seminars/> | N |
| HealthyR: R for healthcare data analysis | Usher Institute - University of Edinburgh | UK | English | School/University | Training course |  | Online | 2021 | N | N | N | Y | N | N | N | N | R training courses for healthcare data analysis, including machine learning (HealthyR). | <https://usher.ed.ac.uk/news-events/events/healthyr> | N |
| MSc Health Data Science | London School of Hygiene & Tropical Medicine (LSHTM) | UK | English | School/University | Degree programme |  | Hybrid | 2022 | Y | N | N | Y | Y | Y | N | Y | MSc in Health Data Science: quantitative and computational programme integrating mathematics, programming, statistics, epidemiology and informatics, including machine learning and EHR analysis. | [https://www.postgrad.com/london-school-of-hygiene-tropical-medicine-university-of-london-interdepartmental-health-data-science/course/ / https://www.lshtm.ac.uk/study/courses/health-and-data-programmes](https://www.postgrad.com/london-school-of-hygiene-tropical-medicine-university-of-london-interdepartmental-health-data-science/course/) | N |
| MSc Health Data Science | London School of Hygiene & Tropical Medicine (LSHTM) | UK | English | School/University | Degree programme |  | Hybrid | 2023 | Y | N | N | Y | Y | Y | N | Y | MSc in Health Data Science: quantitative and computational programme integrating mathematics, programming, statistics, epidemiology and informatics, including machine learning and EHR analysis. | [https://www.postgrad.com/london-school-of-hygiene-tropical-medicine-university-of-london-interdepartmental-health-data-science/course/ / https://www.lshtm.ac.uk/study/courses/health-and-data-programmes](https://www.postgrad.com/london-school-of-hygiene-tropical-medicine-university-of-london-interdepartmental-health-data-science/course/) | N |
| Artificial intelligence and digitalisation of humanitarian assistance | London School of Hygiene & Tropical Medicine (LSHTM) | UK | English | School/University | Seminar/Webinar |  | Online | 2023 | Y | N | N | N | N | N | N | Y | Panel on AI in digitalising humanitarian assistance. | <https://www.lshtm.ac.uk/newsevents/events/artificial-intelligence-and-digitalisation-humanitarian-assistance> | N |
| A Systematic Review - Exploring the Ways Healthcare Teams Facilitate Patient Uptake and Engagement with Digital Health Interventions | Society for Social Medicine & Population Health | UK | English | Scientific Association/Society | Conference session | SSM Annual Scientific Meeting | In presence | 2023 | Y | Y | N | N | N | N | N | N | Systematic review on how healthcare teams facilitate patient uptake and engagement with digital health interventions. | <https://cdn.eventsforce.net/files/ef-px6ci6a56tzs/website/247/ssm_handbook_2023.pdf> | N |
| Technology and Systems - What do they mean for public health? | University of Cambridge | UK | English | School/University | Seminar/Webinar |  | Online | 2023 | Y | N | Y | N | N | N | N | N | Seminar on what technology and systems mean for public health. | <https://www.cph.cam.ac.uk/events/technology-and-systems-what-do-they-mean-public-health> | N |
| New Horizons in Public Health - The use of AI in public health in the UK | Faculty of Public Health (FPH) | UK | English | Scientific Association/Society | Seminar/Webinar |  | Online | 2024 | Y | N | N | N | N | N | N | Y | Event on AI use in public health in the UK. | <https://www.fph.org.uk/policy-advocacy/special-interest-groups/artificial-intelligence-digital-public-health-sig/> | N |
| FPH Public Health Film SIG - International Public Health Film Competition (2024) | Faculty of Public Health (FPH) | UK | English | Scientific Association/Society | Other |  | Hybrid | 2024 | N | Y | N | N | N | N | N | Y | International film competition with focus on AI's influence on public health, including award for 'Best film on AI and Public Health'. Innovative approach to training using creative media. | <https://www.fph.org.uk/policy-advocacy/special-interest-groups/public-health-film-sig/> | N |
| MSc Health Data Science | London School of Hygiene & Tropical Medicine (LSHTM) | UK | English | School/University | Degree programme |  | Hybrid | 2024 | Y | N | N | Y | Y | Y | N | Y | MSc in Health Data Science: quantitative and computational programme integrating mathematics, programming, statistics, epidemiology and informatics, including machine learning and EHR analysis. | [https://www.postgrad.com/london-school-of-hygiene-tropical-medicine-university-of-london-interdepartmental-health-data-science/course/ / https://www.lshtm.ac.uk/study/courses/health-and-data-programmes](https://www.postgrad.com/london-school-of-hygiene-tropical-medicine-university-of-london-interdepartmental-health-data-science/course/) | N |
| Advancing vaccine equity with big data and digital health tools | London School of Hygiene & Tropical Medicine (LSHTM) | UK | English | School/University | Seminar/Webinar |  | Online | 2024 | N | Y | N | Y | N | N | N | N | Seminar on advancing vaccine equity with big data and digital health tools. | <https://www.lshtm.ac.uk/newsevents/events/advancing-vaccine-equity-big-data-and-digital-health-tools> | N |
| Incorporating novel and digital interventions into sexual health services in Australia | London School of Hygiene & Tropical Medicine (LSHTM) | UK | English | School/University | Seminar/Webinar |  | Online | 2024 | Y | N | N | N | N | N | N | N | Seminar on incorporating digital interventions into sexual health services (Australian experience). | <https://www.lshtm.ac.uk/newsevents/events/incorporating-novel-and-digital-interventions-sexual-health-services-australia> | N |
| AI and digital transformation in healthcare | University of Cambridge | UK | English | School/University | Training course |  | Online | 2024 | Y | N | Y | Y | N | N | N | Y | Course on AI and digital transformation in healthcare. | <https://www.ice.cam.ac.uk/course/ai-and-digital-transformation-healthcare> | N |
| Compassionate Digital Integrated Care | University of Salford - School of Health and Society | UK | English | School/University | Training course |  | Online | 2024 | Y | Y | Y | N | N | Y | N | N | Module for community healthcare professionals on compassionate use of technology to improve care delivery, prevention, independent living and self-care. | <https://www.salford.ac.uk/courses/single-module/compassionate-digital-integrated-care-15-credit-level-7> | N |
| Tomorrow's Health Today - AI and Data Science explained | Cambridge Public Health | UK | English | School/University | Seminar/Webinar |  | Hybrid | 2025 | N | N | N | Y | N | N | N | Y | Event explaining AI and data science for tomorrow's health. | <https://www.cph.cam.ac.uk/news/tomorrows-health-today-ai-and-data-science-explained> | N |
| Digital Health Competencies Launch Event | King's College London | UK | English | School/University | Seminar/Webinar |  | Hybrid | 2025 | N | Y | Y | N | N | N | N | N | Launch event on digital health competencies and curricula importance for medical education, following DECODE framework publication. | <https://www.kcl.ac.uk/events/digital-health-competencies-launch-event> | N |
| MSc Health Data Science | London School of Hygiene & Tropical Medicine (LSHTM) | UK | English | School/University | Degree programme |  | Hybrid | 2025 | Y | N | N | Y | Y | Y | N | Y | MSc in Health Data Science: quantitative and computational programme integrating mathematics, programming, statistics, epidemiology and informatics, including machine learning and EHR analysis. | [https://www.postgrad.com/london-school-of-hygiene-tropical-medicine-university-of-london-interdepartmental-health-data-science/course/ / https://www.lshtm.ac.uk/study/courses/health-and-data-programmes](https://www.postgrad.com/london-school-of-hygiene-tropical-medicine-university-of-london-interdepartmental-health-data-science/course/) | N |
| AI in Health: Introduction to Key Concepts and Applications | London School of Hygiene & Tropical Medicine (LSHTM) | UK | English | School/University | Training course |  | In presence | 2025 | Y | N | N | Y | N | Y | N | Y | Two-day introductory course on AI in health: technical fundamentals (ML, DL, computer vision, LLMs), practical applications (hands-on exercises, AI evaluation, critical literature appraisal) and ethical considerations. 11 CPD credits from Royal College of Physicians. | <https://www.lshtm.ac.uk/study/courses/short-courses/AI-health> | Y |
| Digital Innovation Leaders Forum - AI Workshop | NHS Confederation | UK | English | Scientific Association/Society | Seminar/Webinar | Digital Innovation Leaders Forum | Online | 2025 | Y | N | Y | N | N | Y | N | Y | Workshop for NHS digital and innovation leaders exploring AI use cases, adoption barriers and solutions. | <https://www.nhsconfed.org/events/digital-innovation-leaders-forum-ai-workshop> | N |
| AI in the NHS 2025 | The Health Foundation | UK | English | Scientific Association/Society | Seminar/Webinar |  | Online | 2025 | Y | N | Y | N | N | Y | N | Y | Online event on assessing new AI uses, determining safety and efficacy, and understanding adoption requirements at scale in the NHS. | <https://www.health.org.uk/events/ai-in-the-nhs-2025> | N |
| Master / MSc - Artificial Intelligence for Public Health (AI4PH) | Institut des Sciences de la Santé Publique d'Aix-Marseille Université (ISSPAM) | France | English; French | School/University | Degree programme |  | Online | 2025 | Y | N | N | Y | N | Y | N | Y | Master/MSc programme on AI for public health. | <https://sesstim.univ-amu.fr/fr/master-ai4ph> | Y |
| Diplôme d’Etudes Supérieures Universitaires/Postgraduate Diploma - Artificial Intelligence for Public Health (AI4PH) | Institut des Sciences de la Santé Publique d'Aix-Marseille Université (ISSPAM) | France | English | School/University | Training course |  | Online | 2025 | Y | N | N | Y | Y | N | N | Y | Postgraduate diploma on AI for public health. | <https://sesstim.univ-amu.fr/fr/desu-ai4ph> | Y |
| Intelligence artificielle explicable pour le cancer du sein : raisonnement à partir de cas visuel. | Sciences Économiques et Sociales de la Santé et Traitement de l'Information Médicale (SESSTIM) | France | French | School/University | Seminar/Webinar |  | Online | 2020 | Y | N | N | Y | N | N | N | Y | Webinar on explainable AI for breast cancer using visual case-based reasoning. | <https://sesstim.univ-amu.fr/fr/node/16522> | Y |
| Réutilisation de données hospitalières et intelligence artificielle : des données à l'intervention de santé, un chemin cahoteux. | Sciences Économiques et Sociales de la Santé et Traitement de l'Information Médicale (SESSTIM) | France | French | School/University | Seminar/Webinar |  | Online | 2020 | Y | N | N | Y | Y | N | N | N | Webinar on reusing hospital data with AI: from data to health intervention. | <https://sesstim.univ-amu.fr/fr/node/16318> | Y |
| Data, Personalization, Digital Health! | Sciences Économiques et Sociales de la Santé et Traitement de l'Information Médicale (SESSTIM) | France | English | School/University | Seminar/Webinar |  | Online | 2021 | Y | N | N | Y | N | N | N | N | Seminar on data, personalisation and digital health. | <https://sesstim.univ-amu.fr/fr/node/16622> | Y |
| Données massives et intelligence artificielle en médecine : espoirs et défis | Sciences Économiques et Sociales de la Santé et Traitement de l'Information Médicale (SESSTIM) | France | French | School/University | Seminar/Webinar |  | Online | 2022 | N | N | N | Y | N | N | N | Y | Webinar on opportunities and challenges of AI and big data in medicine. | <https://sesstim.univ-amu.fr/fr/video-box/webinar-quantim-christian-lovis> | Y |
| Machine learning for health: promises and methodological challenges | Sciences Économiques et Sociales de la Santé et Traitement de l'Information Médicale (SESSTIM) | France | English | School/University | Seminar/Webinar |  | Online | 2024 | N | N | N | Y | N | N | N | Y | Seminar on machine learning for health: promises and methodological challenges. | <https://sesstim.univ-amu.fr/fr/video-box/webinar-quantim-gael-varoquaux> | Y |
| The importance of transparency in predictive AI: the role of reporting guidelines | Sciences Économiques et Sociales de la Santé et Traitement de l'Information Médicale (SESSTIM) | France | English | School/University | Seminar/Webinar |  | Online | 2025 | N | N | N | Y | N | N | N | Y | Seminar on transparency in predictive AI and the role of reporting guidelines. | <https://sesstim.univ-amu.fr/fr/video-box/webinar-quantim-gary-collins> | Y |
| Methods and Challenges in Public Health Sciences | Institut des Sciences de la Santé Publique d'Aix-Marseille Université (ISSPAM) | France | English; French | School/University | Summer/Winter/Spring school |  | Hybrid | 2024 | N | N | N | Y | Y | N | N | N | Summer school on methods and challenges in public health sciences, including machine learning and programming fundamentals. | <https://www.univ-amu.fr/en/public/summer-school-isspam-2024-methods-and-challenges-public-health-sciences> | Y |
| Methods and Challenges in Public Health Sciences | Institut des Sciences de la Santé Publique d'Aix-Marseille Université (ISSPAM) | France | English; French | School/University | Summer/Winter/Spring school |  | Hybrid | 2025 | N | N | N | Y | Y | N | N | N | Summer school (12h lectures, 6h practical): machine learning methods and programming fundamentals for health data. | <https://institut-isspam.univ-amu.fr/en/training/summer-school/summer-school-2025> | Y |
| Perspectives offered by the reuse of healthcare data | Institut des Sciences de la Santé Publique d'Aix-Marseille Université (ISSPAM); Isped - School of Public Health - Université de Bordeaux | France | English | School/University | Seminar/Webinar | Joint ISSPAM / ISPED webinar: Perspectives offered by the reuse of healthcare data | Online | 2025 | N | N | N | N | Y | N | N | N | Seminar on healthcare data reuse: health data in France, pharmaceutical validation with hospital data (PharmIAge), AI transformers in SNDS for EHDS pilot project. | <https://institut-isspam.univ-amu.fr/en/training/summer-school/summer-school-2025> | Y |
| Master Public Health Data Science (M2 PHDS) | Isped - School of Public Health - Université de Bordeaux | France | English | School/University | Degree programme | Master Public Health Data Science (M2 PHDS) | Hybrid | 2024 | Y | Y | N | Y | Y | N | N | N | Master training future digital public health leaders (e.g. chief data scientists) through a unique European programme linking research and training. | <https://www.isped.u-bordeaux.fr/Graduate-Programs/Digital-Public-Health/Masters-Program> | Y |
| Master Public Health Data Science (M2 PHDS) | Isped - School of Public Health - Université de Bordeaux | France | English | School/University | Degree programme | Master Public Health Data Science (M2 PHDS) | Hybrid | 2025 | Y | N | N | Y | Y | N | N | N | Master training future digital public health leaders (e.g. chief data scientists) through a unique European programme linking research and training. | <https://www.isped.u-bordeaux.fr/Graduate-Programs/Digital-Public-Health/Masters-Program> | Y |
| University diploma Public Health Data Sciences | Isped - School of Public Health - Université de Bordeaux | France | English | School/University | Training course | Master Public Health Data Science (M2 PHDS) | Online | 2025 | Y | Y | N | Y | Y | N | N | N | Professional certificate (Diplôme Universitaire) on leveraging data for population health. Includes modules on digital tools for data sciences (30h) and health informatics fundamentals (45h). | <https://www.isped.u-bordeaux.fr/Graduate-Programs/Digital-Public-Health/DU-Public-Health-Data-Sciences> | Y |
| Master en science des données de santé publique | Isped - School of Public Health - Université de Bordeaux | France | French | School/University | Degree programme |  | Hybrid | 2024 | Y | Y | N | Y | Y | N | N | N | Second-year Master in Public Health Data Science: one semester coursework (300h) plus internship. Combines epidemiology, statistics and data analysis with professional experience. Dual degree option with McGill University. | <https://www.isped.u-bordeaux.fr/Graduate-Programs/Digital-Public-Health/Programme-en-fran%C3%A7ais> | N |
| Master en science des données de santé publique | Isped - School of Public Health - Université de Bordeaux | France | French | School/University | Degree programme |  | Hybrid | 2025 | Y | Y | N | Y | Y | N | N | N | Second-year Master in Public Health Data Science: one semester coursework (300h) plus internship. Combines epidemiology, statistics and data analysis with professional experience. Dual degree option with McGill University. | <https://www.isped.u-bordeaux.fr/Graduate-Programs/Digital-Public-Health/Programme-en-fran%C3%A7ais> | N |
